# Multi-omics approaches identify a key gene, *PpTST1*, for organic acid accumulation in peach

**DOI:** 10.1093/hr/uhac026

**Published:** 2022-02-19

**Authors:** Qi Wang, Ke Cao, Lailiang Cheng, Yong Li, Jian Guo, Xuanwen Yang, Jiao Wang, Irshad Ahmad Khan, Gengrui Zhu, Weichao Fang, Changwen Chen, Xinwei Wang, Jinlong Wu, Qiang Xu, Lirong Wang

**Affiliations:** Zhengzhou Fruit Research Institute, Chinese Academy of Agricultural Sciences, Zhengzhou 450009, China; College of Horticulture & Forestry Sciences, Huazhong Agricultural University, Wuhan, China; Zhengzhou Fruit Research Institute, Chinese Academy of Agricultural Sciences, Zhengzhou 450009, China; Horticulture Section, School of Integrative Plant Science, Cornell University, Ithaca, NY 14853, USA; Zhengzhou Fruit Research Institute, Chinese Academy of Agricultural Sciences, Zhengzhou 450009, China; Zhengzhou Fruit Research Institute, Chinese Academy of Agricultural Sciences, Zhengzhou 450009, China; Zhengzhou Fruit Research Institute, Chinese Academy of Agricultural Sciences, Zhengzhou 450009, China; Zhengzhou Fruit Research Institute, Chinese Academy of Agricultural Sciences, Zhengzhou 450009, China; Zhengzhou Fruit Research Institute, Chinese Academy of Agricultural Sciences, Zhengzhou 450009, China; Zhengzhou Fruit Research Institute, Chinese Academy of Agricultural Sciences, Zhengzhou 450009, China; Zhengzhou Fruit Research Institute, Chinese Academy of Agricultural Sciences, Zhengzhou 450009, China; Zhengzhou Fruit Research Institute, Chinese Academy of Agricultural Sciences, Zhengzhou 450009, China; Zhengzhou Fruit Research Institute, Chinese Academy of Agricultural Sciences, Zhengzhou 450009, China; Zhengzhou Fruit Research Institute, Chinese Academy of Agricultural Sciences, Zhengzhou 450009, China; College of Horticulture & Forestry Sciences, Huazhong Agricultural University, Wuhan, China; Zhengzhou Fruit Research Institute, Chinese Academy of Agricultural Sciences, Zhengzhou 450009, China

## Abstract

Organic acid content in fruit is an important determinant of peach organoleptic quality, and undergoes considerable variations during development and maturation. However, its molecular mechanism remains largely unclear. In this study, an integrative approach of genome-wide association studies and comparative transcriptome analysis was applied to identify candidate genes involved in organic acid accumulation in peach. A key gene, *PpTST1*, encoding tonoplast sugar transporter, was identified and the genotype of *PpTST1* with a single-base transversion (G1584T) in the third exon that leads to a single amino acid substitution (Q528H) was associated with a low level of organic acid content in peach. Overexpression of *PpTST1*^*His*^ resulted in reduced organic acid content along with increased sugar content both in peach and tomato fruits, suggesting its dual function in sugar accumulation and organic acid content reduction. Two V-type proton ATPases interacted with PpTST1 in a yeast two-hybrid assay. In addition, the G1584T transversion appeared and gradually accumulated during domestication and improvement, which indicated that *PpTST1* was under selection. The identification and characterization of *PpTST1* would facilitate the improvement of peach fruit quality.

## Introduction

Peach, as one of the most commercially important Rosaceae trees, is cultivated widely in the world due to its unique flavor and abundant nutrients, with total production of ~19 million tons (FAO, http://www.fao.org/faostat/en/#data/QC). The edible peach emerged 3.47–2.6 million years ago [1]. More recently during its domestication and subsequent breeding efforts there has been selection for improved fruit quality [2]. Genomic analysis not only revealed the breeding history of peach [2, 3], but also provided many molecular markers associated with fruit quality [4, 5], which have been slowly translated into routine practical application in plant breeding [6, 7]. However, traditional fruit breeding programs have focused their efforts on improving fruit external and textural attributes of peaches and postharvest handling and storage attributes [8]. Therefore many commercial peach cultivars lack high flavor [9]. Developing more novel peach cultivars with excellent taste is a main breeding objective to reverse market inertia.

Organic acids, such as malate, citrate, and quinate, have a strong influence on organoleptic fruit quality and are crucial components involved in the development of fruit flavor [10]. Malate and citrate are the major organic acids in mature peach (*Prunus persica* (L.) Batsch) fruit as well as in many other fleshy fruits [11]. Interactions between metabolism and vacuolar storage contribute to the accumulation of these two acids in fruit cells [12]. Concerning malate, Bai *et al*. [13] indicated that *Ma1*, an aluminum-activated malate transporter like (ALMT-like) gene, could be the main determinant of malate content in apple fruit. Differences in fruit acidity were possibly caused not only through a single nucleotide mutation at base 1455 in the open reading frame (ORF), but also by differences in expression levels of *Ma1* [13, 14]. Further evidence held Ma1 responsible for the content of malic acid, and its conserved C-terminal domain for malate transport was identified [15]. Moreover, significant differences in malic acid content in ripe fruits were also detected between accessions with the same genotype at the *Ma* locus, suggesting that other genetic determinants of fruit acidity existed in apple [16]. Hu *et al*. [17, 18] demonstrated that two R2R3-MYB transcription factors, MYB1 and MYB73, influenced malate accumulation and vacuolar pH by activating vacuolar transporters. It was revealed that the MdSAUR37/MdPP2CH/MdALMTII chain accurately determined fruit malate content in apple though cascading hierarchical epistatic genetic effects [19]. A P-type ATPase encoded by *Ma10* was identified to promote malate uptake into the vacuole [20]. MdBT2 was reported to regulate malate accumulation and vacuolar acidification in response to nitrate [21]. MdbHLH3 was found to modulate the accumulation of malate by binding to the promoter of *MdcyMDH* [22]. In regard to citrate, CsPH8 [23], CrMYB73 [24], and CitERF13 [25] were suggested to regulate citrate accumulation in citrus fruit. CitNAC62, with CitWRKY1, is involved in citric acid degradation though up-regulating *CitAco3* [26]. Strazzer *et al*. [27] found that a vacuolar proton-pumping P-ATPase complex participated in hyperacidification of citrus fruits and that its loss of expression leads to the loss of acidification in citrus fruits. The inactivating mutations in the examined genotypes were located in upstream transcription regulators. In some genotypes the inactivating mutation occurred in a basic helix-loop-helix (bHLH) gene, which Stazzer *et al*. [27] called *CitAN1* and Butelli *et al*. [28] called *Noemi*, that activated the P-ATPase proton pump genes.

In peach, previous studies revealed that the major locus (*D*/*d*) controlling the low-acid trait [characterized by high pH, low titratable acidity (TA) or reduced contents of malic and citric acids] and quantitative trait loci (QTLs) taking part in regulating peach fruit acidity are related to dominant markers on linkage group 5 [29, 30]. The corresponding physical location of the *D* locus (characterized by high pH and low TA) estimated through linkage analysis is 772–994 kb at the top of chromosome 5 [31]. After analyzing the relation between CPPCT040 alleles [simple sequence repeat (SSR)] and TA values, the authors found that the probability of the allele CPPCT040^193^ was higher (>91%) in varieties with TA ≤5 g/l while this probability reduced rapidly with increasing TA. A single-nucleotide polymorphism (SNP) marker linked to the SSR was also developed [32]. Our previous study detected a significant region on chromosome 5 and identified that a candidate gene, *Prupe.5G004300*, as a C to T substitution ~11.0 kb upstream of its start codon, co-segregates with the low-acid trait [4, 33], which was more highly expressed in acidic fruits than low-acid fruits at mature stage. Nevertheless, on the strength of dynamic changes in total organic acid in previous studies [4, 34], the low-acid trait resulted from decrease in total organic acid content during the period from the second exponential growth to mature stage in low-acid accessions [34]. The high expression of *Prupe.5G004300* might positively regulate total acid at mature stage, but it could not be explained why the level of total organic acid is high with low expression of *Prupe.5G004300* at early fruit developmental stages [4]. Recently, an increase in pH and a decrease in the concentrations of citrate and malate were induced by overexpression of *PpRPH* (*Prupe.5G008400*) in tobacco leaves [35]. However, further studies are needed to illustrate the role of *PpRPH* in organic acid accumulation in peach.

Here, through an integrative analysis of genome-wide association studies (GWAS), transcriptome analysis, and transient overexpression, we identified a key gene, *PpTST1*, encoding a tonoplast sugar transporter, with two alleles associated with level of organic acid content. Up-regulation of *PpTST1* was seen during the later stage of fruit development in both ‘Tianjin Shui Mi’ (acidic) and ‘Hakuho’ (low-acid). Furthermore, overexpression of *PpTST1^His^* resulted in reduced acid content as well as increased sugar accumulation in both peach and tomato. The identification of *PpTST1* and the mutated allele will facilitate the improvement of peach fruit flavor.

## Results

### GWAS identified SNPs significantly associated with organic acid accumulation in peach

**Figure 1:**
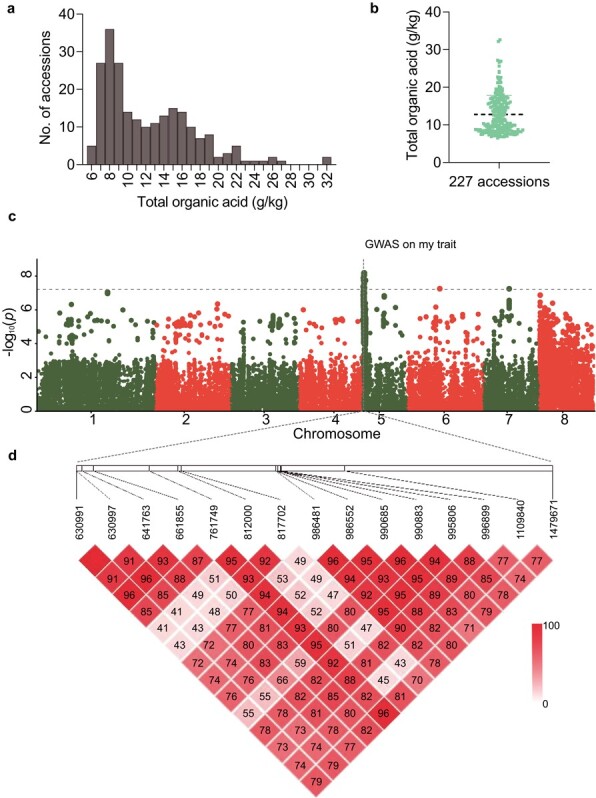
GWAS identified QTLs associated with organic acid accumulation in peach fruit. **a** Distribution of fruit organic acid contents in the 227 accessions measured over three consecutive years (2013–2015). **b** Distribution of fruit organic acid contents in the 227 accessions used for GWAS. **c** Manhattan plot of the GWAS results for fruit organic acid content with a compressed MLM. The vertical axis presents negative log10-transformed *P*-values from the compressed MLM. The horizontal dashed line represents the Bonferroni significance threshold of GWAS. **d** LD plot of 15 significant association signals in a 0.85-Mb (0.63–1.48 Mb) interval on the top of chromosome 5. In each box, the color represents the relationship of LD and the number represents the value of LD multiplied by 100. Absence of a number from a box means the value of LD is 1.

In this study, we measured total organic acids (TOA) of 227 accessions ranging from 6.52 to 32.62 g/kg (Fig. 1a and b; Supplementary Data Table S1). The 227 accessions (Supplementary Data Table S1) were used for GWAS using MLM with principal component analysis (PCA) and kinship analysis, which determine population structure and family pertinence. Our results showed that a total of 15 SNPs exceeding the significant threshold were determined as significant association signals (Fig. 1c; Supplementary Data Table S2). Because the 15 significant association signals were linked with each other [79.0% of the boxes with linkage disequilibrium (LD) >.7] (Fig. 1d), the region range from 0.63 to 1.48 Mb on the top of chromosome 5 was considered as the candidate interval which was overlapped with the known D locus. In the candidate interval, 84 annotated genes were detected (Supplementary Data Table S3).

### Organic acid accumulation during fruit development and transcriptome analysis

**Figure 2:**
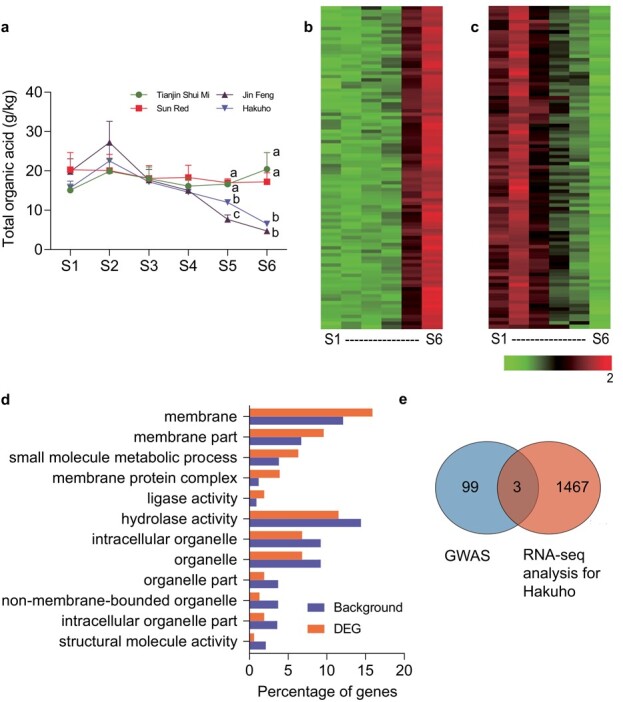
Identification of key candidates associated with organic acid accumulation through an integrative approach using GWAS and transcriptome analysis. **a** Changes in the concentration of total organic acid throughout fruit development (20, 40, 60, 80, 100, and 120 d after blooming) of low-acid and acidic peach accessions. Significant differences among cultivars are shown by different lowercase letters (*P* < .01, Student’s *t*-test), while the stages of fruit development not marked indicate no significant difference among cultivars at the same stage. Error bars indicate the standard error of three biological replicates. S1–S6, stages 1–6. **b**, **c** Heat map with color range from −2 to 2 of the DEGs up-regulated or down-regulated simultaneously at stages 5 and 6 compared with stages 1–4 in ‘Hakuho’. The RPKM value of each gene at six developmental stages was normalized to the range from −2 to 2. **d** GO enrichment analysis of 1470 DEGs up-regulated or down-regulated simultaneously at stages 5 and 6 compared with stages 1–4 in ‘Hakuho’. **e** Venn diagram of the number of genes detected through GWAS and transcriptome analysis.

‘Hakuho’ (low acid) and ‘Tianjin Shui Mi’ (acidic) were selected to the determine dynamic change trend of organic acid contents in peach fruits during six developmental stages, 20, 40, 60, 80, 100, and 120 days after blooming, respectively. ‘Hakuho’ had the maximum and minimum accumulation of organic acid at S2 and S6, respectively. Significant differences in organic acid contents between ‘Hakuho’ and ‘Tianjin Shui Mi’ occurred at S5 and S6 (Fig. 2a).

To identify differentially expressed genes (DEGs) associated with organic acid accumulation in peach fruit, RNA-seq assays were conducted at the six developmental stages for ‘Hakuho’ and ‘Tianjin Shui Mi’. After removal of adapter and low-quality reads, a total of 56.6 Gb (92.5%) of clean data were obtained from 24 libraries. More than 89.8 and 89.7% of reads from ‘Hakuho’ and ‘Tianjin Shui Mi’ were mapped to the peach genome, respectively (Supplementary Data Table S4-1). The most significant difference in organic acid content happened between S2 and S6 in ‘Hakuho’. The comparative transcriptome analysis between S2 and S6 in ‘Hakuho’ identified 7807 DEGs (fold change >2) associated with organic acid accumulation.

Significant differences in organic acid content between ‘Hakuho’ and ‘Tianjin Shui Mi’ during S5 and S6 were detected (Fig. 2a). DEGs showing up- or down-regulation during both S5 and S6 as compared with S1–S4 were selected (Fig. 2b and c; Supplementary Data Table S3) for a gene ontogeny (GO) enrichment analysis. The 1470 DEGs identified revealed a higher percentage of genes that were enriched in ‘membrane’, ‘membrane part’, and ‘membrane protein complex’ terms (Fig. 2d), suggesting that regulation of organic acid accumulation may occur on the membranes.

### Bulk segregant analysis sequencing

**Figure 3:**
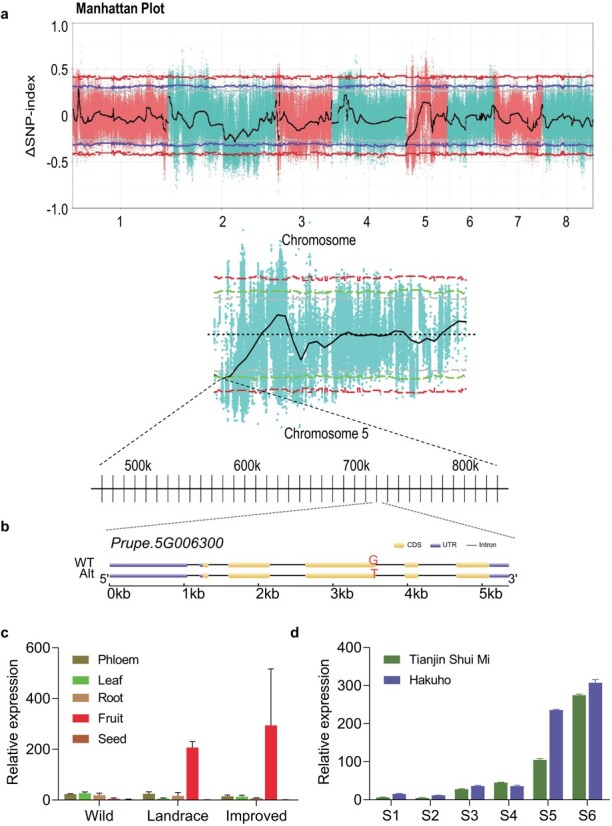
Location, structure, and expression analysis of *PpTST1*. **a** The *Δ*SNP index with its 90, 95, and 99% confidence intervals represented by gray, blue, and red lines, respectively. The *Δ*SNP index is the difference in SNP index in the two pools. The SNP index is defined as the ratio between the number of reads of a mutant SNP and the total number of reads corresponding to the SNP. **b** Structural variations of *PpTST1* haplotypes (WT represents the same sequence and Alt represents a sequence different from the reference genome). The promoter and 3′ untranslated region (UTR) are represented by blue boxes, coding sequences are represented by yellow boxes, and introns are represented by the thin lines between boxes. The corresponding positions of the nucleotide polymorphism (G1584T) are marked. **c** Tissue-specific expression of *PpTST1* in wild related species [‘Zhou Xing Shan Tao’ (acidic), ‘Hong Gen Gan Su Tao’ (acidic), and ‘A Ba Gung He Tao’ (acidic)], edible landraces [‘Nanshan Tian Tao’ (low acid), ‘Huo Lian Jin Dan’ (acidic), and ‘Chinese Cling’ (acidic)], and improved varieties [‘96-2-51’ (low acid), ‘Yu Lu’ (low acid), and ‘Fantasia’ (acidic)]. Error bars indicate the standard error of three biological replicates. **d** Expression profile of *PpTST1* in ‘Hakuho’ (low acid) and ‘Tianjin Shui Mi’ (acidic) at six developmental stages (S1–S6), 20, 40, 60, 80, 100, and 120 days after blooming. Error bars indicate the standard error of two biological replicates.

To further confirm our results, bulk segregant analysis sequencing (BSA-seq) was performed. A total of 21.22 and 21.08 Gb of clean data was generated for the low-acid and acidic bulks, and the sequencing depth was 68.74-fold and 69.18-fold, respectively (referring to the ‘Lovell’ genome) (Supplementary Data Table S4-2). A total of 1 731 344 SNPs were detected across all eight chromosomes. Against the genome positions, we calculated and plotted a *Δ*SNP-index graph, and only one locus exceeded a 95% confidence interval, which was located in a 0.36 Mb (0.47–0.83 Mb) interval on chromosome 5 (Fig. 3a). An interval of 0.16 Mb (0.47–0.63 Mb) was not in the candidate interval obtained from GWAS. There are 20 genes (*Prupe.5G003400*–*Prupe.5G005300*) in this 0.16-Mb region. However all the 20 genes were not selected by transcriptome analysis. There were three loci that exceeded 90% confidence intervals, which were located to a 0.56-Mb (3.68–4.24 Mb, *Prupe.1G052700*–*Prupe.1G060200*) interval on chromosome 1, a 0.25-Mb (10.40–10.65 Mb) interval, and a 0.31-Mb (12.65–12.96 Mb, *Prupe.2G081600*–*Prupe.2G082300*) interval on chromosome 2, respectively. A total of 84 genes fell in these three loci, among which 3 genes (*Prupe.1G054400*, *Prupe.1G056800*, and *Prupe.1G056900*) were also selected by transcriptome analysis and will be studied further. In this study, we focused on the most significant locus, which might have the main effect on organic acid accumulation.

### *PpTST1* is the candidate gene for organic acid accumulation

To investigate the causal gene responsible for organic acid accumulation, two analytical strategies—GWAS and comparative transcriptome analysis—were carried out, resulting in three common genes (*Prupe.5G006300, Prupe.5G006400*, and *Prupe.5G009600*) (Fig. 2e). There were no association signals (*P* < 1 × 10^−6^) in the locus of *Prupe.5G006400* (5 g: 721835–724817) and *Prupe.5G009600* (5 g: 990843–994 898) (Fig. 1d). Transient overexpression of the three genes was performed in peach fruit. The results revealed that overexpression of *Prupe.5G006400* and *Prupe.5G009600* had no significant effect on the accumulation of total organic acid and the TA value (Supplementary Data Fig. S1). Therefore only the gene *Prupe.5G006300*, designated *PpTST1*, encoding tonoplast sugar transporter [36], was considered as the candidate gene. Further, we used five wild (‘2010 Tibet 32’, ‘2010 Tibet 54’, ‘Julong 13’, ‘Lawu 1’, and ‘Yan Tian Jing Qu Zao Hua Tao’), two landrace (‘Qiu Bai Tao’, and ‘Xiao Jin Dan’), and two improved (‘Hang Zhou Zao Shui Mi’, and ‘Jin Feng’) varieties to carry out sequence alignments of *PpTST1*, which revealed 22 polymorphisms in the coding sequence (Supplementary Data Fig. S2). However only a single-base transversion (G1584T) in the third exon, defined as dCAPS1584, which led to a single amino acid substitution (Q528H), was significantly associated with the contents of organic acid. This might be responsible for organic acid accumulation (Fig. 3b; Supplementary Data Table S2).

The tissue-specific expression of *PpTST1* was carried out in wild related species, edible landraces, and improved varieties. Relatively higher expression of *PpTST1* was seen in fruit compared with other tissues in all peach species except wild related species (Fig. 3c). The expression of *PpTST1* showed an elevated increase at stage 5 and stage 6 in ‘Hakuho’ (low acid) and ‘Tianjin Shui Mi’ (acidic) (Fig. 3d), indicating *PpTST1* had specific expression in fruit and high expression at stages 5 and 6 in both low-acid and acidic accessions.

### Overexpression of *PpTST1^His^* reduced organic acid accumulation in peach and tomato fruit

**Figure 4:**
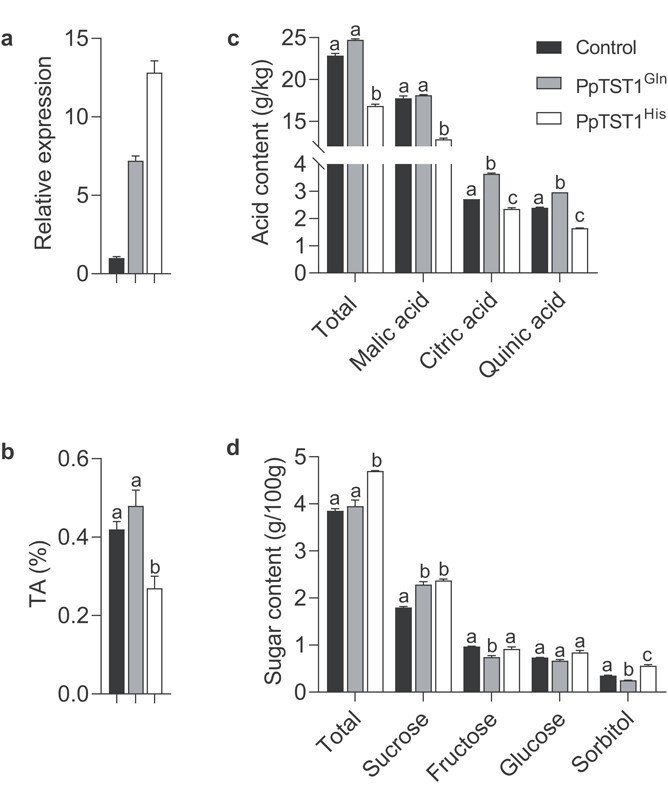
Transient overexpression assays. **a** Relative expression levels of *PpTST1* in fruits from transiently transformed peaches and the control by qRT–PCR. *PpActin* (*Prupe.6G163400*) was used as housekeeping gene. Gene expression was normalized against *PpActin* as an internal expression control. The control was set at 1 to calculate the relative expression of transformants. **b** TA values in transiently transformed peaches and the control. **c** Total and three main organic acid contents in transiently transformed peaches and the control. **d** Total and four main sugar contents in transiently transformed peaches and the control. Significant differences among cultivars are shown by different lowercase letters (*P* < .01, Student’s *t*-test). Error bars present the standard error of three biological replicates.

To investigate the function of *PpTST1* in regulating organic acid accumulation in peach, the overexpression vectors 35S::*PpTST1^His^*, 35S::*PpTST1^Gln^* constructs, and the empty vector were transiently transformed into ‘Tianjin Shui Mi’ (acidic) fruits at stage 5. Compared with the control, the *PpTST1* transcript increased 6- to 12-fold in fruit transformed with the overexpression vectors, indicating the overexpression of *PpTST1^His^* and *PpTST1^Gln^* (Fig. 4a). The TA level of fruit transformed with 35S::*PpTST1^His^* was decreased by 35.7%, and that of fruit transformed with 35S::*PpTST1^Gln^* had no significant change, relative to the control (Fig. 4b). High-performance liquid chromatography (HPLC) analysis then showed that overexpression of *PpTST1^His^* decreased the contents of total organic acid, malic acid, citric acid, and quinic acid by 26.3, 27.7, 13.2, and 31.2%, respectively. For transgenic fruits of *PpTST1^Gln^*, the contents of total organic acid and malic acid remained the same as in the wild type, while the contents of citric acid and quinic acid increased by 34.3 and 23.6%, respectively (Fig. 4c). These results indicated that overexpression of *PpTST1^His^* can reduce organic acid accumulation in peach.

**Figure 5:**
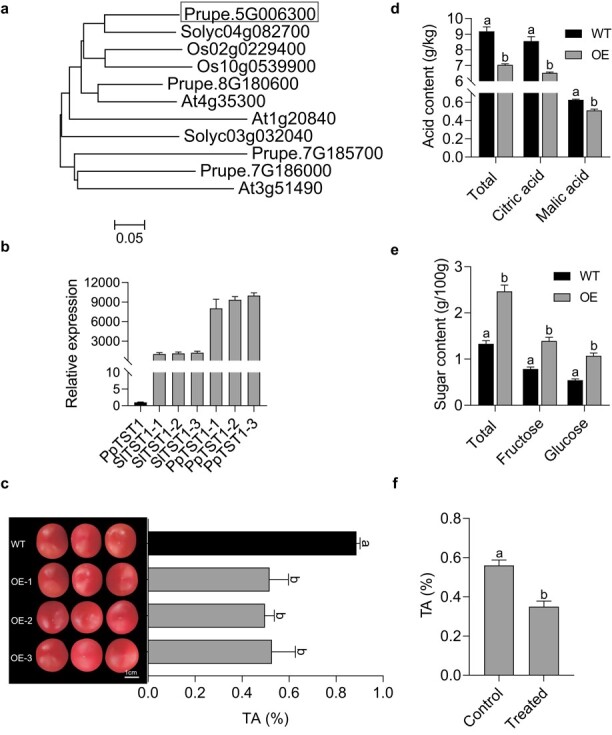
Transgenic analysis. **a** Phylogenetic analysis of PpTST1 and the other three peach TST1 members, two tomato TST1 members, three *Arabidopsis* and two rice TST1 members using amino acid sequence alignment through the neighbor-joining method with MEGA 7.0. **b** Relative expression levels of *PpTST1^His^* and *SlTST1* (*Solyc04g082700*) in ripe fruits from three transgenic tomato lines (-1, -2, -3) by qRT–PCR. *SlActin* (*Solyc10g080500*) was used as housekeeping gene. Gene expression was normalized against *SlActin* as an internal expression control. The WT was set at 1 to calculate the relative expression of transformants **c** Fruit TA values in *PpTST1^His^* transgenic tomato fruits and the WT. **d** Total and two main organic acid contents in *PpTST1^His^* transgenic tomato fruits and the WT. **e** Total and two main sugar contents in *PpTST1^His^* transgenic tomato fruits and the WT. **f** Fruit TA values in *PpTST1^His^* transgenic tomatoes treated with glucose injection and the control. Significant differences among cultivars are shown by different lowercase letters (*P* < .01, Student’s *t*-test). Error bars represents the standard error of three biological replicates.

Phylogenetic tree analysis based on the TST1 amino acid sequence revealed that PpTST1 was more closely related to an SlTST1 (Solyc04g082700) than other homologous TST1s in *Arabidopsis*, peach, tomato, and rice (Fig. 5a; Supplementary Data Fig. S4). To further confirm the role of *PpTST1* in organic acid accumulation, its two alleles were transformed into tomato. Three transgenic lines were randomly selected from each transgene for further analysis. There were no significant phenotypic differences between the WT and transgenic tomato plants (Fig. 5c). qRT–PCR analysis revealed there was no expression of *PpTST1* in the WT tomato and the expression of *PpTST1* was 8- to 12-fold that of *SlTST1* (*Solyc04g082700*) in the transgenic tomato, demonstrating the overexpression of *PpTST1* in fruits of transgenic lines (Fig. 5b; Supplementary Data Fig. S3a). The average TA value in transgenic fruits expressing *PpTST1^His^* was 0.51%, which was significantly lower than the WT (0.89%) (Fig. 5c), while TA in transgenic fruits expressing *PpTST1^Gln^* remained the same as in the WT (Supplementary Data Fig. S3b). HPLC analysis then showed that overexpression of *PpTST1^His^* decreased the contents of citric acid and malic acid by 23.7 and 18.4% (Fig. 5d). For transgenic fruits of *PpTST1^Gln^*, the contents of citric acid and malic acid remained the same as in the WT (Supplementary Data Fig. S3d). These reactions demonstrated the negative role of *PpTST1^His^* in regulating organic acid accumulation in tomato fruit.

### Two V-type proton ATPases interact with PpTST1

**Figure 6:**
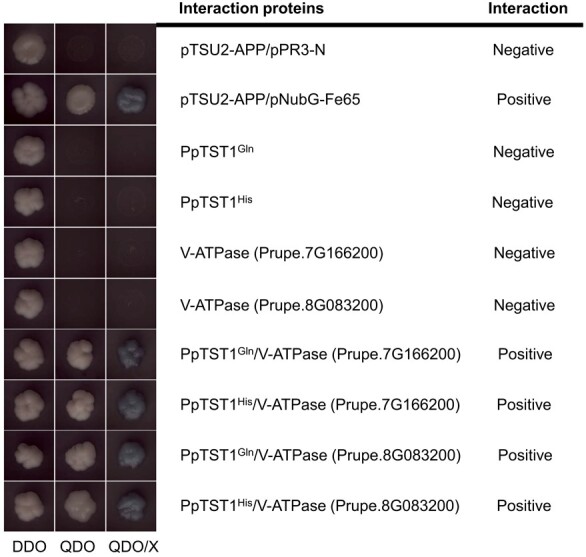
Analysis of interactions between PpTST1^Gln/His^ and two V-type proton ATPases (V-ATPases) using a Y2H system. DDO, SD/−Leu –Trp; QDO, SD/−His−Leu−Trp−Ade; QDO/X, SD/−His−Leu−Trp−Ade + X-gal.

To further explore the regulatory mechanism of organic acids in which PpTST1 participated, we utilized the DUALmembrane yeast two-hybrid (Y2N) system to screen interaction proteins of PpTST1, based on a previous report that both of the two alleles of PpTST1 were located in the tonoplast [36]. The results showed that there were 35 and 26 primary interactions with PpTST1^Gln^ and PpTST1^His^, respectively, including 11 proteins interacting with both PpTST1^Gln^ and PpTST1^His^. Supplementary Data Table S5 shows the BLAST result (nr database of GenBank) from positive prey clone sequencing, the corresponding genes, and the classification. Then we determined whether the remaining 24 PpTST1^Gln^ and 15 PpTST1^His^ primary interactions interacted with the other allele of PpTST1. From a total of 32 proteins interacting with both PpTST1^Gln^ and PpTST1^His^, 11 and 7 proteins interacting exclusively with PpTST1^Gln^ or PpTST1^His^ were identified (Supplementary Data Table S3). Among them, two proteins annotated as ‘V-type proton ATPase’ were noted, which interacted with both PpTST1^Gln^ and PpTST1^His^ (Fig. 6; Supplementary Data Table S5). The two V-type proton ATPases (V-ATPases) were members of vacuolar H^+^-pumping ATPase 16-kDa proteolipid subunit 4, a homolog of CitVHA-c4, which was reported to interact with CitERF13 and to be involved in citric acid accumulation in citrus fruit [25]. We speculated that PpTST1 might regulate organic acid accumulation by interacting with V-ATPases in peach fruit.

### Expression analysis of H(+)-ATPase, H(+)-pyrophosphatase, tonoplast dicarboxylate transporter, and aluminum-activated malate transporter/channel genes in transiently transformed peaches

**Figure 7:**
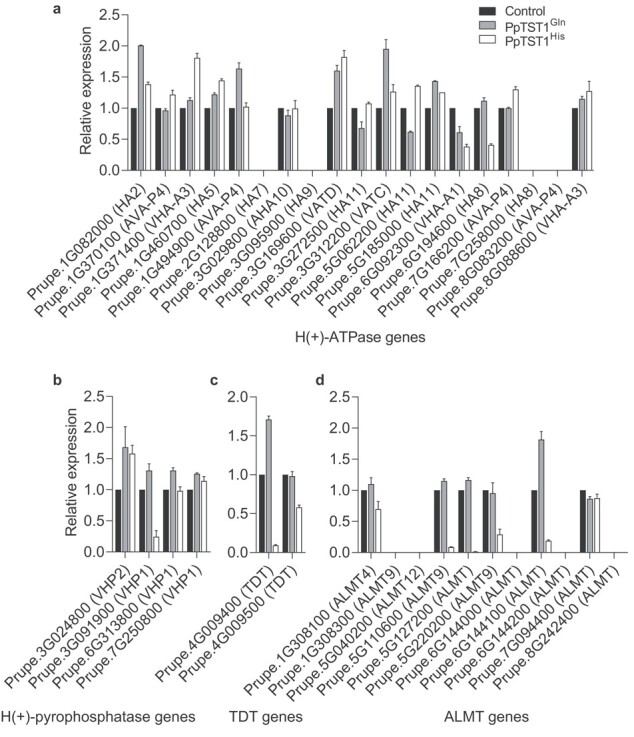
Relative expression levels of organic acid accumulation-related genes in transiently transformed peaches. **a** H(+)-ATPase genes. **b** H(+)-pyrophosphatase genes. **c** Tonoplast dicarboxylate transporter (TDT) genes. **d** Aluminum-activated malate transporter (ALMT) genes. *PpActin* (*Prupe.6G163400*) was used as housekeeping gene. Gene expression was normalized against *PpActin* as an internal expression control. The control was set at 1 to calculate the relative expression of transformants. Error bars present the standard error of three biological replicates.

The accumulation of malate and citrate in the vacuole only happened when the pH of vacuole was acidic depending on the ‘acid trap’ mechanism [12, 37]. The vacuolar H(+)-ATPase (V-ATPase) and H(+)-pyrophosphatase (V-PPase) pumped H^+^ into the vacuole to lower the vacuolar pH, thus immediately making any malate and citrate transported into the vacuole protonated [38–42]. Based on the results above, we speculated that PpTST1 might be involved in regulation of organic acid accumulation by interacting with proteins responsible for pumping H^+^. Then we quantified the expressions of genes annotated as ‘H^+^-ATPase and H^+^-pyrophosphatase in transiently transformed and control peaches, and found no significant difference (fold change ≥5) [43] (Fig. 7a and b). The malate concentration gradient across the tonoplast was maintained though this ‘acid trap’ mechanism for its facilitated diffusion [12, 17, 21, 37], which was mediated by tonoplast dicarboxylate transporter (TDT) and aluminum-activated malate transporter/channel (ALMT) [38, 39, 44, 45]. Further, we detected the expression of genes annotated as TDT and ALMT in transiently transformed and control peaches. The results revealed that one TDT gene (*Prupe.4G009400*) and three ALMT genes (*Prupe.5G110600*, *Prupe.5G127200* and *Prupe.6G144100*) had sharply decreased expression in *PpTST1^His^* transiently transformed peaches (Fig. 7c and d). The results suggested that overexpression of *PpTST1^His^* had no significant influence on proton pump genes but reduced the expression of genes involved in organic acid transport.

### Genotypes of *PpTST1* were associated with titratable acidity in diverse *Prunus* accessions

First, we used 100 accessions for investigating the relationship between TOA and TA by SPSS 17 (IBM, Armonk, NY, USA). Their TOA (ranging from 6.83 to 32.20 g/kg) and TA (ranging from 0.14 to 1.27%) values are shown in Supplementary Data Table S6. The Pearson correlation revealed a linear relationship between TOA and TA (Supplementary Data Fig. S5). Therefore we utilized TA to represent organic acid accumulation in the following analysis.

**Figure 8:**
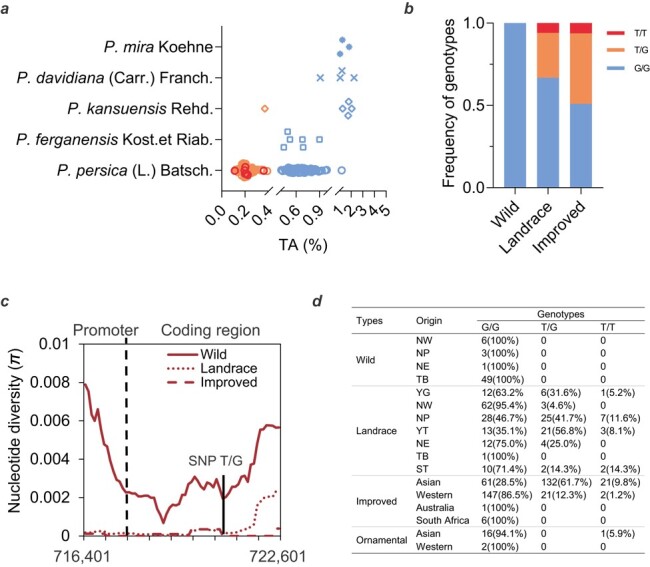
Genotype and evolution analysis. **a** Distribution of fruit TA in five *Prunus* species assessed over three consecutive years (2013–2015). The two breaks in the *X* axis are 0.4 and 1% TA values, respectively. Blue, orange, and red colors represent G/G, T/G and T/T genotypes. **b** Frequency of three genotypes in wild, landrace, and improved groups. **c** Nucleotide diversity (*π*) distribution of the wild (red solid line), landrace (red dotted line), and improved (red dashed line) covering the promoter and coding region of *PpTST1* within the 6.2-kb region. **d** Geographical distribution of 681 accessions based on genotyping with three genotypes of *PpTST1*. YG, Yun-Gui Plateau; NW, Northwest China; NP, North Plain China; YT, Yangtze River Middle and Backward; NE, Northeast China; TB, Tibet Plateau; ST, South China Subtropical [63].

To explore whether organic acid accumulation was genetically determined by the non-synonymous point mutation identified in *PpTST* in a more diverse genetic background, 169 *Prunus* germplasm accessions from five *Prunus* species were used for fruit TA measurement and marker dCAPS1584 [36] genotyping. Fruit TA value ranged widely, from 0.11 to 2.24% (Fig. 8a; Supplementary Data Table S7). However, only 11 (6.5%) accessions had TA > 1.0%, all of which are wild *Prunus* accessions except for ‘Da Guo Hei Tao’ (Fig. 8a; Supplementary Data Table S7).

Based on the marker dCAPS1584, 91 (53.8%) of the 169 accessions were genotyped G/G, 71 (42.0%) were T/G, and another 7 (4.2%) were T/T, reckoning a frequency of 0.25 and 0.75 for alleles T and G, respectively (Supplementary Data Table S7). ANOVA of TA was performed in three genotypes G/G, T/G, and T/T. The mean TA of T/T was 0.22 ± 0.07% (*n* = 7), basically identical with that of T/G (0.22 ± 0.06%; *n* = 71, *P* = 0.82); both of these were significantly lower than that of G/G (0.77 ± 0.34%; *n* = 91, *P* = 5.30686E−28 and *P* = 4.53137E−05, respectively). The result demonstrated that the genotypes of *PpTST1* had a determinative effect on TA. More importantly, all the 91 G/G accessions that span over five *Prunus* species, including 74 in *P. persica* (L.) Batsch., 6 in *P. ferganensis* Kost. et Riab., 4 in *P. davidiana* (Carr.) Franch., 4 in *P. kansuensis* Rehd and 3 in *P. mira* Koehne, had a TA level >0.4%, which could be the TA threshold value of low-acid and acidic fruit (Fig. 8a; Supplementary Data Table S7). The seven T/T accessions had TA levels that were scattered among those of the 71 T/G accessions, suggesting that there was actually little or no difference between T/G and T/T accessions and that the T genotype was dominant over G, which was consistent with low acid being dominant over acidic (Fig. 8a; Supplementary Data Table S7).

### *PpTST1* was under selection during peach domestication and improvement

The global natural population with 480 peach accessions was divided into wild, landrace, and improved groups based on the results of the neighbor-joining tree analysis and PCA using the 4 980 259 SNPs [2]. We compared the nucleotide diversity (*π*) among 480 peach accessions. The *PpTST1* region revealed decreased nucleotide diversity (*π*) in improved (5.85475e−05 and 2.38095e−05 for the promoter and coding region, respectively) and landrace (0.000123565 and 2.02966e−05) groups compared with that in the wild (0.00512883 and 0.000721035) group (Fig. 8c). The results indicated that *PpTST1* was under selection, especially during domestication. In terms of marker dCAPS1584, among the 480 accessions except 22 missing data, 100% (48/48) wild accessions had the genotype G/G, 66.8% (135/202), 27.2% (55/202), and 6.0% (12/202) landrace accessions were G/G, T/G and T/T, and 50.9% (106/208), 42.8% (89/208), and 6.3% (13/208) improved accessions were G/G, T/G and T/T. The frequency of *PpTST1^His^* increased significantly in landrace and improved accessions at a frequency of 19.6 and 27.6% compared with 0% in wild progenitors (Fig. 8b; Supplementary Data Table S8). The analysis suggested that the single-base transversion (G1584T) appeared during peach domestication, and then low-acid character selection was conducted resulting in the gradually increased frequency of *PpTST1^His^*.

In the geographical distribution of the three genotypes of *PpTST1*, we found that all the accessions originating from Southwest China, where wild peach relatives originated, had the G allele (Fig. 8d). The frequency of the T allele increased in the Asian landraces, in which it was 21.0%. For Asian improved accessions, the T allele was largely retained: 40.6%. However, for western improved accessions, the proportion of the T allele was just 7.4%.

## Discussion

### Dual function of *PpTST1* in decreasing organic acid and increasing sugar accumulation

Tonoplast sugar transporters (TSTs), previously designated as tonoplast monosaccharide transporters (TMTs), are tonoplast-localized and members of the major facilitator superfamily (MFS), possessing 12 transmembrane domains (TMs) and a unique, large central loop [46, 47]. TSTs were first reported to participate in vacuolar monosaccharide transport and play an important part during stress responses [48]. Subsequent study showed that TSTs could load high-capacity glucose and sucrose into the vacuole as proton-coupled antiporters [49]. With a highly similar amino acid sequence to *Arabidopsis* TSTs, BvTST2.1 also imports sucrose into the vacuole along with the export of protons as a proton antiporter in sugar beet [50]. In recent years, many TST family genes have been identified to play a role in sugar accumulation in fruit, such as tomato [51], sweet orange [52], pear [53–55], cucumber [56], citrus [52], apple [57, 58], grape [59, 60], watermelon [61], and melon [62].

A recent study also reported that *PpTST1* played a part in fruit sugar accumulation in peach [36]. Peng *et al*. [36] used ‘Jinxiu’, with the low-acid trait, as plant material to perform tobacco rattle virus (TRV)-mediated virus-induced gene silencing (VIGS) analysis, which revealed that the contents of total sugars, sucrose, glucose, and fructose were decreased by silencing of *PpTST1*. However, sorbitol content had no significant change. In our study, we used ‘Tianjin Shui Mi’, with the acidic trait, as plant material to conduct a transient overexpression assay, which showed that overexpression of *PpTST1* increased the content of sucrose. Moreover, overexpression of *PpTST1^His^* increased sorbitol and total sugar contents, and overexpression of *PpTST1^Gln^* reduced fructose and sorbitol contents (Fig. 4d). Peng *et al*. [36] showed only total sugars and the sucrose accumulation pattern displayed consistency with the expression profile of *PpTST1* from two accessions (XH6H and WHJ), in which sucrose was the predominant sugar in peach fruits. Overall, *PpTST1* may play a role in sucrose (the predominant sugar) accumulation, which is supported by Peng *et al*. [36] and our study. The influences of *PpTST1* on other sugars need to be further studied. We also measured 304 peach accessions (Supplementary Data Fig. S6), which confirmed the positive effect of *PpTST1^His^* on total sugar accumulation.

In the present study, we identified *PpTST1* as a key gene for organic acid accumulation in peach using GWAS and comparative transcriptome analysis (Figs 1 and 2). Overexpression of *PpTST1* in peach and tomato revealed that the allele of *PpTST1* with the single-base transversion (*PpTST1^His^*) reduced organic acid accumulation (Figs 4c and 5d; Supplementary Data Fig. S3d). Further, we treated transgenic tomato fruits expressing *PpTST1^His^* and *PpTST1^Gln^* with 25 g/L (almost 10 times of the concentration of wild type) glucose injection at the onset of ripening, resulting in the appearance of fruit dots and TA reduction by 37.5% in tomato fruits expressing *PpTST1^His^* but no significant change in the tomato fruits expressing *PpTST1^Gln^* (Fig. 5f; Supplementary Data Fig. S3c). PpTST1, annotated as tonoplast sugar/proton antiporter, might antiport sugars and protons to have a negative influence on acidifying vacuoles. The accumulation of malate and citrate in the vacuole only happened when the pH of the vacuole was acidic, depending on the ‘acid trap’ mechanism. In addition, four organic acid transporter genes had sharply decreased expression in *PpTST1^His^* transiently transformed peaches (Fig. 7c and d). Therefore we speculated that PpTST1^His^ might reduce citrate and malate accumulation through a negative influence on acidifying vacuoles and the transport of organic acids to the vacuole.

### A single-base transversion (G1584T) in *PpTST1* might be responsible for organic acid reduction

Analysis of tissue-specific expression of *PpTST1* showed that expression was relatively higher in fruit in both low-acid and acidic accessions belonging to landrace and improved cultivars (including three low-acid and three acidic accessions) (Fig. 3c). The expression profile of *PpTST1* in both ‘Hakuho’ and ‘Tianjin Shui Mi’ showed an elevated increase at stage 5 and stage 6, which was the crucial period of organic acid reduction in low-acid accessions (Fig. 3d). Meanwhile, as shown in Fig. 4a, relative expression level of *PpTST1^His^* was much higher than that of *PpTST1^Gln^*. We assumed that the difference in expression levels between the two *PpTST1* alleles might affect the results of organic acid and sugar content analyses. If this hypothesis were true, the total organic acid and malic acid contents of transgenic fruits overexpressing *PpTST1^Gln^* should be changed compared with the control, even if not as significantly as in transgenic fruits overexpressing *PpTST1^His^*. Based on the above results, we speculated that the expression level of *PpTST1* contributed to organic acid regulation in peach fruit but might not be the determining factor.

In *Arabidopsis*, Yuan *et al*. [81] found that carotenoid overaccumulation was induced by an ORANGE (OR) protein with a single amino acid substitution (R90H). However, carotenogenic gene expression was not greatly affected by either AtOR or AtOR^His^. Moreover both AtOR^His^ and AtOR posttranscriptionally regulated PSY protein abundance by interacting with phytoene synthase (PSY). Finally, the authors found that carotenoid overproduction was promoted since AtOR^His^ had the unique ability to mediate chromoplast biogenesis.

In our study, two genotypes of *PpTST1* (*PpTST1^Gln^* and *PpTST1^His^*) were associated with TA in diverse *Prunus* accessions. The non-synonymous single-base transversion (G1584T) in the third exon, which leads to a single amino acid substitution (Q528H), might be the major determinant of organic acid reduction by PpTST1. In Y2H analysis, both PpTST1^Gln^ and PpTST1^His^ interacted with two V-type proton ATPases (Fig. 6), which was responsible for pumping H+ into the vacuole, lowering the vacuolar pH [38–42]. After exploring the expression of genes annotated as H+-ATPase and H+-pyrophosphatase in transiently transformed peaches and control, we found that overexpression of *PpTST1^His^* had no significant influence on proton pump genes, suggesting PpTST1^His^ reduces citrate and malate accumulation might not through interacting with proton pump proteins. Based on the results above, we obtained two hypotheses. One was that PpTST1 acted as an antiporter that drove import of sugar into the vacuole and the export of protons from the vacuole, and the single amino acid substitution (Q528H) greatly enhanced transport activity. The other was that PpTST1^His^ gained the unique ability to stimulate the activity of organic acid transport. Apart from vacuolar pH, the other main determinant of malate and citrate accumulation in the vacuole is the inside positive ψ (the inside positive electrochemical potential gradient) [12], which may act as an abiotic stress like osmotic stress. Four proteins annotated as ‘protein phosphatase 2C’, ‘calcium-dependent lipid-binding family protein’, ‘ABC transporter’ and ‘zinc finger CCCH domain-containing protein’ only interacted with PpTST1^His^ (Supplementary Data Table S5). In a previous study, TST was reported to play an important part during stress responses [48]. Protein phosphatase 2C could respond to osmotic stress in an abscisic acid-independent manner [63]. A calcium-dependent lipid-binding protein is a regulator of osmotic stress tolerance in rice [64]. ABC transporter is required for detoxification of Al in rice and transports UDP-glucose [65]. Two CCCH-type zinc finger proteins were reported to regulate salt stress responses in *Arabidopsis* [66]. The response and regulation of Δψ could be a strong candidate activator for organic acid transport. Based on our first hypothesis, the functionality of the G allele is not completely inactive, but it is at a significantly lower level compared with the T allele and is hyperactivated by the G1528T mutations. According to our second hypothesis, PpTST1^His^ gained the unique ability to stimulate organic acid transport activity, but PpTST1^Gln^ is completely inactive in this function. The hypotheses need to be further confirmed.

### Evolutionary relationship between low-acid phenotype and *PpTST1*

According to examination of peach germplasm, all wild peach accessions are acidic. The low-acid phenotype arose in landraces and improved accessions, suggesting that the low-acid phenotype in peach may originate from natural mutation and human selection. Interestingly, *PpTST1* was under selection during domestication based on the result of nucleotide diversity analysis (Fig. 8c). The single-base transversion (G1584T) appeared during peach domestication, and then the frequency of *PpTST1^His^* gradually increased (Fig. 8b), probably due to human selection for the low-acid trait. The geographical distribution of the two alleles of *PpTST1* further supported this hypothesis (Fig. 8d). Moreover, based on the geographical distribution of these two alleles of *PpTST1* across the globe, it might be that the allele frequency of T has risen during the evolutionary transition from wild relatives to landrace, and then the allele frequency increased in Asian improved cultivars but declined in Western improved cultivars. Based on the domestication history of peach [67], we hypothesized that the T allele originated from wild relatives, but underwent different selection pressures during domestication and subsequent spread. Furthermore, in wild related species, the expression of *PpTST1* in fruit was not relatively higher than in other tissues (Fig. 3c). The function of *PpTST1* might change during peach domestication.

## Materials and methods

### Plant materials

A total of 227 peach accessions consisting of 5 wild accessions, 150 landraces, and 72 improved varieties (Supplementary Data Table S1) were used for organic acid extraction and measurement. To maintain consistency in maturity, one person collected at least nine fruits (representing three biological replicates) from each cultivar at maturity. The fruit was considered mature when the fruit skin background color changed, fruit size was not increasing, and the fruits softened and were easily picked. Fruit samples were peeled and had the pits removed. The mesocarp tissues were immediately diced, frozen in liquid nitrogen, and then stored at −80°C for analysis. Fresh leaves of the 227 accessions were sampled, frozen in liquid nitrogen, and then stored at −80°C for DNA extraction and re-sequencing.

Peach cultivars ‘Tianjin Shui Mi’ (acidic), ‘Sun Red’ (acidic), ‘Hakuho’ (low acid), and ‘Jinfeng’ (low acid) were chosen to detect dynamic changes in organic acids at six development stages of fruit because they had similar bloom and maturation stages. The genotypes of these four cultivars at the key locus (Chr05: 720760 bp) in this study were G/G, G/G, T/G, and T/T, respectively. At least nine fruits were sampled at 20, 40, 60, 80, 100, and 120 days after bloom. Every cultivar contained three biological replicates, each consisting of at least three fruits. Fruit samples were processed as previously described. We used ‘Tianjin Shui Mi’ (acidic) and ‘Hakuho’ (low acid) to screen differentially expressed genes (DEGs) that presented corresponding expression profiles with dynamic changes in organic acids at six development stages. Two biological replicates were sampled for each stage of every cultivar. Frozen mesocarp tissue was used for RNA extraction and sequencing.

‘Yangzhou 431’ (low acid), ‘Huang 07-4-28’ (acidic) and 30 *F*_1_ individuals from *P. persica* ‘Yangzhou 431’ (low-acid) × ‘Huang 07-4-28’ (acidic) were used for bulk segregant analysis sequencing (BSA-seq). The genotypes of ‘Yangzhou 431’ (low acid) and ‘Huang 07-4-28’ (acidic) at the key locus (Chr05: 720760 bp) in this study were T/G and G/G, respectively. A total of 118 *F*_1_ individuals were obtained from a cross between ‘Yangzhou 431’ (low acid) and ‘Huang 07-4-28’ (acidic). Among the 118 *F*_1_ individuals, there were 58 low-acid and 60 acidic varieties after taste evaluation. The segregation ratio was close to 1:1. We selected 15 *F*_1_ individuals with the extreme low-acid trait and 15 *F*_1_ individuals with the extreme acidic trait for bulk construction.

We used 169 *Prunus* germplasm accessions from five *Prunus* species for fruit TA measurement and marker genotyping. Fruit and leaf samples were processed as previously described.

We chose nine accessions to carry out the expression analysis of candidate genes in different tissues. These accessions could be classified into wild related species [‘Zhou Xing Shan Tao’ (acidic), ‘Hong Gen Gan Su Tao’ (acidic), and ‘A Ba Gung He Tao’ (acidic)], edible landraces [‘Nanshan Tian Tao’ (low acid), ‘Huo Lian Jin Dan’ (acidic), and ‘Chinese Cling’ (acidic)], and improved varieties [‘96-2-51’ (low acid), ‘Yu Lu’ (low acid), and ‘Fantasia’ (acidic)]. Five tissues, namely seeds, mature fruit, mature leaves, phloem, and roots, were sampled at fruit maturity for total RNA sequencing.

All the varieties used in this study were obtained from the experimental farm of the National Peach Germplasm Repository of China (NPGRC) (Zhengzhou).

### Phenotypic evaluation

The method for organic acid extraction and measurement followed the method described by Ma *et al*. [16]. For each replicate, the mesocarp of peach flesh was ground into powder in liquid nitrogen with a mortar and pestle and then 5 g of powder was dissolved in 50 mL deionized water obtained using the Arium Comfort II ultrapure water system H_2_O-II-I-UV-T (Sartorius, Goettingen, Germany). The mixture was extracted in an ultrasonic bath for 30 min, and we then transferred 1.5 mL of the mixture into a 2-mL clean centrifuge tube and centrifuged at 10 000 × g for 5 min. The supernatant was purified with a 2.5 mL syringe filter (Shuguang Hui Zhikang Biotechnology Co. Ltd, Henan, China), and the purified supernatant was filtered using a 0.22-μm organic phase needle filter (ANPEL, China) into a 2-mL clean sample bottle (Agilent, China), and then loaded into the autosampler to measure organic acid content using a Waters Alliance 2695-996 HPLC system (Waters, Milford, MA, USA). Chromatographic separation was performed with a Capcell Pak C18 (Catalog No. 90104) column (4.6 mm i.d. × 250 mm, 5 μm), while maintained the column temperature at 40°C. We applied the mobile phase of 0.1% H_3_PO_4_ solution and performed elution at the flow rate of 0.8 mL/min. Ultraviolet (UV) absorbance detection at 210 nm was utilized to quantify the acid concentration and we calculated acid concentration through comparison with the values acquired from a standard curve. The sum of major organic acids is considered as total organic acid in this paper.

Analyses of TA were performed according to Flores *et al*. [68]. TA was measured through manual titration with a 0.1 M NaOH solution up to pH 8.1 in triplicate, and expressed as percentage of fresh weight. The contents of sucrose, glucose, fructose, sorbitol, and sucrose were measured using the same method as Cao *et al*. [33].

We evaluated the taste according to the method described by Colaric *et al*. [69]. Briefly, the taste evaluation was conducted by 10 panelists. For each cultivar, 10 fruits with similar size, appearance, and ripeness were evaluated by each panelist independently. We carried out the evaluations at room temperature (24°C). The taste (a combination of sweet and sour) was evaluated on a four-step scale from sweet to sour (1 for sweet, 2 for sweet–sour, 3 for sour–sweet, and 4 for sour fruit). Sweet was grouped into low-acid, and sour, sweet–sour, and sour–sweet were grouped into acidic.

### DNA extraction and re-sequencing

A genomic DNA sequencing library was constructed using at least 4 μg of genomic DNA extracted from fresh leaves by the CTAB method [70] for each accession. The resulting libraries were sequenced on a HiSeq2500 in paired-end mode with a 49-, 90-, or 125-bp read length. More than 1 Gb of data was achieved for every accession.

### SNP calling

After trimming, the clean reads were aligned against the peach reference genome v2.0 [71] using BWA [72] (version 0.7.12). Format conversion from SAM to BAM was carried out with SAMtools v1.3.1 [73]. We used the MarkDuplicates tool (http://broadinstitute.github.io/picard/) to remove duplicate reads from the PCR. SNP calling was conducted using the Genome Analysis Toolkit v3.4 (GATK [74]). SNPs of low mapping quality (<20) were filtered out before analysis using SAMtools [73] (V1.3.1).

### Genome-wide association studies

Association analysis of total organic acid content in fruit was performed for 1 172 894 filtered SNPs from 227 accessions using the mixed linear model (MLM) in Efficient Mixed-Model Association eXpedited (EMMAX) [75] (version beta). SNPs were filtered with minor allele frequency <0.05 after genotype imputation in order to improve the statistical power of the analysis. The EMMAX emmax-kin program was utilized to calculate the pairwise relationship matrix. The resulting kinship matrix was applied in the MLM association models as a correction for population structure to calculate *P*-values to associate every SNP marker with the trait of interest [75]. The Bonferroni test threshold (set as 0.05/total SNPs) was utilized to screen significant association signals based on the *P*-value.

### RNA-seq and expression analysis

All the DNA-free RNA samples were isolated according to the modified instructions for an RNA Extraction Kit (product code RN53, Aidlab, Beijing, China), which added DNase treatments with DNase I (RNase free) (product code GT512, Huayueyang, Beijing, China). Paired-end RNA sequencing was conducted with a HiSeq 2500 system (Illumina) (125 or 150 bp paired-end reads). After removal of the adapter sequences and low-quality bases with Trimmomatic version 0.33 [76], the resulting clean reads were then aligned to the peach reference genome v2.0 [71] using TopHat v2.1.0 [77]. The number of reads per kilobase per million (RPKM) mapped reads and normalized values were calculated using Cufflinks (http://cufflinks.cbcb.umd.edu/) and RSEM [78].

### Identification of quantitative trait loci related to low-acid/acidic phenotype in peach

To identify candidate QTLs related to low-acid/acidic phenotype, BSA-seq was carried out as described by Takagi *et al*. [79]. Briefly, young healthy leaves from parents and each individual of *F*_1_ populations were sampled for genomic DNA extraction by the modified CTAB method [70]. Two DNA pools were constructed, each consisting of an equal amount of DNA from 15 low-acid (‘Yangzhou’ 431 type) or 15 acidic (‘Huang’ 07-4-28 type) individuals. Sequencing libraries from ‘Yangzhou’ 431, ‘Huang’ 07-4-28, and the HiSeq 2500 platform (Illumina) (150-bp paired-end reads) were applied to analyze the two bulks at Berry Genomics, Beijing, China. We aligned the clean reads for the low-acid bulk and the acidic bulk to the ‘Lovell’ (release version 2.0_a2.1) [80] reference genomes using the BWA program [72]. The results from BWA were fed to the Genome Analysis Toolkit (GATK) [74] for variant calling. The SNP index (defined as the ratio between the number of reads of a mutant SNP and the total number of reads corresponding to the SNP) was calculated for all SNPs in the two pools of mixed samples. A sliding window with a 1-Mb window size and 1-bp step was used to estimate the distribution of average SNP index and *∆* SNP index (difference in SNP index in the two pools) in a given genomic interval. The threshold for detecting candidate QTL regions was a 0.05 confidence level.

### Transient overexpression assays

‘Hakuho’ (low acid) and ‘Tianjin Shui Mi’ (acidic) were used as templates to amplify two variant coding sequences of *Prupe.5G006300* through PCR (Supplementary Data Table S9), respectively. The resulting products were then inserted into the pri101-AN vector under the control of the cauliflower mosaic virus (CaMV) 35S promoter. The expression constructs pri101-AN-*PpTST1^His^* and pri101-AN-*PpTST1^Gln^* as well as the empty vector (pri101-AN) as a control were introduced into *Agrobacterium tumefaciens* GV3101. Peach fruits sampled at stage 5 were utilized for a transient overexpression assay [81]. Briefly, peach flesh cubes (1 cm thick) were submerged into *Agrobacterium* suspension for infiltration, and then a vacuum (−70 kPa) was applied for 30 min. The resulting flesh cubes were cultured on Murashige and Skoog (MS) medium for 2 days, and then frozen on-site in liquid nitrogen, stored at −80°C for subsequent analysis. qRT–PCR was performed to confirm the expression of *PpTST1*. Measurements of TA and organic acid content were conducted following the methods described above.

Transient overexpression assays of *Prupe.5G006400* and *Prupe.5G009600* were conducted by the same method as that used for *Prupe.5G006300*.

### Phylogenetic analysis

Eleven amino acid sequences of TST1 from *Arabidopsis thaliana*, rice, tomato, and peach were aligned with CLUSTAL W. The phylogenetic tree was constructed making use of the obtained data matrix with MEGA software version 7 by the neighbor-joining method. The parameters were as follows: p-distance as model, bootstrap (1000 replicates), and pairwise deletion of gaps/missing data.

### Ectopic transformation in tomato

The full-length open reading frames of *PpTST1^His^* and *PpTST1^Gln^* were amplified using cDNA templates from fruit of ‘Tianjin Shui Mi’ (acidic) and ‘Hakuho’ (low acid), respectively, using the gene-specific primers listed in Supplementary Data Table S9. The resulting products were inserted into the PART-CAM vector driven by cauliflower mosaic virus 35S promoter, generating two constructs, pART-CAM-TST1*^His^* and pART-CAM-TST1*^Gln^*. The resulting constructs were then transformed into micro-Tom (*Solanum lycopersicum* L.) by *A. tumefaciens* GV3101. Transformants were screened on MS medium containing 300 mg/L timentin and 50 mg/L kanamycin. The *T*_0_ plants were obtained and grown in pots under glasshouse conditions. The seedlings were analyzed again through PCR and sequencing. Matured tomato fruits were sampled to measure gene expression and organic acid content. The expression of *PpTST1* in fruit was confirmed by qRT–PCR. Three fruits derived from each transgenic line and the control were used to measure TA. Ten tomato fruits were randomly collected from transgenic lines to compare the content of organic acids with the control. Analyses of TA were performed according to Flores *et al*. [68]. TA was measured through manual titration with a 0.1 M NaOH solution up to pH 8.1 in triplicate, and expressed as percentage of fresh weight. The methods for organic acid extraction and measurement were the same as described above. We treated transgenic tomato fruits expressing *PpTST1^His^* and *PpTST1^Gln^* with 25 g/L (10 times the content) glucose injection at the onset of ripening to investigate the change in TA. Transgenic tomato fruits expressing *PpTST1^His^* and *PpTST1^Gln^* injected with distilled water were used as the control.

### Screening of PpTST1 interaction proteins by the DUALmembrane yeast two-hybrid system

The DUALmembrane [82, 83] system makes use of the split-ubiquitin mechanism to screen cDNA libraries and detect novel interactions with target proteins. We conducted a DUALmembrane screen (Dualsystems Biotech, Zurich, Switzerland) by choosing the DUALmembrane starter kit SUC appropriate for the subcellular localization of PpTST1 and the N-terminal cleavable signal sequence. pBT3-SUC with the SUC2 ORF upstream and the Cub-LexA-VP16 ORF downstream was used as the bait vector. Full-length ORFs of *PpTST1^His^* and *PpTST1^Gln^* amplified through PCR were cloned into the bait vector, and then the bait construct was transformed into the yeast strain NMY51. To verify that our bait protein was expressed correctly in yeast, co-transformation with the control plasmids pOst1-NubI [expressing wild-type (WT) Nub] and pPR3-N (expressing the NubG–nonsense peptide fusion) was performed. We used the fruit of ‘Tianjin Shui Mi’ at stage 5 to construct the cDNA library with pPR3-N as the prey vector. The screening conditions were optimized using a pilot screen as Protocol (V.) in the instructions and the appropriate concentration of 3-AT for the library screen (40 mM). Then, we co-transformed the bait and prey in yeast NMY51, and screened transformants on SD double-dropout medium (SD/−Leu−Trp) and SD quadruple-dropout medium (SD/−His−Leu−Trp−Ade). Thereafter we retested positive clones on selected quadruple dropout medium and X-Gal (0.8 mg/mL) agar plates for a β-galactosidase assay. Based on the fact that amyloid beta A4 precursor (APP) and APP-binding family B member 1 (Fe65) interact with each other, pTSU2-APP plus pNubG-Fe65 were applied as a positive control, and pTSU2-APP plus pPR3-N were utilized as a negative control. Positive clones were confirmed by passing plasmids via *Escherichia coli* and retransforming them in the yeast two-hybrid host strain.

### Molecular diversity

In order to determine the selective sweeps in *PpTST1* related to peach domestication and improvement events, the *θπ* ratio method was used to conduct molecular diversity analysis by VCFtools v.3 [84]. Briefly, a sliding window approach with window size of 1000 bp and step size of 100 bp was used to calculate *θπ* ratios.

### Expression assays by qRT–PCR

Total RNA was extracted from frozen flesh cubes of transiently transformed peaches and the control using an extraction kit (Aidlab, Beijing, China). We used 1 μg RNA for first-strand cDNA synthesis with the Transcriptor First Strand cDNA Synthesis Kit (Takara, Dalian, China), following the manufacturer’s protocol. The sequences of the H(+)-ATPase genes, H(+)-pyrophosphatase genes, tonoplast dicarboxylate transporter (TDT) genes, and ALMT genes were obtained from the Genome Database for Rosaceae (GDR; www.rosaceae.org). We designed specific primers using Primer-BLAST software (National Center for Biotechnology Information, MD, USA). qRT–PCR was conducted on the LightCycler System (Roche LightCycler 480; Roche Diagnostics), according to the manufacturer’s protocol. We computed relative gene expression levels via the 2^−ΔΔCT^ method. Gene expression was normalized against actin as an internal expression control. The control was set at 1 to calculate the relative expression of transformants. Supplementary Data Table S9 lists the primers.

## Supplementary Material

Web_Material_uhac026

## Data Availability

The raw re-sequencing data have been deposited in the National Center for Biotechnology Information (NCBI). Accession numbers in the Sequence Read Archive are listed in Supplementary Data Table S1. The raw RNA-seq data and BSA data are available in the NCBI Sequence Read Archive under BioProject PRJNA762288 and PRJNA761716.
