# Supplementary material for: Multi-omics approaches identify a key gene, PpTST1, for organic acid accumulation in peach
Source: Hortic Res. 2022 Feb 19;9:uhac026. doi: 10.1093/hr/uhac026 (PMC9171119; doi:10.1093/hr/uhac026)
Supplement: Web_Material_uhac026 [file web_material_uhac026.zip › Figure S1-S6.docx]

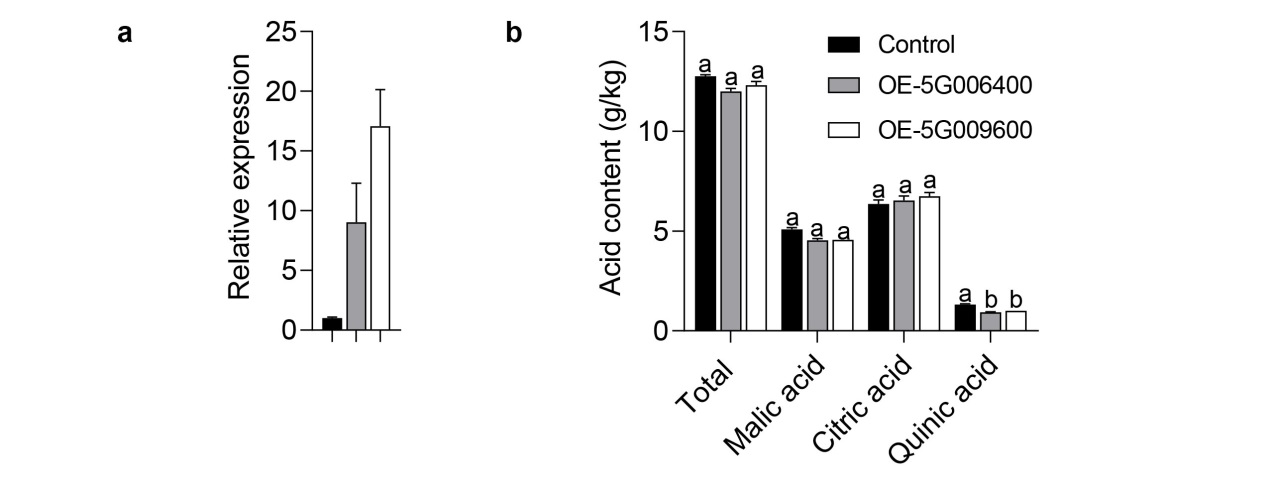


**Figure S1.** (a) Relative expression levels of *Prupe.5G006400* and *Prupe.5G009600* in fruits from transiently transformed peaches and the control by qRT-PCR. *PpActin* (*Prupe.6G163400*) was used as housekeeping gene. Gene expression was normalized against *PpActin* as an internal expression control. And then put the control at 1 to calculate the relative expression of transformants. (b) Total and three main organic acid contents in transiently transformed peaches and the control. OE-5G006400: overexpression of *Prupe.5G006400*, OE-5G009600: overexpression of *Prupe.5G009600*. Significant differences among cultivars were showed by different lowercase letters (P < 0.01, student’s t-test). Error bars present SE of three biological replicates.


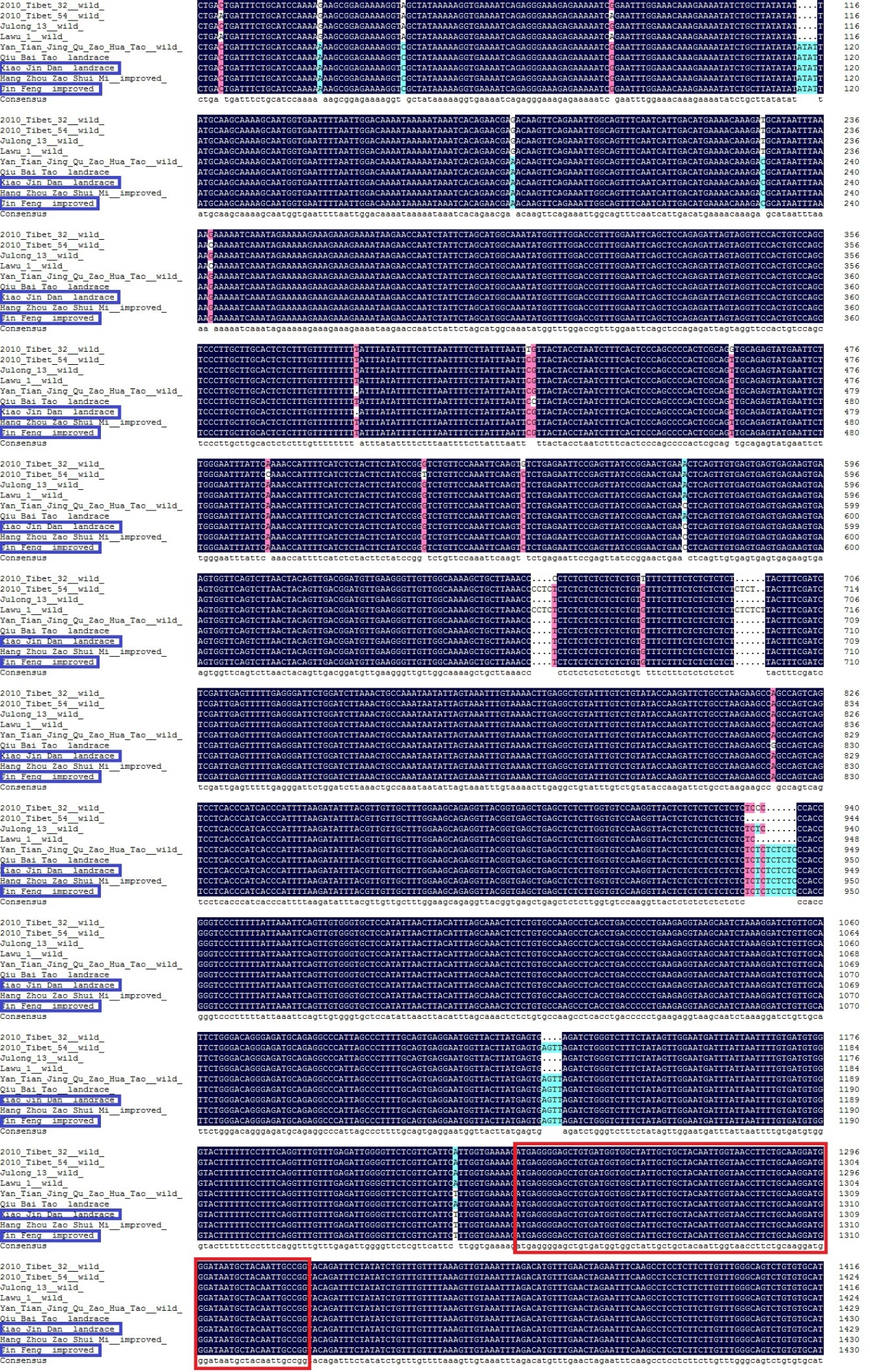


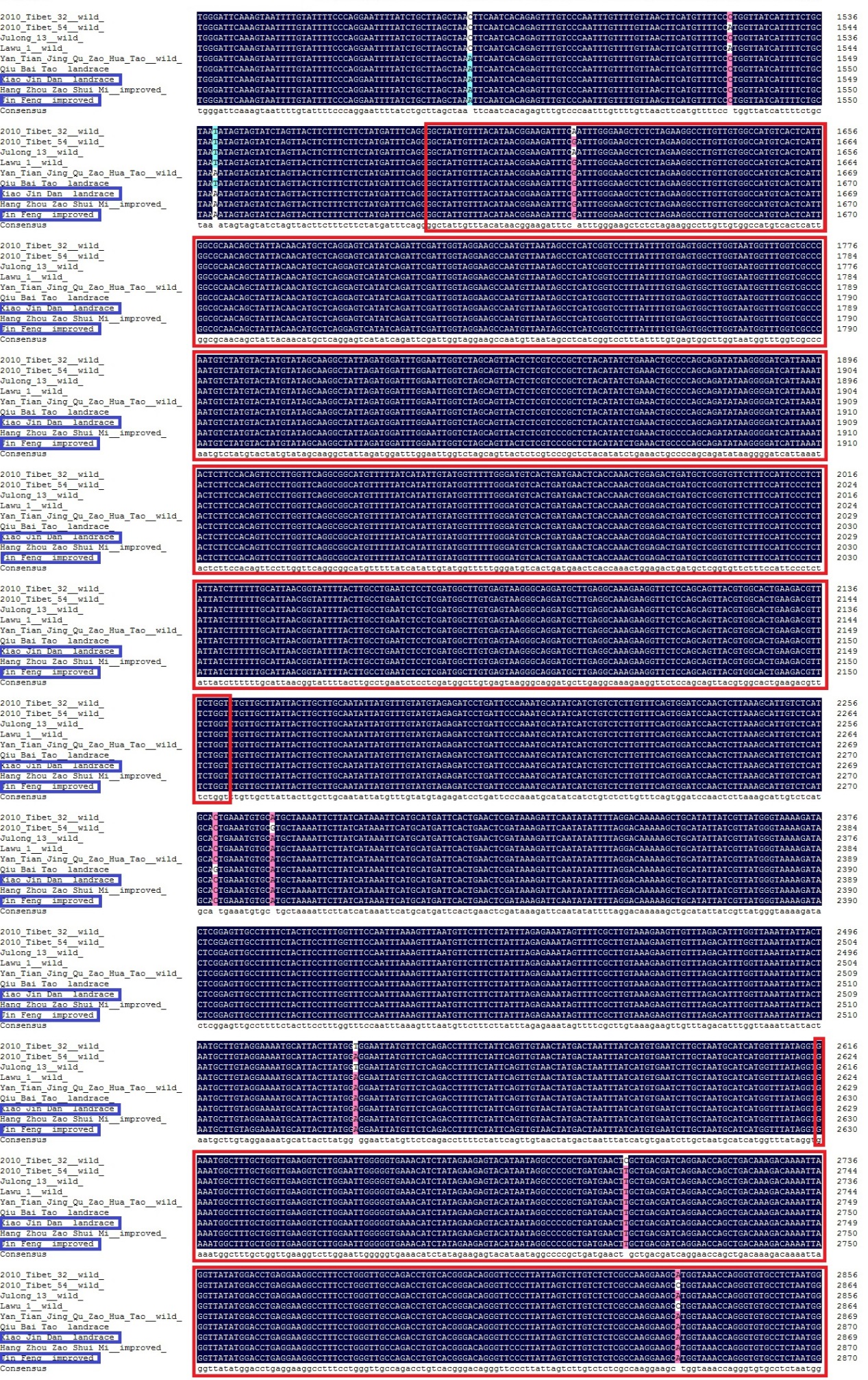


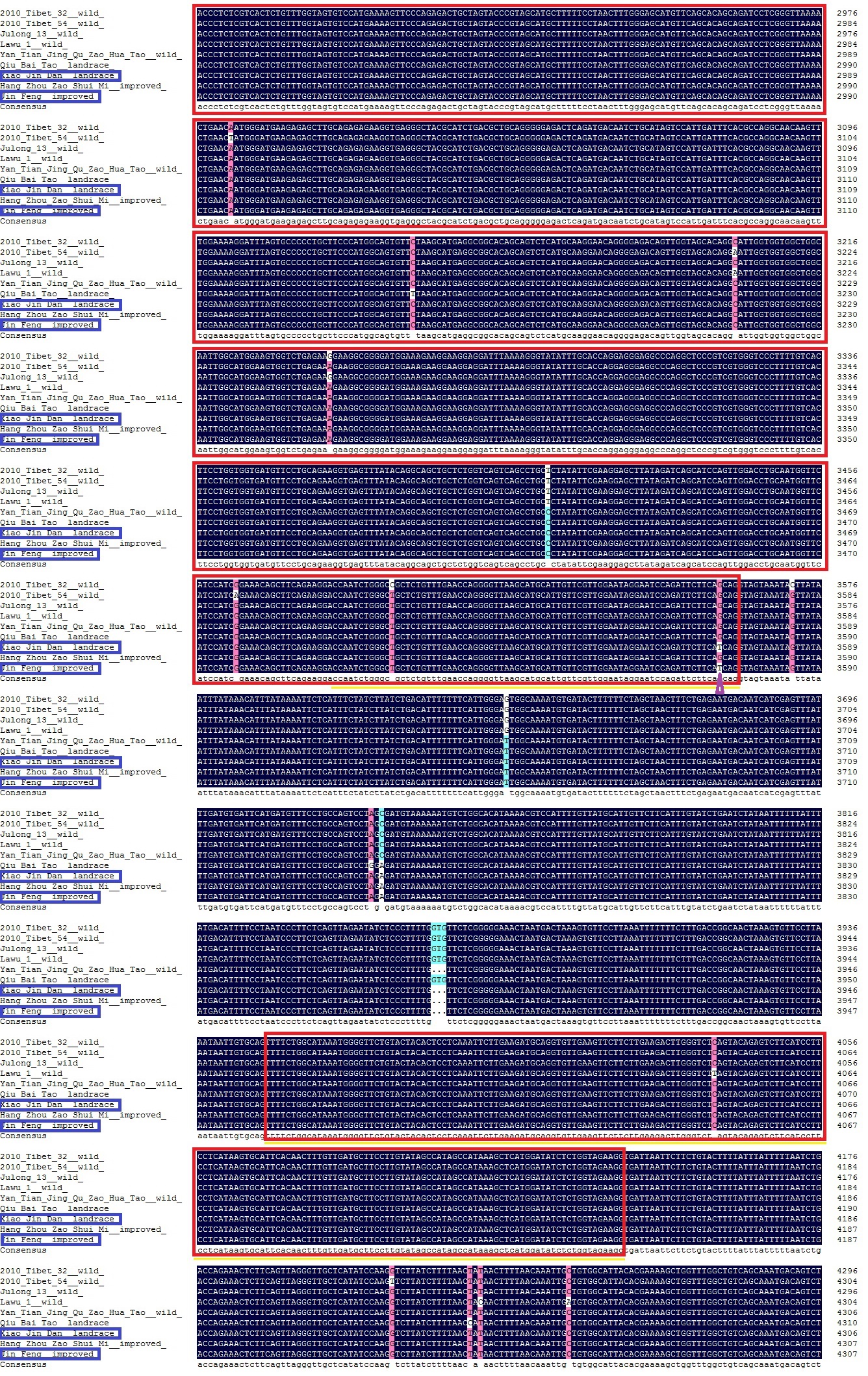


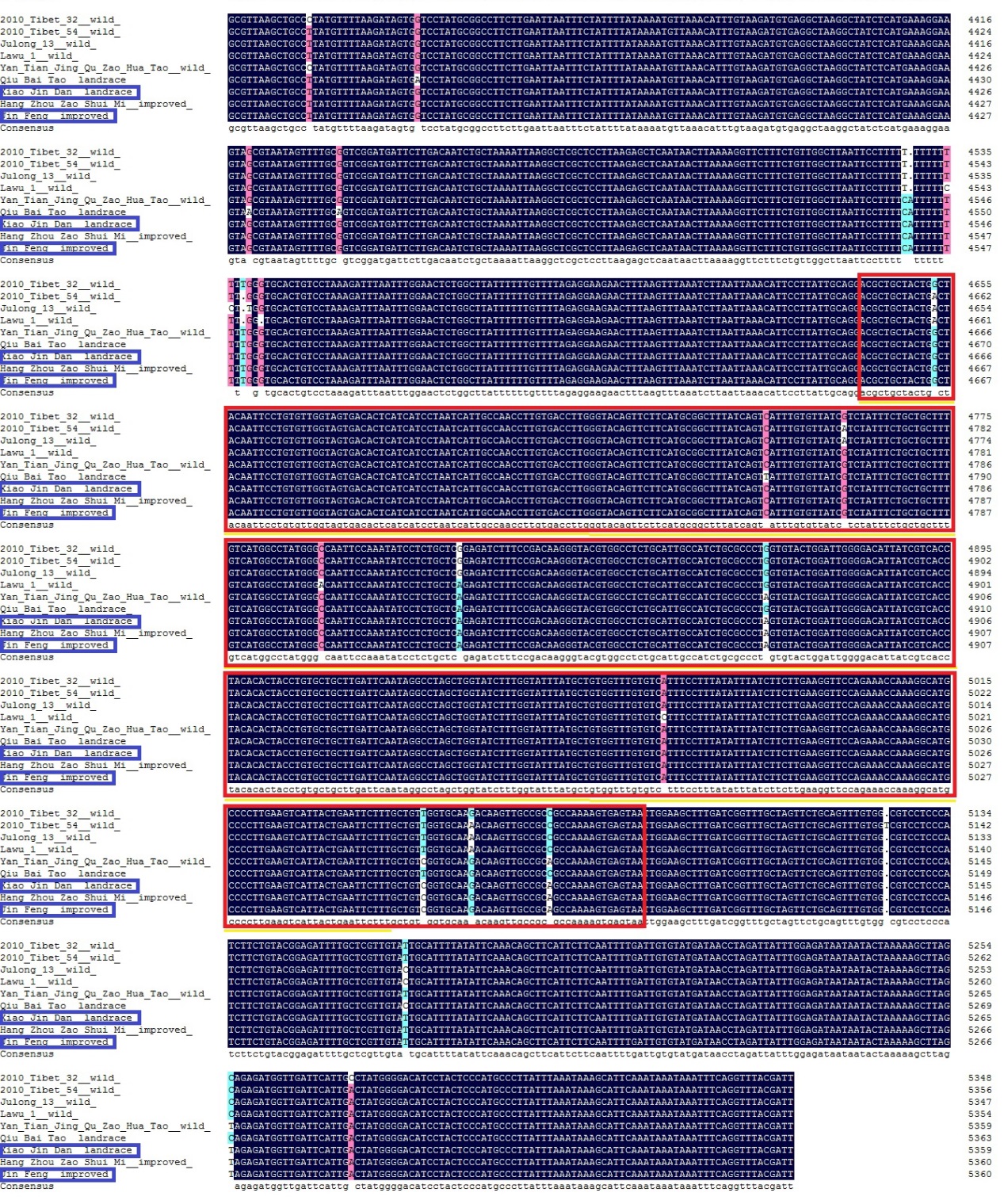


**Figure S2.** Sequence alignments of *PpTST1* from 5 wild (‘2010 Tibet 32’, ‘2010 Tibet 54’, ‘Julong 13’, ‘Lawu 1’, ‘Yan Tian Jing Qu Zao Hua Tao’), 2 landrace (‘Qiu Bai Tao’, ‘Xiao Jin Dan’) and 2 improved (‘Hang Zhou Zao Shui Mi’, ‘Jin Feng’) varieties. Among the 9 accessions, ‘Xiao Jin Dan’ and ‘Jin Feng’ are non-acid which marked by blue rectangle and the other 7 accessions are acidic. The red rectangle highlights the coding sequence. The conserved domain marked by the yellow line at the bottom of the alignment. The position marked by the purple triangle at the bottom of the alignment is relative to the locus of G/T polymorphism.


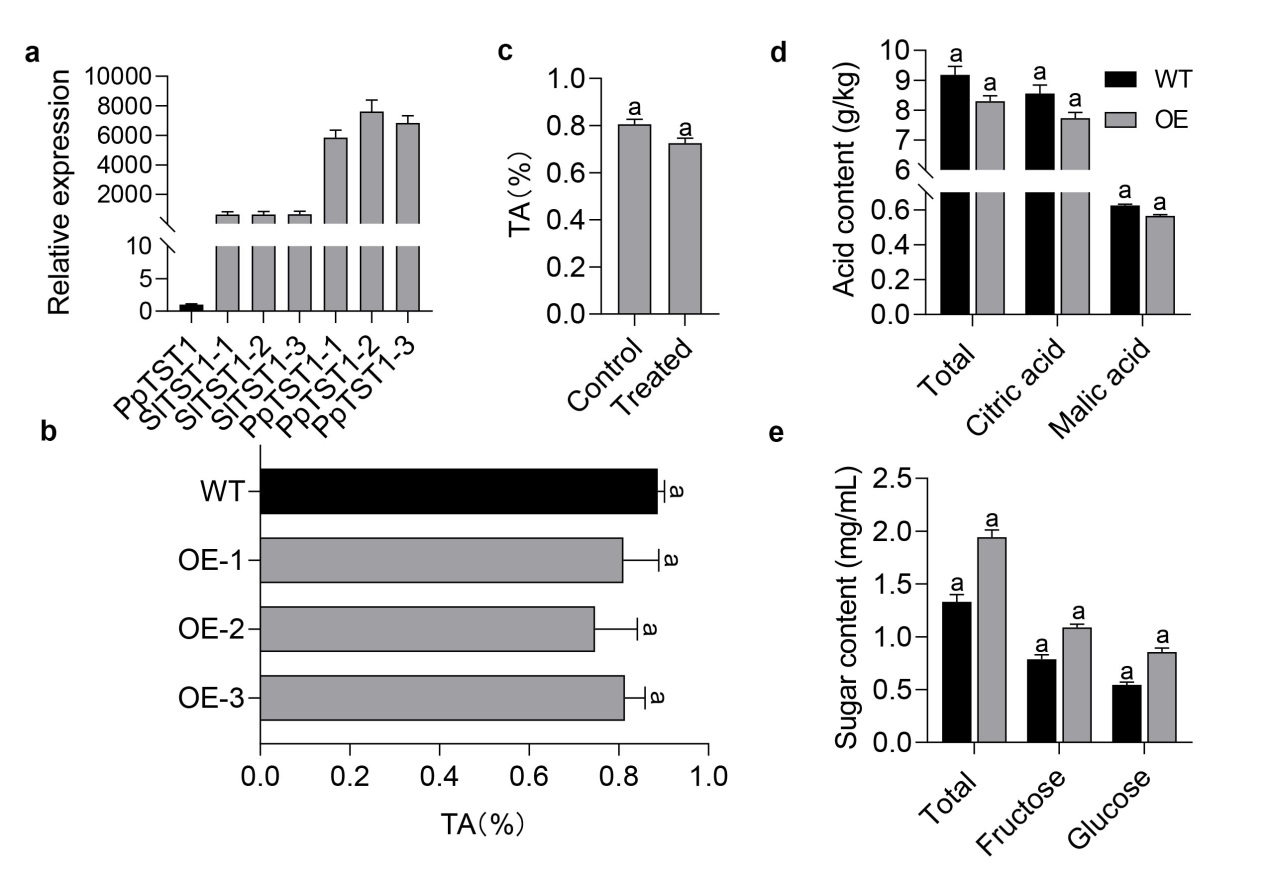


**Figure S3.** Transgenic analysis. (a) Relative expression levels of *PpTST1^Gln^* and *SlTST1 (Solyc04g082700)* in ripe fruit from three transgenic tomato lines (-1, -2, -3) from qRT-PCR. *SlActin* (*Solyc10g080500*) was used as housekeeping gene. Gene expression was normalized against *SlActin* as an internal expression control. And then put the WT at 1 to calculate the relative expression of transformants (b) Fruit TA values in *PpTST1^Gln^* transgenic tomatoes and the WT. (c) Fruit TA values in *PpTST1^Gln^* transgenic tomatoes treated with glucose injection and the control. (d) Malic acid and citric acid content in *PpTST1^Gln^* transgenic tomato fruits and the WT. (e) Fructose and glucose content in *PpTST1^Gln^* transgenic tomato fruits and the WT. Different lowercase letters indicate significant differences among cultivars (P < 0.01, student’s t-test). Error bars represent SE of three biological replicates.


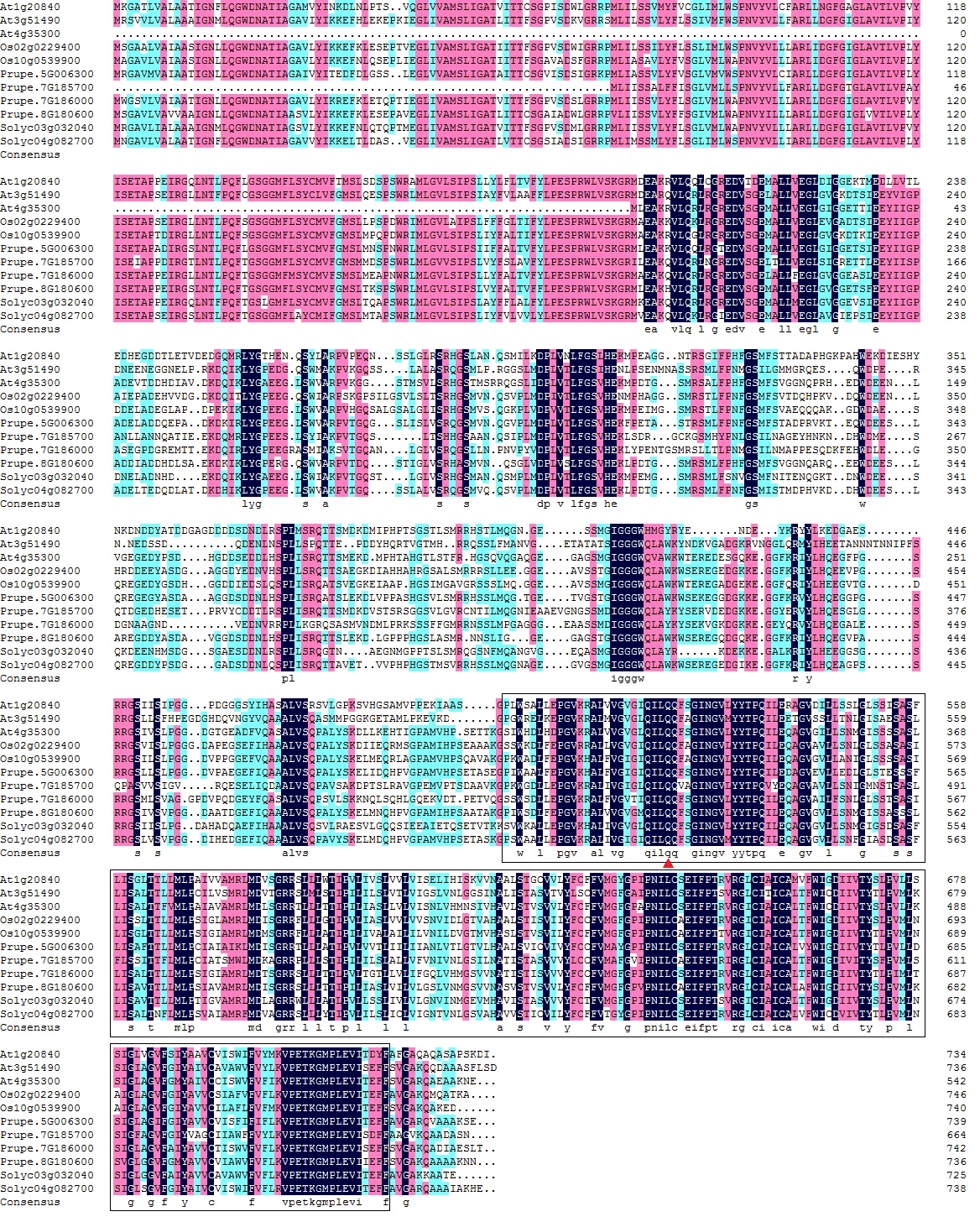


**Figure S4.** Sequence alignment of the TSTs described in Figure 5a. The residue position marked by the red triangle at the bottom of the alignment is relative to the Q528 locus. The black box highlights the conserved domain.


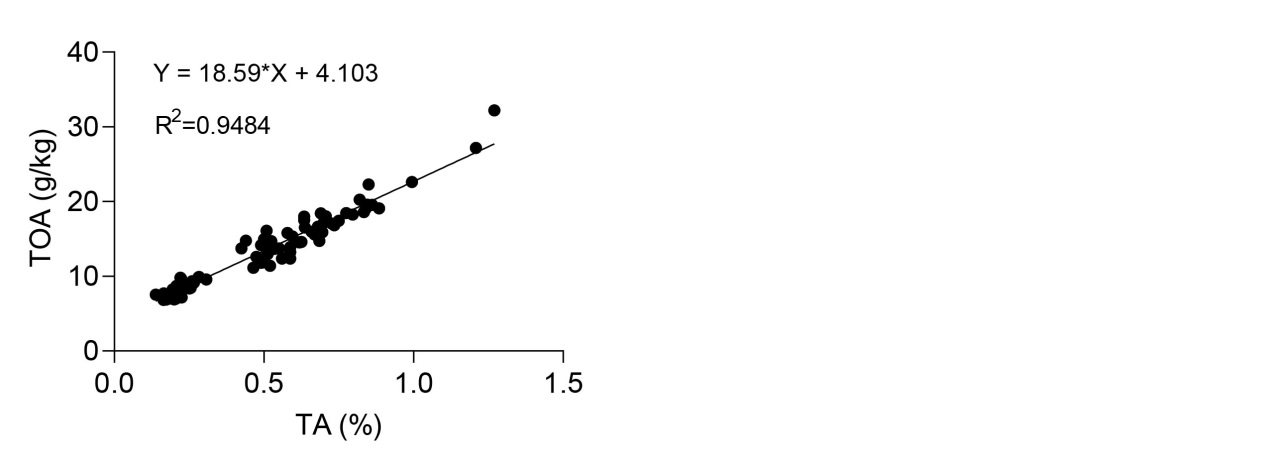


**Figure S5.** Relationship between TOA (total organic acid) and TA (titratable acidity) of 100 accessions.


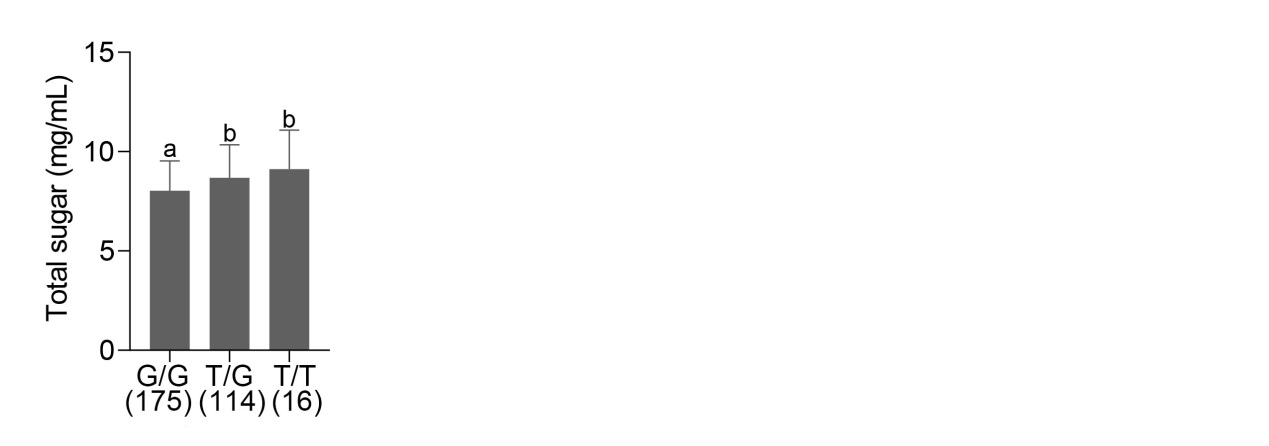


**Figure S6.** Mean values of total sugar contents in mature peach fruits of different genotypes at the G/T locus for 304 peach cultivars. Different lowercase letters indicate significant differences among cultivars (P < 0.01, student’s t-test). Error bars show the SE of the mean.
