# Supplementary material for: Multi-omics approaches identify a key gene, PpTST1, for organic acid accumulation in peach
Source: Hortic Res. 2022 Feb 19;9:uhac026. doi: 10.1093/hr/uhac026 (PMC9171119; doi:10.1093/hr/uhac026)
Supplement: Web_Material_uhac026 [file web_material_uhac026.zip › Table S1-S9.docx]

| **Table S1. Peach accessions used for measurement of organic acid and GWAS (red color)** | | | |
| --- | --- | --- | --- |
| **Accessions** | **Groups** | **Total organic acid** | **SRA** |
| Yangquan Rou Tao | Landrace | 6.52465516±1.075248782 | SRX2914899 |
| Xiang Jiao Tao | Landrace | 6.831847228±0.848945315 | SRR15831076 |
| Yu Hua Lu | Improved | 6.833750394±1.360300484 | SRX5117989 |
| Rui Guang 3 | Improved | 6.866912343±1.350779609 | SRX4994100 |
| Sunago Wase | Improved | 6.915726583±1.336764922 | SRX2914594 |
| Han Lu Mi | Landrace | 7.011257462±0.553355106 | SRX2914892 |
| Xingyi Bai Hua Tao | Landrace | 7.019552184±0.660320071 | SRX2914794 |
| Sa Hua Hong Pan Tao | Landrace | 7.089064016±0.545908956 | SRX8233325 |
| Hakuho | Improved | 7.092220629±0.545606866 | SRX4994092 |
| Shu Guang | Improved | 7.156019941±0.539501219 | SRX8233248 |
| Zhong You 5 | Improved | 7.215670578±1.250650273 | SRX1007913 |
| Shenzhou Li He Shui Mi | Landrace | 7.330971285±0.522758234 | SRX2914889 |
| Zao Huang Pan Tao | Improved | 7.379595216±1.203587111 | SRX8233282 |
| Guizhou Shui Mi | Landrace | 7.389033849±0.951166171 | SRX2914787 |
| Hong Hua Shan Tao | Wild | 7.411725176±1.194362521 | SRX2914978 |
| Hu Jing Mi Lu | Landrace | 7.434902232±1.187708333 | SRX2914942 |
| Qing Tao | Landrace | 7.436483059±0.512660672 | SRX4994095 |
| Nunome Wase | Improved | 7.51682899±1.164186961 | SRX8233295 |
| Bai Hua | Improved | 7.518314716±1.528171751 | SRX8233333 |
| Wujiang Bai | Landrace | 7.52650201±1.161409814 | SRX2914949 |
| Dong Xu Mi Tao | Improved | 7.528383912±1.160869515 | SRR11672353 |
| Yan Hong | Improved | 7.622962139±0.494814473 | SRX8233257 |
| Dawangzhuang Huang Tao | Landrace | 7.626645383±1.132658409 | SRX5117966 |
| Jin Feng | Improved | 7.653898678±0.491853821 | SRX8233193 |
| Mei Gui Hong | Improved | 7.667905185±0.490513387 | SRX8233250 |
| Xia Hui 1 | Improved | 7.703418246±1.110616733 | SRX2914593 |
| Diao Zhi Bai | Landrace | 7.880468563±0.4701709 | SRX8233284 |
| Zao Huan Gjin | Landrace | 7.887043826±1.057897383 | SRX2914991 |
| Nectagrand 4 | Improved | 7.937502064±1.043410701 | SRX5117973 |
| Qingzhou Hong Pi Mi Tao | Landrace | 7.959230135±0.687108129 | SRX2914886 |
| Rui Guang 18 | Improved | 7.991480131±1.392324811 | SRX2914739 |
| Yu Lu Pan Tao | Landrace | 7.998195659±0.519834612 | SRX2914964 |
| 8903 | Landrace | 8.013029216±1.021726672 | SRR11672164 |
| Zao Hong Zhu | Improved | 8.080597401±1.002327681 | SRX2914619 |
| Xi Nong Shui Mi | Improved | 8.094776034±0.998256962 | SRX5117988 |
| Matsumori | Improved | 8.101363688±0.996365631 | SRX2914596 |
| Zhong Nong Jin Hui | Improved | 8.143805396±1.348591858 | SRX2914735 |
| Chang Sheng Pan Tao | Landrace | 8.158713707±0.496792052 | SRX2914960 |
| Xiao Hong Tao | Landrace | 8.167228187±1.665227056 | SRX13025355 |
| Wan Pan Tao | Landrace | 8.172337916±0.838721921 | SRX2914962 |
| Hong Ya Zui | Landrace | 8.174314007±0.494552609 | SRX8233330 |
| Chen Pu Pan Tao | Landrace | 8.183093152±0.972900903 | SRX8233147 |
| Bai Mang Pan Tao | Landrace | 8.18939268±0.440606612 | SRR11672377 |
| Hua Guang | Improved | 8.225863754±0.960621359 | SRX8233258 |
| Stark Saturn | Improved | 8.229793985±0.95949298 | SRX5117972 |
| Shiwo Shui Mi | Landrace | 8.248939527±0.434907931 | SRX8233338 |
| Shen Zhou Shui Mi | Landrace | 8.257401277±0.826510973 | SRR11672177 |
| Wuhan Da Hong Pao | Landrace | 8.310898425±0.936207699 | SRX2914968 |
| Zhong Tao Hong Yu | Improved | 8.330013103±0.930719828 | SRX13025354 |
| Okubo | Improved | 8.357693921±0.4682282 | SRX4994090 |
| Feng Huang | Improved | 8.358830532±0.922446275 | SRX2914609 |
| Huang La Tao 1 | Landrace | 8.397212518±0.911426713 | SRX13025358 |
| Taiyuan Shui Mi | Landrace | 8.435711857±0.900373459 | SRX2914887 |
| Su Hong | Landrace | 8.445899449±0.897448577 | SRX2914955 |
| Kanoiwa | Improved | 8.453175284±0.895359667 | SRX2914598 |
| Longhua Shui Mi | Landrace | 8.522530172±0.444565756 | SRX2914953 |
| Hu You 003 | Improved | 8.569016467±0.862101382 | SRX2914621 |
| Mai Huang Pan Tao | Improved | 8.571284564±0.861450206 | SRX8233322 |
| Er Jie Bai | Landrace | 8.625930835±0.398829557 | SRX2914890 |
| Zhong You 5 Zao Shu Ya Bian | Improved | 8.638371605±0.842189353 | SRX13025352 |
| Shi Tou Tao | Landrace | 8.716324572±0.390178803 | SRR11672162 |
| Qing Pi Qiu Tao | Landrace | 8.720538758±0.818598965 | SRS10963131 |
| Jiaqing Pan Tao | Landrace | 8.726834028±0.576917659 | SRR11672371 |
| Xiao Hong Hua | Landrace | 8.74706284±0.810983836 | SRX2914950 |
| Zhong You Pan 4 | Improved | 8.799934265±0.795804322 | SRX1007923 |
| Wu Yue Bai | Landrace | 8.87519933±0.774195538 | SRX2914901 |
| Ji Zhui Bai | Landrace | 8.93878769±0.368888903 | SRX2914894 |
| Ying Ge Tao | Landrace | 8.952227879±0.752080455 | SRX4994089 |
| Ju Shan Tong Tao | Landrace | 9.016261986±0.733696108 | SRX2914786 |
| Shangshan Da Yu Lu | Landrace | 9.125976673±0.702196754 | SRX2914952 |
| Yu Lu | Improved | 9.213883058±0.676958618 | SRX2914963 |
| Li He Pan Tao | Landrace | 9.2800117±1.02238427 | SRX2914958 |
| Kawanakajima Hakuto | Improved | 9.344927749±0.639335369 | SRX8233373 |
| Hardired | Improved | 9.355814831±0.636209661 | SRX2914633 |
| Lulin Shui Mi | Landrace | 9.365814518±0.323511265 | SRX2914954 |
| Fei Cheng Bai Li 17 | Landrace | 9.479546324±0.965097394 | SRX2914891 |
| Ping Bei Zi | Landrace | 9.502106698±0.647831998 | SRX8233352 |
| Fei Cheng Hong Li 6 | Landrace | 9.552579565±0.944129374 | SRX2914906 |
| Han Shan Mi | Improved | 9.576580599±0.572827273 | SRX13025360 |
| Yi Xian Hong | Landrace | 9.580786785±0.571619667 | SRX2914930 |
| Wei Jian Hong Rou | Landrace | 9.616180203±0.561458131 | SRX2914969 |
| Da Zhao Huang Tao | Landrace | 9.628749181±0.30285903 | SRX13025359 |
| Changling Zao Yu Lu | Landrace | 9.654479327±0.300396634 | SRX2914945 |
| Zhao Shu Huang Gan | Landrace | 9.700578822±0.275455442 | SRX4994085 |
| Qiu Ban Jin | Landrace | 9.711585641±0.534066997 | SRS10963132 |
| Xia Ye Tao | Improved | 9.719713631±0.531733432 | SRX5118036 |
| Qin Guang | Improved | 9.736503883±0.52691291 | SRX2914620 |
| Yumyeong | Improved | 9.786157369±0.512657274 | SRX2914599 |
| Huo Lian Jin Dan | Landrace | 9.814067708±0.259163975 | SRX4994096 |
| Xiao Bai Tao | Landrace | 9.868764068±0.279889411 | SRX2914898 |
| Xingyi Wu YueTao | Landrace | 9.887927314±0.483438875 | SRX2914795 |
| Sha Hong Tao | Improved | 9.888974949±0.483138097 | SRX2914595 |
| Yang Tao | Landrace | 9.904327459±0.478730354 | SRX4994089 |
| Jing Yu | Improved | 9.912990032±0.244963555 | SRX4994098 |
| Baisha | Landrace | 9.93805057±0.273258637 | SRX2914836 |
| Long 1-2-4 | Landrace | 10.05277083±0.800523245 | SRX2914811 |
| Xi Jiao 2 | Landrace | 10.08782002±0.790460543 | SRX2914824 |
| Jingmen Tao (Mao) | Landrace | 10.2112564±0.390610311 | SRX2914967 |
| Zhang Bai 5 | Landrace | 10.34685973±0.526566673 | SRX2914846 |
| Nanshan 1 | Landrace | 10.35783356±0.233085065 | SRX2914820 |
| Da Jin Dan | Landrace | 10.42392588±0.17161809 | SRX2914798 |
| Yu Bai | Improved | 10.42423664±0.226730235 | SRX8233343 |
| Zhong You Pan 1 | Improved | 10.42673063±1.016518416 | SRX2914616 |
| Da Xue Tao | Landrace | 10.44538535±0.68780266 | SRX2914902 |
| Jian Zui Hong Rou | Landrace | 10.70327142±0.249351604 | SRX2914966 |
| Wuhan 2 | Landrace | 10.79696195±0.222452824 | SRX8233272 |
| Fenghua Pan Tao | Landrace | 10.84080678±0.111774335 | SRX8233148 |
| Dicon | Improved | 10.85752659±0.205064566 | SRX2914613 |
| Liu Yue Kong | Landrace | 10.92231709±0.186463061 | SRX2914897 |
| Jiangcun 4 | Landrace | 11.09260897±0.137571846 | SRX2914817 |
| Maria Serena | Improved | 11.14287091±0.123141522 | SRX4994099 |
| Zhang Bai Gan | Landrace | 11.14590901±0.15766561 | SRX2914847 |
| Huo Zhu | Landrace | 11.27779242±0.084405229 | SRX2914941 |
| Wu Bao Tao | Landrace | 11.36654947±0.058922869 | SRX2914922 |
| Kashi 3 | Landrace | 11.41211591±0.410251977 | SRX2914838 |
| Hei Bu Dai | Landrace | 11.45749858±0.184927448 | SRX5118039 |
| Shuang Xi Hong | Improved | 11.49811445±0.021150236 | SRX8233255 |
| Lin Bai 10 | Landrace | 11.50481168±0.38363879 | SRX2914840 |
| Fen Shou Xing | Landrace | 11.71256632±0.040419412 | SRX8233265 |
| Rou Pan Tao | Landrace | 11.7975275±0.622959307 | SRX2914856 |
| Gaotai 1 | Landrace | 11.84784051±0.31109906 | SRX2914835 |
| NJC108 | Improved | 12.36806502±0.228614679 | SRX2914611 |
| Huang La Tao 2 | Landrace | 12.36899172±0.135530605 | SRX13025353 |
| Fei Jin Tao | Landrace | 12.40425323±0.239004404 | SRX2914800 |
| Er Zao Tao | Landrace | 12.53860685±0.211938711 | SRX2914790 |
| Yangzhou 3 | Improved | 12.57930357±0.289261778 | SRX5117993 |
| Shui Bai Tao | Landrace | 12.58433026±0.290704955 | SRX2914947 |
| Zhang Huang 9 | Landrace | 12.59503691±0.070632479 | SRX13025357 |
| Da Tian Tao | Landrace | 12.81408936±0.356669356 | SRX2914946 |
| Tian Li Guang | Landrace | 12.95184162±0.396218361 | SRX2914830 |
| Dan Ban Zi Tao | Landrace | 12.99882266±0.278064552 | SRX13025362 |
| Mang Xia Lu | Improved | 13.18736572±0.118808591 | SRS10963124 |
| Huang Nian Hu | Landrace | 13.23871962±0.042618066 | SRX2914805 |
| Bai Nian He | Landrace | 13.42507657±0.097520759 | SRX2914785 |
| Huang Li Guang | Landrace | 13.48134132±0.183827667 | SRX2914828 |
| Cullinan | Improved | 13.62414418±0.28777713 | SRX2914603 |
| Chinese Cling | Landrace | 13.65773901±0.051285932 | SRX4994080 |
| Babygold 5 | Improved | 13.7277618±0.302651567 | SRX2914614 |
| Lin Huang 1 | Landrace | 13.7407733±0.622722567 | SRX2914818 |
| Xiamiao 1 | Landrace | 13.79318634±0.637770478 | SRX2914810 |
| NJN76 | Improved | 13.8837648±0.663775773 | SRX8233389 |
| Da Hong Pao | Landrace | 13.955948±0.33540797 | SRX8233355 |
| Shi Yu Bai Tao | Improved | 14.1489874±0.052150539 | SRX13025365 |
| Qinling Dong Tao | Landrace | 14.16051993±0.203094552 | SRX2914881 |
| Ren Mian Tao | Landrace | 14.23544539±0.03164452 | SRS10963129 |
| Xian Tao | Landrace | 14.45041144±0.062503155 | SRX2915040 |
| Texstar | Improved | 14.51620095±0.845349728 | SRX5117969 |
| Wanzhou Suan Tao | Landrace | 14.59988984±0.869377011 | SRX2914974 |
| Huang Jin Pan Tao | Landrace | 14.66782566±0.888881548 | SRX8233287 |
| Hong Li Guang | Landrace | 14.69089988±0.097025565 | SRX2914831 |
| Xiang Tao | Landrace | 14.71643151±0.902836406 | SRX2914976 |
| Dunhuang Dong Tao | Landrace | 14.74389582±0.222950188 | SRX2914834 |
| Kashi 1 | Landrace | 14.75945743±0.227417965 | SRX2914855 |
| Bai Nian Hu | Landrace | 14.818692±0.193822695 | SRX2914799 |
| Fuzalode | Improved | 14.96107276±0.973073499 | SRX2914632 |
| Cheonhong | Improved | 15.07290263±1.005180128 | SRX2914624 |
| Favolate 2 | Improved | 15.14917074±1.027076885 | SRX2914601 |
| Zhang Bai 2 | Landrace | 15.16877434±0.50951066 | SRX2914845 |
| Lian Huang | Improved | 15.33008314±1.079017272 | SRX2914610 |
| Ying Chun | Landrace | 15.36825389±1.08997619 | SRX2914610 |
| NJN80 | Improved | 15.48843497±1.124480469 | SRX2914625 |
| May Fire | Improved | 15.57838448±0.266525884 | SRX2914574 |
| Regina | Improved | 15.68825159±1.181848311 | SRX2914604 |
| Linze Zi Tao | Landrace | 15.7615142±1.202882185 | SRX2914842 |
| Kashi Huang Rou Li Guang | Landrace | 15.76664034±0.284542121 | SRX8233324 |
| Lincheng Tao | Landrace | 15.7834874±0.2861544 | SRX2914895 |
| Maravilha | Improved | 15.87797415±1.236318118 | SRX2914600 |
| Armking | Improved | 15.89594646±1.241478011 | SRX2914629 |
| Tian Ren Tao | Landrace | 15.90810769±0.557198242 | SRX2914854 |
| Wu Da Tao | Landrace | 15.97010921±0.898358971 | SRX2914792 |
| Bai He Tao | Landrace | 16.03811494±0.310522462 | SRX2914783 |
| Xinjiang You Pan Tao | Landrace | 16.05127381±1.286072866 | SRX2914634 |
| Li He Tian Ren | Landrace | 16.08187179±0.29670127 | SRX8233362 |
| Ping Ding Qiu Tao | Landrace | 16.19531365±1.327427059 | SRX2914880 |
| Wu Yue Xian Bian Gan | Landrace | 16.23941209±0.97567648 | SRR11672174 |
| Lin Bai 3 | Landrace | 16.35688698±1.373815152 | SRX2914841 |
| Da Guo Hei Tao | Landrace | 16.45586181±0.694273635 | SRX2914932 |
| Suan Tao | Landrace | 16.52607598±0.360467318 | SRX2914974 |
| Fei Cheng Bai Li 10 | Landrace | 16.60355994±0.715475874 | SRX2914909 |
| Kashi 4 | Landrace | 16.60366081±1.080253174 | SRX2914839 |
| Kashi 2 | Landrace | 16.62757185±1.087118092 | SRX2914837 |
| Snow Queen | Improved | 16.83365475±1.510696338 | SRX8233091 |
| Hong Shan Hu | Improved | 16.94952972±1.543964319 | SRX5118034 |
| Suan Li Guang | Landrace | 16.9933412±1.556542702 | SRX2914829 |
| Mi Yang Shan | Landrace | 17.02503089±1.201229542 | SRX2914843 |
| Bai Li Hu | Landrace | 17.07152378±0.620972686 | SRX2914784 |
| Flordagold | Improved | 17.19989793±1.615845641 | SRX2914607 |
| Qing Si Tao | Landrace | 17.21110915±1.619064412 | SRR11672234 |
| Yexian Dong Tao | Landrace | 17.24968493±0.942368328 | SRX2914900 |
| Hetian Huang Rou | Landrace | 17.32881231±0.965085986 | SRX2914776 |
| Zao Chun Tao | Landrace | 17.39643383±0.440514681 | SRR15831077 |
| Shi Tao | Landrace | 17.41450286±0.67020775 | SRX2915038 |
| Qiu Bai Tao | Landrace | 17.44898624±0.999588215 | SRX2914789 |
| Ping Yong Tao | Landrace | 17.51611703±0.846474554 | SRX2914788 |
| Tie 4-1 | Landrace | 18.00098491±0.572192285 | SRX2914821 |
| Anlong Bai Tao | Landrace | 18.01904864±0.756991033 | SRX2914782 |
| Jiang Tao | Landrace | 18.2398807±0.788691736 | SRR15831075 |
| Early Red 2 | Improved | 18.24979295±1.91727305 | SRX2914630 |
| Xinjiang Huang Rou | Landrace | 18.43374054±0.634314883 | SRX2914808 |
| NJC77 | Improved | 18.45164261±1.97522458 | SRX8233366 |
| Long You Pan Tao | Improved | 18.57526318±2.010716341 | SRX8233150 |
| Hong Tao | Landrace | 19.07528438±0.601182035 | SRX2914893 |
| Nectaross | Improved | 19.09888363±2.161049046 | SRX2914631 |
| Tian Jin Shui Mi | Landrace | 19.11018362±0.604521919 | SRX4994082 |
| Tu- 2 | Landrace | 19.1325253±0.734626281 | SRX2914823 |
| Hong Hua Bi Tao | Landrace | 19.34079766±1.542731874 | SRX2914920 |
| Croce del Sud | Improved | 19.56149446±2.29386574 | SRX2914626 |
| Yuan Chun Bai | Landrace | 19.57865799±0.980874846 | SRX2914934 |
| Sa Hong Long Zhu | Landrace | 19.69248261±2.331472755 | SRR11672259 |
| Guizhou Huang Jin Mi | Landrace | 20.01236367±2.423311387 | SRX5117959 |
| Okitsu | Improved | 20.25275658±1.239322488 | SRX4994091 |
| Nan Fang Hong Hua Zao | Landrace | 21.20068537±2.764481429 | SRX2915044 |
| Yuan Yang Chui Zhi | Landrace | 21.28099452±0.81227028 | SRX2914927 |
| Fen Hong Bi Tao | Landrace | 21.41300228±2.825438128 | SRR15831079 |
| Bai Dan Ban | Landrace | 22.04668291±2.319598098 | SRR15831078 |
| Moyu 8 | Landrace | 22.29187513±1.188154791 | SRX2914819 |
| Zhu Fen Chui Zhi | Landrace | 22.50455913±3.138826757 | SRX8233267 |
| Jiu Yang Qing Tao | Landrace | 22.60641646±1.415513253 | SRX2914793 |
| Hong Ye Tao | Landrace | 22.7101332±1.248196239 | SRX2914921 |
| Gan Su Tao | Wild | 23.9495813±1.770006166 | SRX8233217 |
| Wu Hei Ji Rou Tao | Landrace | 24.20588864±1.806799396 | SRX8233356 |
| Ju Hua Tao | Landrace | 25.66648966±1.854790471 | SRX2914917 |
| Redhaven | Improved | 26.66555808±4.333459658 | SRX4994101 |
| Kawanakajima | Improved | 26.91216729±4.404261758 | SRX8233373 |
| Zhou Xing Shan Tao | Wild | 27.17464881±4.479620836 | SRX2914915 |
| Bai Gen Gan Su Tao | Wild | 32.19725121±2.953969189 | SRR11672433 |
| Shan Gan Shan Tao | Wild | 32.62392443±5.679709758 | SRX2914851 |

| **Table S2. The 5 SNPs exceeding the significant threshold** | | | |  |  |
| --- | --- | --- | --- | --- | --- |
| Chromosome | Position | | -log10(p) |  |  |
| 5 | 996899 | | 8.177178355 |  |  |
| 5 | 995806 | | 8.055024092 |  |  |
| 5 | 761749 | | 7.89279003 |  |  |
| 5 | 812000 | | 7.754487332 |  |  |
| 5 | 1479671 | | 7.742321425 |  |  |
| 5 | 986481 | | 7.661543506 |  |  |
| 5 | 990883 | | 7.621602099 |  |  |
| 5 | 986552 | | 7.539102157 |  |  |
| 5 | 990685 | | 7.524328812 |  |  |
| 5 | 630991 | | 7.37675071 |  |  |
| 5 | 630997 | | 7.37675071 |  |  |
| 5 | 661855 | | 7.364516253 |  |  |
| 5 | 641763 | | 7.30980392 |  |  |
| 5 | 1109840 | | 7.30980392 |  |  |
| 5 | 817702 | | 7.27083521 |  |  |
| \| **Table S3. All genes may related to fruit acidity accumulation identified by two analytical strategies** \| \| \| --- \| --- \| \|  \|  \| \| **Genes identified by GWAS** \| **Genes identified by comparative transcriptome analysis among stages** \| \| Prupe.5G005400 \| Prupe.1G006300 \| \| Prupe.5G005500 \| Prupe.1G011400 \| \| Prupe.5G005600 \| Prupe.1G011500 \| \| Prupe.5G005700 \| Prupe.1G012800 \| \| Prupe.5G005800 \| Prupe.1G015600 \| \| Prupe.5G005900 \| Prupe.1G018600 \| \| Prupe.5G006000 \| Prupe.1G018700 \| \| Prupe.5G006100 \| Prupe.1G018800 \| \| Prupe.5G006200 \| Prupe.1G020300 \| \| Prupe.5G006300 \| Prupe.1G020600 \| \| Prupe.5G006400 \| Prupe.1G021800 \| \| Prupe.5G006500 \| Prupe.1G022100 \| \| Prupe.5G006600 \| Prupe.1G026400 \| \| Prupe.5G006700 \| Prupe.1G027000 \| \| Prupe.5G006800 \| Prupe.1G027300 \| \| Prupe.5G006900 \| Prupe.1G027800 \| \| Prupe.5G007000 \| Prupe.1G027900 \| \| Prupe.5G007100 \| Prupe.1G028800 \| \| Prupe.5G007200 \| Prupe.1G037600 \| \| Prupe.5G007300 \| Prupe.1G041100 \| \| Prupe.5G007400 \| Prupe.1G041800 \| \| Prupe.5G007500 \| Prupe.1G042200 \| \| Prupe.5G007600 \| Prupe.1G043700 \| \| Prupe.5G007700 \| Prupe.1G046500 \| \| Prupe.5G007800 \| Prupe.1G047900 \| \| Prupe.5G007900 \| Prupe.1G048000 \| \| Prupe.5G008000 \| Prupe.1G049000 \| \| Prupe.5G008100 \| Prupe.1G049600 \| \| Prupe.5G008200 \| Prupe.1G050200 \| \| Prupe.5G008300 \| Prupe.1G050600 \| \| Prupe.5G008400 \| Prupe.1G052400 \| \| Prupe.5G008500 \| Prupe.1G054400 \| \| Prupe.5G008600 \| Prupe.1G056800 \| \| Prupe.5G008700 \| Prupe.1G056900 \| \| Prupe.5G008800 \| Prupe.1G060900 \| \| Prupe.5G008900 \| Prupe.1G061000 \| \| Prupe.5G009000 \| Prupe.1G061100 \| \| Prupe.5G009100 \| Prupe.1G061200 \| \| Prupe.5G009200 \| Prupe.1G065300 \| \| Prupe.5G009300 \| Prupe.1G067500 \| \| Prupe.5G009400 \| Prupe.1G067600 \| \| Prupe.5G009500 \| Prupe.1G069600 \| \| Prupe.5G009600 \| Prupe.1G070500 \| \| Prupe.5G009700 \| Prupe.1G073400 \| \| Prupe.5G009800 \| Prupe.1G074600 \| \| Prupe.5G009900 \| Prupe.1G076400 \| \| Prupe.5G010000 \| Prupe.1G076900 \| \| Prupe.5G010100 \| Prupe.1G087900 \| \| Prupe.5G010200 \| Prupe.1G091700 \| \| Prupe.5G010300 \| Prupe.1G092500 \| \| Prupe.5G010400 \| Prupe.1G097600 \| \| Prupe.5G010500 \| Prupe.1G099600 \| \| Prupe.5G010600 \| Prupe.1G101900 \| \| Prupe.5G010700 \| Prupe.1G102500 \| \| Prupe.5G010800 \| Prupe.1G103900 \| \| Prupe.5G010900 \| Prupe.1G106900 \| \| Prupe.5G011000 \| Prupe.1G107000 \| \| Prupe.5G011100 \| Prupe.1G109500 \| \| Prupe.5G011200 \| Prupe.1G110600 \| \| Prupe.5G011300 \| Prupe.1G111900 \| \| Prupe.5G011400 \| Prupe.1G112000 \| \| Prupe.5G011500 \| Prupe.1G113600 \| \| Prupe.5G011600 \| Prupe.1G114500 \| \| Prupe.5G011700 \| Prupe.1G117800 \| \| Prupe.5G011800 \| Prupe.1G118200 \| \| Prupe.5G011900 \| Prupe.1G118300 \| \| Prupe.5G012000 \| Prupe.1G121200 \| \| Prupe.5G012100 \| Prupe.1G122800 \| \| Prupe.5G012200 \| Prupe.1G128600 \| \| Prupe.5G012300 \| Prupe.1G132800 \| \| Prupe.5G012400 \| Prupe.1G133100 \| \| Prupe.5G012500 \| Prupe.1G133200 \| \| Prupe.5G012600 \| Prupe.1G136400 \| \| Prupe.5G012700 \| Prupe.1G138300 \| \| Prupe.5G012800 \| Prupe.1G142100 \| \| Prupe.5G012900 \| Prupe.1G142500 \| \| Prupe.5G013000 \| Prupe.1G144400 \| \| Prupe.5G013100 \| Prupe.1G144500 \| \| Prupe.5G013200 \| Prupe.1G147900 \| \| Prupe.5G013300 \| Prupe.1G148000 \| \| Prupe.5G013400 \| Prupe.1G148700 \| \| Prupe.5G013500 \| Prupe.1G149600 \| \| Prupe.5G013600 \| Prupe.1G153000 \| \| Prupe.5G013700 \| Prupe.1G153600 \| \|  \| Prupe.1G154100 \| \|  \| Prupe.1G164000 \| \|  \| Prupe.1G165200 \| \|  \| Prupe.1G173200 \| \|  \| Prupe.1G174100 \| \|  \| Prupe.1G176100 \| \|  \| Prupe.1G177500 \| \|  \| Prupe.1G179700 \| \|  \| Prupe.1G182200 \| \|  \| Prupe.1G187700 \| \|  \| Prupe.1G187800 \| \|  \| Prupe.1G193000 \| \|  \| Prupe.1G194800 \| \|  \| Prupe.1G202600 \| \|  \| Prupe.1G203800 \| \|  \| Prupe.1G204800 \| \|  \| Prupe.1G205800 \| \|  \| Prupe.1G209800 \| \|  \| Prupe.1G210200 \| \|  \| Prupe.1G212800 \| \|  \| Prupe.1G213600 \| \|  \| Prupe.1G215400 \| \|  \| Prupe.1G217000 \| \|  \| Prupe.1G217100 \| \|  \| Prupe.1G218100 \| \|  \| Prupe.1G218700 \| \|  \| Prupe.1G219800 \| \|  \| Prupe.1G221900 \| \|  \| Prupe.1G225800 \| \|  \| Prupe.1G226800 \| \|  \| Prupe.1G230200 \| \|  \| Prupe.1G231900 \| \|  \| Prupe.1G235800 \| \|  \| Prupe.1G235900 \| \|  \| Prupe.1G239600 \| \|  \| Prupe.1G239700 \| \|  \| Prupe.1G241500 \| \|  \| Prupe.1G243100 \| \|  \| Prupe.1G244200 \| \|  \| Prupe.1G244300 \| \|  \| Prupe.1G244800 \| \|  \| Prupe.1G244900 \| \|  \| Prupe.1G247400 \| \|  \| Prupe.1G248000 \| \|  \| Prupe.1G251600 \| \|  \| Prupe.1G253800 \| \|  \| Prupe.1G255100 \| \|  \| Prupe.1G255500 \| \|  \| Prupe.1G257700 \| \|  \| Prupe.1G257800 \| \|  \| Prupe.1G259100 \| \|  \| Prupe.1G261700 \| \|  \| Prupe.1G262000 \| \|  \| Prupe.1G262400 \| \|  \| Prupe.1G262600 \| \|  \| Prupe.1G262700 \| \|  \| Prupe.1G271300 \| \|  \| Prupe.1G271400 \| \|  \| Prupe.1G272000 \| \|  \| Prupe.1G272500 \| \|  \| Prupe.1G275200 \| \|  \| Prupe.1G277200 \| \|  \| Prupe.1G278300 \| \|  \| Prupe.1G279300 \| \|  \| Prupe.1G282500 \| \|  \| Prupe.1G282700 \| \|  \| Prupe.1G282800 \| \|  \| Prupe.1G283600 \| \|  \| Prupe.1G286200 \| \|  \| Prupe.1G287500 \| \|  \| Prupe.1G288100 \| \|  \| Prupe.1G290800 \| \|  \| Prupe.1G291800 \| \|  \| Prupe.1G294800 \| \|  \| Prupe.1G295600 \| \|  \| Prupe.1G296900 \| \|  \| Prupe.1G297700 \| \|  \| Prupe.1G298000 \| \|  \| Prupe.1G301500 \| \|  \| Prupe.1G306400 \| \|  \| Prupe.1G306500 \| \|  \| Prupe.1G306700 \| \|  \| Prupe.1G309300 \| \|  \| Prupe.1G311100 \| \|  \| Prupe.1G311300 \| \|  \| Prupe.1G312400 \| \|  \| Prupe.1G312600 \| \|  \| Prupe.1G313100 \| \|  \| Prupe.1G313900 \| \|  \| Prupe.1G314000 \| \|  \| Prupe.1G314100 \| \|  \| Prupe.1G314300 \| \|  \| Prupe.1G318100 \| \|  \| Prupe.1G318400 \| \|  \| Prupe.1G322000 \| \|  \| Prupe.1G322100 \| \|  \| Prupe.1G323600 \| \|  \| Prupe.1G324900 \| \|  \| Prupe.1G325700 \| \|  \| Prupe.1G329300 \| \|  \| Prupe.1G329600 \| \|  \| Prupe.1G330400 \| \|  \| Prupe.1G334600 \| \|  \| Prupe.1G336300 \| \|  \| Prupe.1G338800 \| \|  \| Prupe.1G341100 \| \|  \| Prupe.1G342100 \| \|  \| Prupe.1G342600 \| \|  \| Prupe.1G343000 \| \|  \| Prupe.1G345400 \| \|  \| Prupe.1G345500 \| \|  \| Prupe.1G353100 \| \|  \| Prupe.1G353700 \| \|  \| Prupe.1G354100 \| \|  \| Prupe.1G355500 \| \|  \| Prupe.1G357500 \| \|  \| Prupe.1G357600 \| \|  \| Prupe.1G357700 \| \|  \| Prupe.1G358900 \| \|  \| Prupe.1G364200 \| \|  \| Prupe.1G364800 \| \|  \| Prupe.1G366400 \| \|  \| Prupe.1G366800 \| \|  \| Prupe.1G367000 \| \|  \| Prupe.1G370300 \| \|  \| Prupe.1G371300 \| \|  \| Prupe.1G376200 \| \|  \| Prupe.1G376300 \| \|  \| Prupe.1G378700 \| \|  \| Prupe.1G378900 \| \|  \| Prupe.1G380200 \| \|  \| Prupe.1G382200 \| \|  \| Prupe.1G382800 \| \|  \| Prupe.1G384500 \| \|  \| Prupe.1G386200 \| \|  \| Prupe.1G386300 \| \|  \| Prupe.1G386400 \| \|  \| Prupe.1G390500 \| \|  \| Prupe.1G392300 \| \|  \| Prupe.1G393300 \| \|  \| Prupe.1G393900 \| \|  \| Prupe.1G397200 \| \|  \| Prupe.1G403400 \| \|  \| Prupe.1G403900 \| \|  \| Prupe.1G404100 \| \|  \| Prupe.1G404700 \| \|  \| Prupe.1G405000 \| \|  \| Prupe.1G406100 \| \|  \| Prupe.1G406800 \| \|  \| Prupe.1G407300 \| \|  \| Prupe.1G410700 \| \|  \| Prupe.1G411000 \| \|  \| Prupe.1G411400 \| \|  \| Prupe.1G413600 \| \|  \| Prupe.1G413700 \| \|  \| Prupe.1G413800 \| \|  \| Prupe.1G413900 \| \|  \| Prupe.1G414800 \| \|  \| Prupe.1G415300 \| \|  \| Prupe.1G418400 \| \|  \| Prupe.1G419000 \| \|  \| Prupe.1G419100 \| \|  \| Prupe.1G419900 \| \|  \| Prupe.1G420400 \| \|  \| Prupe.1G426500 \| \|  \| Prupe.1G426700 \| \|  \| Prupe.1G427200 \| \|  \| Prupe.1G429600 \| \|  \| Prupe.1G429700 \| \|  \| Prupe.1G430500 \| \|  \| Prupe.1G432400 \| \|  \| Prupe.1G433900 \| \|  \| Prupe.1G445000 \| \|  \| Prupe.1G453100 \| \|  \| Prupe.1G455900 \| \|  \| Prupe.1G462100 \| \|  \| Prupe.1G462300 \| \|  \| Prupe.1G462400 \| \|  \| Prupe.1G462700 \| \|  \| Prupe.1G463400 \| \|  \| Prupe.1G472100 \| \|  \| Prupe.1G475200 \| \|  \| Prupe.1G476800 \| \|  \| Prupe.1G481700 \| \|  \| Prupe.1G483200 \| \|  \| Prupe.1G483800 \| \|  \| Prupe.1G485600 \| \|  \| Prupe.1G486500 \| \|  \| Prupe.1G486600 \| \|  \| Prupe.1G488000 \| \|  \| Prupe.1G488100 \| \|  \| Prupe.1G488600 \| \|  \| Prupe.1G488700 \| \|  \| Prupe.1G492300 \| \|  \| Prupe.1G493100 \| \|  \| Prupe.1G496500 \| \|  \| Prupe.1G497100 \| \|  \| Prupe.1G497200 \| \|  \| Prupe.1G497400 \| \|  \| Prupe.1G499700 \| \|  \| Prupe.1G501800 \| \|  \| Prupe.1G503100 \| \|  \| Prupe.1G504400 \| \|  \| Prupe.1G507100 \| \|  \| Prupe.1G508600 \| \|  \| Prupe.1G511600 \| \|  \| Prupe.1G512200 \| \|  \| Prupe.1G516200 \| \|  \| Prupe.1G518600 \| \|  \| Prupe.1G520400 \| \|  \| Prupe.1G521500 \| \|  \| Prupe.1G524800 \| \|  \| Prupe.1G524900 \| \|  \| Prupe.1G525000 \| \|  \| Prupe.1G525600 \| \|  \| Prupe.1G527300 \| \|  \| Prupe.1G528800 \| \|  \| Prupe.1G532400 \| \|  \| Prupe.1G533900 \| \|  \| Prupe.1G536600 \| \|  \| Prupe.1G540200 \| \|  \| Prupe.1G540700 \| \|  \| Prupe.1G540800 \| \|  \| Prupe.1G540900 \| \|  \| Prupe.1G541300 \| \|  \| Prupe.1G542700 \| \|  \| Prupe.1G543900 \| \|  \| Prupe.1G544100 \| \|  \| Prupe.1G544500 \| \|  \| Prupe.1G552500 \| \|  \| Prupe.1G552800 \| \|  \| Prupe.1G553300 \| \|  \| Prupe.1G553600 \| \|  \| Prupe.1G563000 \| \|  \| Prupe.1G563100 \| \|  \| Prupe.1G566600 \| \|  \| Prupe.1G568400 \| \|  \| Prupe.1G570800 \| \|  \| Prupe.1G571000 \| \|  \| Prupe.1G571100 \| \|  \| Prupe.1G575000 \| \|  \| Prupe.1G576300 \| \|  \| Prupe.1G576900 \| \|  \| Prupe.1G579400 \| \|  \| Prupe.2G000900 \| \|  \| Prupe.2G001800 \| \|  \| Prupe.2G002300 \| \|  \| Prupe.2G004100 \| \|  \| Prupe.2G004500 \| \|  \| Prupe.2G005300 \| \|  \| Prupe.2G005400 \| \|  \| Prupe.2G005500 \| \|  \| Prupe.2G008000 \| \|  \| Prupe.2G012000 \| \|  \| Prupe.2G012300 \| \|  \| Prupe.2G014500 \| \|  \| Prupe.2G019500 \| \|  \| Prupe.2G020100 \| \|  \| Prupe.2G021500 \| \|  \| Prupe.2G021700 \| \|  \| Prupe.2G022700 \| \|  \| Prupe.2G024600 \| \|  \| Prupe.2G029500 \| \|  \| Prupe.2G029800 \| \|  \| Prupe.2G032000 \| \|  \| Prupe.2G032200 \| \|  \| Prupe.2G032300 \| \|  \| Prupe.2G032600 \| \|  \| Prupe.2G033300 \| \|  \| Prupe.2G036500 \| \|  \| Prupe.2G040000 \| \|  \| Prupe.2G043400 \| \|  \| Prupe.2G045800 \| \|  \| Prupe.2G049000 \| \|  \| Prupe.2G051500 \| \|  \| Prupe.2G056200 \| \|  \| Prupe.2G070300 \| \|  \| Prupe.2G072100 \| \|  \| Prupe.2G075100 \| \|  \| Prupe.2G082800 \| \|  \| Prupe.2G085000 \| \|  \| Prupe.2G086600 \| \|  \| Prupe.2G090900 \| \|  \| Prupe.2G093700 \| \|  \| Prupe.2G097900 \| \|  \| Prupe.2G100800 \| \|  \| Prupe.2G101600 \| \|  \| Prupe.2G101800 \| \|  \| Prupe.2G101900 \| \|  \| Prupe.2G102700 \| \|  \| Prupe.2G103300 \| \|  \| Prupe.2G104200 \| \|  \| Prupe.2G107600 \| \|  \| Prupe.2G107900 \| \|  \| Prupe.2G108100 \| \|  \| Prupe.2G111700 \| \|  \| Prupe.2G111800 \| \|  \| Prupe.2G111900 \| \|  \| Prupe.2G112100 \| \|  \| Prupe.2G113800 \| \|  \| Prupe.2G114000 \| \|  \| Prupe.2G116600 \| \|  \| Prupe.2G118700 \| \|  \| Prupe.2G120300 \| \|  \| Prupe.2G125200 \| \|  \| Prupe.2G128400 \| \|  \| Prupe.2G131000 \| \|  \| Prupe.2G131900 \| \|  \| Prupe.2G132500 \| \|  \| Prupe.2G135700 \| \|  \| Prupe.2G140400 \| \|  \| Prupe.2G142300 \| \|  \| Prupe.2G147300 \| \|  \| Prupe.2G152600 \| \|  \| Prupe.2G155000 \| \|  \| Prupe.2G155400 \| \|  \| Prupe.2G156900 \| \|  \| Prupe.2G158600 \| \|  \| Prupe.2G161300 \| \|  \| Prupe.2G162700 \| \|  \| Prupe.2G167700 \| \|  \| Prupe.2G167900 \| \|  \| Prupe.2G170700 \| \|  \| Prupe.2G176500 \| \|  \| Prupe.2G177400 \| \|  \| Prupe.2G177700 \| \|  \| Prupe.2G179500 \| \|  \| Prupe.2G180600 \| \|  \| Prupe.2G182400 \| \|  \| Prupe.2G183400 \| \|  \| Prupe.2G184000 \| \|  \| Prupe.2G184400 \| \|  \| Prupe.2G187900 \| \|  \| Prupe.2G188000 \| \|  \| Prupe.2G190100 \| \|  \| Prupe.2G190300 \| \|  \| Prupe.2G190500 \| \|  \| Prupe.2G193300 \| \|  \| Prupe.2G193900 \| \|  \| Prupe.2G195100 \| \|  \| Prupe.2G198900 \| \|  \| Prupe.2G201600 \| \|  \| Prupe.2G201700 \| \|  \| Prupe.2G201800 \| \|  \| Prupe.2G201900 \| \|  \| Prupe.2G202000 \| \|  \| Prupe.2G207300 \| \|  \| Prupe.2G207600 \| \|  \| Prupe.2G207700 \| \|  \| Prupe.2G207800 \| \|  \| Prupe.2G208900 \| \|  \| Prupe.2G209000 \| \|  \| Prupe.2G210600 \| \|  \| Prupe.2G211800 \| \|  \| Prupe.2G212900 \| \|  \| Prupe.2G213300 \| \|  \| Prupe.2G217000 \| \|  \| Prupe.2G223500 \| \|  \| Prupe.2G223800 \| \|  \| Prupe.2G229000 \| \|  \| Prupe.2G230300 \| \|  \| Prupe.2G230400 \| \|  \| Prupe.2G231100 \| \|  \| Prupe.2G234500 \| \|  \| Prupe.2G235400 \| \|  \| Prupe.2G236500 \| \|  \| Prupe.2G238700 \| \|  \| Prupe.2G241700 \| \|  \| Prupe.2G241800 \| \|  \| Prupe.2G242600 \| \|  \| Prupe.2G243800 \| \|  \| Prupe.2G243900 \| \|  \| Prupe.2G245400 \| \|  \| Prupe.2G245700 \| \|  \| Prupe.2G251400 \| \|  \| Prupe.2G253200 \| \|  \| Prupe.2G258900 \| \|  \| Prupe.2G261600 \| \|  \| Prupe.2G263600 \| \|  \| Prupe.2G266100 \| \|  \| Prupe.2G270300 \| \|  \| Prupe.2G270400 \| \|  \| Prupe.2G275300 \| \|  \| Prupe.2G275500 \| \|  \| Prupe.2G277000 \| \|  \| Prupe.2G277800 \| \|  \| Prupe.2G278000 \| \|  \| Prupe.2G279500 \| \|  \| Prupe.2G279800 \| \|  \| Prupe.2G279900 \| \|  \| Prupe.2G282500 \| \|  \| Prupe.2G286300 \| \|  \| Prupe.2G288200 \| \|  \| Prupe.2G288800 \| \|  \| Prupe.2G289700 \| \|  \| Prupe.2G290300 \| \|  \| Prupe.2G291000 \| \|  \| Prupe.2G291100 \| \|  \| Prupe.2G291300 \| \|  \| Prupe.2G292000 \| \|  \| Prupe.2G295000 \| \|  \| Prupe.2G295300 \| \|  \| Prupe.2G296000 \| \|  \| Prupe.2G301700 \| \|  \| Prupe.2G302000 \| \|  \| Prupe.2G303900 \| \|  \| Prupe.2G304400 \| \|  \| Prupe.2G305000 \| \|  \| Prupe.2G306400 \| \|  \| Prupe.2G309000 \| \|  \| Prupe.2G310000 \| \|  \| Prupe.2G310300 \| \|  \| Prupe.2G314600 \| \|  \| Prupe.2G315200 \| \|  \| Prupe.2G315400 \| \|  \| Prupe.2G316500 \| \|  \| Prupe.2G319700 \| \|  \| Prupe.2G321700 \| \|  \| Prupe.2G323500 \| \|  \| Prupe.2G324700 \| \|  \| Prupe.3G002500 \| \|  \| Prupe.3G003200 \| \|  \| Prupe.3G004100 \| \|  \| Prupe.3G004800 \| \|  \| Prupe.3G008400 \| \|  \| Prupe.3G008900 \| \|  \| Prupe.3G011900 \| \|  \| Prupe.3G012000 \| \|  \| Prupe.3G013100 \| \|  \| Prupe.3G013200 \| \|  \| Prupe.3G014600 \| \|  \| Prupe.3G015000 \| \|  \| Prupe.3G016700 \| \|  \| Prupe.3G017100 \| \|  \| Prupe.3G018300 \| \|  \| Prupe.3G022700 \| \|  \| Prupe.3G027900 \| \|  \| Prupe.3G030600 \| \|  \| Prupe.3G033000 \| \|  \| Prupe.3G035500 \| \|  \| Prupe.3G036600 \| \|  \| Prupe.3G040900 \| \|  \| Prupe.3G043900 \| \|  \| Prupe.3G048500 \| \|  \| Prupe.3G048600 \| \|  \| Prupe.3G049200 \| \|  \| Prupe.3G049400 \| \|  \| Prupe.3G050500 \| \|  \| Prupe.3G053200 \| \|  \| Prupe.3G054300 \| \|  \| Prupe.3G057600 \| \|  \| Prupe.3G058300 \| \|  \| Prupe.3G062000 \| \|  \| Prupe.3G062800 \| \|  \| Prupe.3G063500 \| \|  \| Prupe.3G063700 \| \|  \| Prupe.3G064200 \| \|  \| Prupe.3G066300 \| \|  \| Prupe.3G066500 \| \|  \| Prupe.3G066900 \| \|  \| Prupe.3G067400 \| \|  \| Prupe.3G067900 \| \|  \| Prupe.3G068100 \| \|  \| Prupe.3G068200 \| \|  \| Prupe.3G071600 \| \|  \| Prupe.3G072900 \| \|  \| Prupe.3G074800 \| \|  \| Prupe.3G074900 \| \|  \| Prupe.3G076000 \| \|  \| Prupe.3G076400 \| \|  \| Prupe.3G077600 \| \|  \| Prupe.3G077700 \| \|  \| Prupe.3G083200 \| \|  \| Prupe.3G088700 \| \|  \| Prupe.3G089200 \| \|  \| Prupe.3G090800 \| \|  \| Prupe.3G091900 \| \|  \| Prupe.3G092500 \| \|  \| Prupe.3G094000 \| \|  \| Prupe.3G095500 \| \|  \| Prupe.3G096400 \| \|  \| Prupe.3G098800 \| \|  \| Prupe.3G100900 \| \|  \| Prupe.3G101400 \| \|  \| Prupe.3G101500 \| \|  \| Prupe.3G101900 \| \|  \| Prupe.3G102000 \| \|  \| Prupe.3G106900 \| \|  \| Prupe.3G113500 \| \|  \| Prupe.3G124300 \| \|  \| Prupe.3G127700 \| \|  \| Prupe.3G131900 \| \|  \| Prupe.3G135600 \| \|  \| Prupe.3G139800 \| \|  \| Prupe.3G142300 \| \|  \| Prupe.3G143800 \| \|  \| Prupe.3G145300 \| \|  \| Prupe.3G146400 \| \|  \| Prupe.3G148500 \| \|  \| Prupe.3G151200 \| \|  \| Prupe.3G151300 \| \|  \| Prupe.3G153200 \| \|  \| Prupe.3G153400 \| \|  \| Prupe.3G157100 \| \|  \| Prupe.3G157200 \| \|  \| Prupe.3G160200 \| \|  \| Prupe.3G160900 \| \|  \| Prupe.3G161500 \| \|  \| Prupe.3G162500 \| \|  \| Prupe.3G163100 \| \|  \| Prupe.3G163300 \| \|  \| Prupe.3G165400 \| \|  \| Prupe.3G165900 \| \|  \| Prupe.3G170700 \| \|  \| Prupe.3G173200 \| \|  \| Prupe.3G179400 \| \|  \| Prupe.3G179600 \| \|  \| Prupe.3G184400 \| \|  \| Prupe.3G187000 \| \|  \| Prupe.3G191500 \| \|  \| Prupe.3G192600 \| \|  \| Prupe.3G193600 \| \|  \| Prupe.3G195300 \| \|  \| Prupe.3G195400 \| \|  \| Prupe.3G196900 \| \|  \| Prupe.3G197000 \| \|  \| Prupe.3G197500 \| \|  \| Prupe.3G197600 \| \|  \| Prupe.3G198600 \| \|  \| Prupe.3G198900 \| \|  \| Prupe.3G199600 \| \|  \| Prupe.3G200500 \| \|  \| Prupe.3G200600 \| \|  \| Prupe.3G202400 \| \|  \| Prupe.3G202600 \| \|  \| Prupe.3G203100 \| \|  \| Prupe.3G209100 \| \|  \| Prupe.3G209200 \| \|  \| Prupe.3G211800 \| \|  \| Prupe.3G215800 \| \|  \| Prupe.3G217400 \| \|  \| Prupe.3G217500 \| \|  \| Prupe.3G221800 \| \|  \| Prupe.3G225700 \| \|  \| Prupe.3G226000 \| \|  \| Prupe.3G227100 \| \|  \| Prupe.3G229700 \| \|  \| Prupe.3G232200 \| \|  \| Prupe.3G235500 \| \|  \| Prupe.3G243500 \| \|  \| Prupe.3G245100 \| \|  \| Prupe.3G248200 \| \|  \| Prupe.3G249200 \| \|  \| Prupe.3G250000 \| \|  \| Prupe.3G251100 \| \|  \| Prupe.3G251900 \| \|  \| Prupe.3G252600 \| \|  \| Prupe.3G254700 \| \|  \| Prupe.3G254800 \| \|  \| Prupe.3G255500 \| \|  \| Prupe.3G256500 \| \|  \| Prupe.3G258100 \| \|  \| Prupe.3G259100 \| \|  \| Prupe.3G262300 \| \|  \| Prupe.3G262500 \| \|  \| Prupe.3G263100 \| \|  \| Prupe.3G264100 \| \|  \| Prupe.3G266700 \| \|  \| Prupe.3G268600 \| \|  \| Prupe.3G269500 \| \|  \| Prupe.3G269600 \| \|  \| Prupe.3G269700 \| \|  \| Prupe.3G271300 \| \|  \| Prupe.3G272200 \| \|  \| Prupe.3G272600 \| \|  \| Prupe.3G272800 \| \|  \| Prupe.3G273700 \| \|  \| Prupe.3G273800 \| \|  \| Prupe.3G274200 \| \|  \| Prupe.3G279000 \| \|  \| Prupe.3G284400 \| \|  \| Prupe.3G285300 \| \|  \| Prupe.3G285400 \| \|  \| Prupe.3G285500 \| \|  \| Prupe.3G285900 \| \|  \| Prupe.3G287100 \| \|  \| Prupe.3G293000 \| \|  \| Prupe.3G294100 \| \|  \| Prupe.3G294700 \| \|  \| Prupe.3G295800 \| \|  \| Prupe.3G296700 \| \|  \| Prupe.3G308800 \| \|  \| Prupe.3G315500 \| \|  \| Prupe.4G003900 \| \|  \| Prupe.4G004000 \| \|  \| Prupe.4G004900 \| \|  \| Prupe.4G005600 \| \|  \| Prupe.4G005700 \| \|  \| Prupe.4G005800 \| \|  \| Prupe.4G006300 \| \|  \| Prupe.4G006400 \| \|  \| Prupe.4G011000 \| \|  \| Prupe.4G013400 \| \|  \| Prupe.4G014100 \| \|  \| Prupe.4G016500 \| \|  \| Prupe.4G017900 \| \|  \| Prupe.4G019900 \| \|  \| Prupe.4G020800 \| \|  \| Prupe.4G022700 \| \|  \| Prupe.4G024600 \| \|  \| Prupe.4G025300 \| \|  \| Prupe.4G029300 \| \|  \| Prupe.4G030400 \| \|  \| Prupe.4G030900 \| \|  \| Prupe.4G031000 \| \|  \| Prupe.4G033900 \| \|  \| Prupe.4G036700 \| \|  \| Prupe.4G037200 \| \|  \| Prupe.4G037300 \| \|  \| Prupe.4G037400 \| \|  \| Prupe.4G042700 \| \|  \| Prupe.4G042800 \| \|  \| Prupe.4G046100 \| \|  \| Prupe.4G047400 \| \|  \| Prupe.4G047800 \| \|  \| Prupe.4G048200 \| \|  \| Prupe.4G048300 \| \|  \| Prupe.4G048900 \| \|  \| Prupe.4G049100 \| \|  \| Prupe.4G050300 \| \|  \| Prupe.4G052200 \| \|  \| Prupe.4G052400 \| \|  \| Prupe.4G053300 \| \|  \| Prupe.4G055900 \| \|  \| Prupe.4G067100 \| \|  \| Prupe.4G076500 \| \|  \| Prupe.4G079400 \| \|  \| Prupe.4G080700 \| \|  \| Prupe.4G081700 \| \|  \| Prupe.4G082000 \| \|  \| Prupe.4G084400 \| \|  \| Prupe.4G085200 \| \|  \| Prupe.4G089300 \| \|  \| Prupe.4G090200 \| \|  \| Prupe.4G090800 \| \|  \| Prupe.4G091300 \| \|  \| Prupe.4G091800 \| \|  \| Prupe.4G096600 \| \|  \| Prupe.4G096700 \| \|  \| Prupe.4G098300 \| \|  \| Prupe.4G103500 \| \|  \| Prupe.4G103600 \| \|  \| Prupe.4G104200 \| \|  \| Prupe.4G105400 \| \|  \| Prupe.4G105600 \| \|  \| Prupe.4G105700 \| \|  \| Prupe.4G121500 \| \|  \| Prupe.4G123000 \| \|  \| Prupe.4G123500 \| \|  \| Prupe.4G123600 \| \|  \| Prupe.4G123800 \| \|  \| Prupe.4G126200 \| \|  \| Prupe.4G130900 \| \|  \| Prupe.4G131700 \| \|  \| Prupe.4G134900 \| \|  \| Prupe.4G135000 \| \|  \| Prupe.4G135100 \| \|  \| Prupe.4G139400 \| \|  \| Prupe.4G143300 \| \|  \| Prupe.4G143400 \| \|  \| Prupe.4G148200 \| \|  \| Prupe.4G148300 \| \|  \| Prupe.4G148800 \| \|  \| Prupe.4G149300 \| \|  \| Prupe.4G150400 \| \|  \| Prupe.4G152700 \| \|  \| Prupe.4G156400 \| \|  \| Prupe.4G157300 \| \|  \| Prupe.4G157700 \| \|  \| Prupe.4G157800 \| \|  \| Prupe.4G157900 \| \|  \| Prupe.4G158000 \| \|  \| Prupe.4G162700 \| \|  \| Prupe.4G165200 \| \|  \| Prupe.4G173000 \| \|  \| Prupe.4G177100 \| \|  \| Prupe.4G181300 \| \|  \| Prupe.4G181700 \| \|  \| Prupe.4G182300 \| \|  \| Prupe.4G185700 \| \|  \| Prupe.4G187100 \| \|  \| Prupe.4G187400 \| \|  \| Prupe.4G190300 \| \|  \| Prupe.4G190600 \| \|  \| Prupe.4G192500 \| \|  \| Prupe.4G192800 \| \|  \| Prupe.4G195000 \| \|  \| Prupe.4G195800 \| \|  \| Prupe.4G200500 \| \|  \| Prupe.4G201200 \| \|  \| Prupe.4G201300 \| \|  \| Prupe.4G212100 \| \|  \| Prupe.4G216600 \| \|  \| Prupe.4G216700 \| \|  \| Prupe.4G216900 \| \|  \| Prupe.4G229700 \| \|  \| Prupe.4G230300 \| \|  \| Prupe.4G231100 \| \|  \| Prupe.4G231800 \| \|  \| Prupe.4G232400 \| \|  \| Prupe.4G233600 \| \|  \| Prupe.4G233700 \| \|  \| Prupe.4G234000 \| \|  \| Prupe.4G234200 \| \|  \| Prupe.4G234600 \| \|  \| Prupe.4G235300 \| \|  \| Prupe.4G235600 \| \|  \| Prupe.4G241000 \| \|  \| Prupe.4G248200 \| \|  \| Prupe.4G249900 \| \|  \| Prupe.4G250700 \| \|  \| Prupe.4G254900 \| \|  \| Prupe.4G259200 \| \|  \| Prupe.4G262500 \| \|  \| Prupe.4G266500 \| \|  \| Prupe.4G266800 \| \|  \| Prupe.4G269500 \| \|  \| Prupe.4G271700 \| \|  \| Prupe.4G271800 \| \|  \| Prupe.4G276400 \| \|  \| Prupe.4G278200 \| \|  \| Prupe.4G279800 \| \|  \| Prupe.4G281900 \| \|  \| Prupe.4G286100 \| \|  \| Prupe.5G001800 \| \|  \| Prupe.5G002200 \| \|  \| Prupe.5G002800 \| \|  \| Prupe.5G003300 \| \|  \| Prupe.5G006300 \| \|  \| Prupe.5G006400 \| \|  \| Prupe.5G009600 \| \|  \| Prupe.5G018200 \| \|  \| Prupe.5G021000 \| \|  \| Prupe.5G021500 \| \|  \| Prupe.5G021700 \| \|  \| Prupe.5G021900 \| \|  \| Prupe.5G024400 \| \|  \| Prupe.5G024600 \| \|  \| Prupe.5G027300 \| \|  \| Prupe.5G029300 \| \|  \| Prupe.5G033700 \| \|  \| Prupe.5G034700 \| \|  \| Prupe.5G035000 \| \|  \| Prupe.5G038500 \| \|  \| Prupe.5G039900 \| \|  \| Prupe.5G040700 \| \|  \| Prupe.5G041300 \| \|  \| Prupe.5G041700 \| \|  \| Prupe.5G044300 \| \|  \| Prupe.5G044400 \| \|  \| Prupe.5G045000 \| \|  \| Prupe.5G045200 \| \|  \| Prupe.5G045500 \| \|  \| Prupe.5G047800 \| \|  \| Prupe.5G052400 \| \|  \| Prupe.5G055000 \| \|  \| Prupe.5G061700 \| \|  \| Prupe.5G064500 \| \|  \| Prupe.5G064600 \| \|  \| Prupe.5G065500 \| \|  \| Prupe.5G065700 \| \|  \| Prupe.5G066100 \| \|  \| Prupe.5G068400 \| \|  \| Prupe.5G068700 \| \|  \| Prupe.5G069400 \| \|  \| Prupe.5G072200 \| \|  \| Prupe.5G072300 \| \|  \| Prupe.5G072500 \| \|  \| Prupe.5G073200 \| \|  \| Prupe.5G074800 \| \|  \| Prupe.5G075900 \| \|  \| Prupe.5G076000 \| \|  \| Prupe.5G077800 \| \|  \| Prupe.5G078200 \| \|  \| Prupe.5G081200 \| \|  \| Prupe.5G081300 \| \|  \| Prupe.5G086100 \| \|  \| Prupe.5G086700 \| \|  \| Prupe.5G088800 \| \|  \| Prupe.5G091500 \| \|  \| Prupe.5G092600 \| \|  \| Prupe.5G095200 \| \|  \| Prupe.5G100500 \| \|  \| Prupe.5G101200 \| \|  \| Prupe.5G101600 \| \|  \| Prupe.5G103500 \| \|  \| Prupe.5G104000 \| \|  \| Prupe.5G104500 \| \|  \| Prupe.5G107400 \| \|  \| Prupe.5G109300 \| \|  \| Prupe.5G110500 \| \|  \| Prupe.5G110800 \| \|  \| Prupe.5G112300 \| \|  \| Prupe.5G114400 \| \|  \| Prupe.5G114700 \| \|  \| Prupe.5G117900 \| \|  \| Prupe.5G118000 \| \|  \| Prupe.5G118300 \| \|  \| Prupe.5G118400 \| \|  \| Prupe.5G126300 \| \|  \| Prupe.5G129000 \| \|  \| Prupe.5G130400 \| \|  \| Prupe.5G131400 \| \|  \| Prupe.5G132200 \| \|  \| Prupe.5G133900 \| \|  \| Prupe.5G134900 \| \|  \| Prupe.5G135800 \| \|  \| Prupe.5G137800 \| \|  \| Prupe.5G138000 \| \|  \| Prupe.5G138500 \| \|  \| Prupe.5G142200 \| \|  \| Prupe.5G142600 \| \|  \| Prupe.5G142800 \| \|  \| Prupe.5G143100 \| \|  \| Prupe.5G143400 \| \|  \| Prupe.5G148500 \| \|  \| Prupe.5G150500 \| \|  \| Prupe.5G150700 \| \|  \| Prupe.5G161300 \| \|  \| Prupe.5G162000 \| \|  \| Prupe.5G165000 \| \|  \| Prupe.5G166300 \| \|  \| Prupe.5G167100 \| \|  \| Prupe.5G167500 \| \|  \| Prupe.5G169000 \| \|  \| Prupe.5G171400 \| \|  \| Prupe.5G172000 \| \|  \| Prupe.5G173800 \| \|  \| Prupe.5G175400 \| \|  \| Prupe.5G176800 \| \|  \| Prupe.5G176900 \| \|  \| Prupe.5G178200 \| \|  \| Prupe.5G181300 \| \|  \| Prupe.5G182000 \| \|  \| Prupe.5G184000 \| \|  \| Prupe.5G184300 \| \|  \| Prupe.5G186200 \| \|  \| Prupe.5G186300 \| \|  \| Prupe.5G189500 \| \|  \| Prupe.5G191200 \| \|  \| Prupe.5G199500 \| \|  \| Prupe.5G200900 \| \|  \| Prupe.5G204500 \| \|  \| Prupe.5G205100 \| \|  \| Prupe.5G206500 \| \|  \| Prupe.5G210100 \| \|  \| Prupe.5G213900 \| \|  \| Prupe.5G215700 \| \|  \| Prupe.5G215800 \| \|  \| Prupe.5G216300 \| \|  \| Prupe.5G218300 \| \|  \| Prupe.5G219700 \| \|  \| Prupe.5G221000 \| \|  \| Prupe.5G221200 \| \|  \| Prupe.5G224400 \| \|  \| Prupe.5G228300 \| \|  \| Prupe.5G233100 \| \|  \| Prupe.5G233300 \| \|  \| Prupe.5G233400 \| \|  \| Prupe.5G233500 \| \|  \| Prupe.5G235800 \| \|  \| Prupe.5G235900 \| \|  \| Prupe.5G238100 \| \|  \| Prupe.5G239500 \| \|  \| Prupe.5G240300 \| \|  \| Prupe.5G241300 \| \|  \| Prupe.5G242000 \| \|  \| Prupe.5G243300 \| \|  \| Prupe.5G243400 \| \|  \| Prupe.5G244000 \| \|  \| Prupe.5G244300 \| \|  \| Prupe.5G245400 \| \|  \| Prupe.6G000800 \| \|  \| Prupe.6G001600 \| \|  \| Prupe.6G002600 \| \|  \| Prupe.6G002900 \| \|  \| Prupe.6G003500 \| \|  \| Prupe.6G005900 \| \|  \| Prupe.6G008000 \| \|  \| Prupe.6G009000 \| \|  \| Prupe.6G009100 \| \|  \| Prupe.6G014500 \| \|  \| Prupe.6G023900 \| \|  \| Prupe.6G024300 \| \|  \| Prupe.6G024700 \| \|  \| Prupe.6G025100 \| \|  \| Prupe.6G026300 \| \|  \| Prupe.6G028100 \| \|  \| Prupe.6G028500 \| \|  \| Prupe.6G033600 \| \|  \| Prupe.6G035200 \| \|  \| Prupe.6G036400 \| \|  \| Prupe.6G039700 \| \|  \| Prupe.6G045000 \| \|  \| Prupe.6G047300 \| \|  \| Prupe.6G049900 \| \|  \| Prupe.6G051800 \| \|  \| Prupe.6G052400 \| \|  \| Prupe.6G053300 \| \|  \| Prupe.6G053900 \| \|  \| Prupe.6G056700 \| \|  \| Prupe.6G058100 \| \|  \| Prupe.6G058200 \| \|  \| Prupe.6G059600 \| \|  \| Prupe.6G061000 \| \|  \| Prupe.6G063500 \| \|  \| Prupe.6G067400 \| \|  \| Prupe.6G069000 \| \|  \| Prupe.6G069600 \| \|  \| Prupe.6G070500 \| \|  \| Prupe.6G072700 \| \|  \| Prupe.6G074000 \| \|  \| Prupe.6G075100 \| \|  \| Prupe.6G077200 \| \|  \| Prupe.6G077700 \| \|  \| Prupe.6G077800 \| \|  \| Prupe.6G077900 \| \|  \| Prupe.6G080200 \| \|  \| Prupe.6G085300 \| \|  \| Prupe.6G085800 \| \|  \| Prupe.6G087100 \| \|  \| Prupe.6G090800 \| \|  \| Prupe.6G091800 \| \|  \| Prupe.6G092300 \| \|  \| Prupe.6G100200 \| \|  \| Prupe.6G100700 \| \|  \| Prupe.6G101600 \| \|  \| Prupe.6G112200 \| \|  \| Prupe.6G114800 \| \|  \| Prupe.6G118500 \| \|  \| Prupe.6G119800 \| \|  \| Prupe.6G130100 \| \|  \| Prupe.6G130200 \| \|  \| Prupe.6G131600 \| \|  \| Prupe.6G131700 \| \|  \| Prupe.6G135000 \| \|  \| Prupe.6G135100 \| \|  \| Prupe.6G137400 \| \|  \| Prupe.6G143700 \| \|  \| Prupe.6G144100 \| \|  \| Prupe.6G148600 \| \|  \| Prupe.6G149800 \| \|  \| Prupe.6G156200 \| \|  \| Prupe.6G156300 \| \|  \| Prupe.6G156400 \| \|  \| Prupe.6G163700 \| \|  \| Prupe.6G166000 \| \|  \| Prupe.6G168000 \| \|  \| Prupe.6G169400 \| \|  \| Prupe.6G176700 \| \|  \| Prupe.6G177500 \| \|  \| Prupe.6G186900 \| \|  \| Prupe.6G187500 \| \|  \| Prupe.6G189800 \| \|  \| Prupe.6G190000 \| \|  \| Prupe.6G193100 \| \|  \| Prupe.6G193900 \| \|  \| Prupe.6G194700 \| \|  \| Prupe.6G196300 \| \|  \| Prupe.6G199400 \| \|  \| Prupe.6G199600 \| \|  \| Prupe.6G199700 \| \|  \| Prupe.6G201400 \| \|  \| Prupe.6G202100 \| \|  \| Prupe.6G203500 \| \|  \| Prupe.6G204700 \| \|  \| Prupe.6G209100 \| \|  \| Prupe.6G209500 \| \|  \| Prupe.6G210500 \| \|  \| Prupe.6G212200 \| \|  \| Prupe.6G214800 \| \|  \| Prupe.6G216100 \| \|  \| Prupe.6G216700 \| \|  \| Prupe.6G216800 \| \|  \| Prupe.6G221800 \| \|  \| Prupe.6G222000 \| \|  \| Prupe.6G223500 \| \|  \| Prupe.6G224600 \| \|  \| Prupe.6G224700 \| \|  \| Prupe.6G225400 \| \|  \| Prupe.6G228200 \| \|  \| Prupe.6G228900 \| \|  \| Prupe.6G230400 \| \|  \| Prupe.6G232000 \| \|  \| Prupe.6G233700 \| \|  \| Prupe.6G234500 \| \|  \| Prupe.6G235800 \| \|  \| Prupe.6G237800 \| \|  \| Prupe.6G237900 \| \|  \| Prupe.6G238000 \| \|  \| Prupe.6G238100 \| \|  \| Prupe.6G238500 \| \|  \| Prupe.6G244300 \| \|  \| Prupe.6G246000 \| \|  \| Prupe.6G246700 \| \|  \| Prupe.6G247400 \| \|  \| Prupe.6G251300 \| \|  \| Prupe.6G251400 \| \|  \| Prupe.6G251500 \| \|  \| Prupe.6G251700 \| \|  \| Prupe.6G252000 \| \|  \| Prupe.6G255100 \| \|  \| Prupe.6G258300 \| \|  \| Prupe.6G259200 \| \|  \| Prupe.6G261200 \| \|  \| Prupe.6G261300 \| \|  \| Prupe.6G261600 \| \|  \| Prupe.6G262400 \| \|  \| Prupe.6G264000 \| \|  \| Prupe.6G264700 \| \|  \| Prupe.6G265200 \| \|  \| Prupe.6G267500 \| \|  \| Prupe.6G270200 \| \|  \| Prupe.6G273000 \| \|  \| Prupe.6G278000 \| \|  \| Prupe.6G282200 \| \|  \| Prupe.6G283900 \| \|  \| Prupe.6G284000 \| \|  \| Prupe.6G284800 \| \|  \| Prupe.6G284900 \| \|  \| Prupe.6G287600 \| \|  \| Prupe.6G288800 \| \|  \| Prupe.6G290900 \| \|  \| Prupe.6G291400 \| \|  \| Prupe.6G293800 \| \|  \| Prupe.6G294000 \| \|  \| Prupe.6G294800 \| \|  \| Prupe.6G296500 \| \|  \| Prupe.6G296800 \| \|  \| Prupe.6G299600 \| \|  \| Prupe.6G301200 \| \|  \| Prupe.6G302500 \| \|  \| Prupe.6G303100 \| \|  \| Prupe.6G303200 \| \|  \| Prupe.6G305000 \| \|  \| Prupe.6G305300 \| \|  \| Prupe.6G306200 \| \|  \| Prupe.6G310300 \| \|  \| Prupe.6G314100 \| \|  \| Prupe.6G314600 \| \|  \| Prupe.6G317900 \| \|  \| Prupe.6G318400 \| \|  \| Prupe.6G324100 \| \|  \| Prupe.6G324400 \| \|  \| Prupe.6G324600 \| \|  \| Prupe.6G325100 \| \|  \| Prupe.6G325200 \| \|  \| Prupe.6G326500 \| \|  \| Prupe.6G330400 \| \|  \| Prupe.6G330500 \| \|  \| Prupe.6G332300 \| \|  \| Prupe.6G336400 \| \|  \| Prupe.6G336500 \| \|  \| Prupe.6G336700 \| \|  \| Prupe.6G339400 \| \|  \| Prupe.6G340000 \| \|  \| Prupe.6G340300 \| \|  \| Prupe.6G340400 \| \|  \| Prupe.6G341400 \| \|  \| Prupe.6G342000 \| \|  \| Prupe.6G343800 \| \|  \| Prupe.6G345400 \| \|  \| Prupe.6G345800 \| \|  \| Prupe.6G349500 \| \|  \| Prupe.6G350200 \| \|  \| Prupe.6G350300 \| \|  \| Prupe.6G350700 \| \|  \| Prupe.6G352500 \| \|  \| Prupe.6G353000 \| \|  \| Prupe.6G357600 \| \|  \| Prupe.6G358000 \| \|  \| Prupe.6G361000 \| \|  \| Prupe.6G362400 \| \|  \| Prupe.6G364700 \| \|  \| Prupe.6G365100 \| \|  \| Prupe.7G009700 \| \|  \| Prupe.7G009800 \| \|  \| Prupe.7G011100 \| \|  \| Prupe.7G017900 \| \|  \| Prupe.7G018300 \| \|  \| Prupe.7G021900 \| \|  \| Prupe.7G022500 \| \|  \| Prupe.7G022600 \| \|  \| Prupe.7G026500 \| \|  \| Prupe.7G029500 \| \|  \| Prupe.7G029600 \| \|  \| Prupe.7G029800 \| \|  \| Prupe.7G032400 \| \|  \| Prupe.7G034300 \| \|  \| Prupe.7G038600 \| \|  \| Prupe.7G041200 \| \|  \| Prupe.7G049700 \| \|  \| Prupe.7G050500 \| \|  \| Prupe.7G057000 \| \|  \| Prupe.7G058900 \| \|  \| Prupe.7G059000 \| \|  \| Prupe.7G059300 \| \|  \| Prupe.7G067900 \| \|  \| Prupe.7G068000 \| \|  \| Prupe.7G070700 \| \|  \| Prupe.7G072500 \| \|  \| Prupe.7G073200 \| \|  \| Prupe.7G076700 \| \|  \| Prupe.7G077900 \| \|  \| Prupe.7G078000 \| \|  \| Prupe.7G079900 \| \|  \| Prupe.7G086100 \| \|  \| Prupe.7G086200 \| \|  \| Prupe.7G089100 \| \|  \| Prupe.7G090300 \| \|  \| Prupe.7G091300 \| \|  \| Prupe.7G092100 \| \|  \| Prupe.7G093100 \| \|  \| Prupe.7G093200 \| \|  \| Prupe.7G095600 \| \|  \| Prupe.7G096100 \| \|  \| Prupe.7G096200 \| \|  \| Prupe.7G098000 \| \|  \| Prupe.7G098200 \| \|  \| Prupe.7G098700 \| \|  \| Prupe.7G099800 \| \|  \| Prupe.7G100000 \| \|  \| Prupe.7G102200 \| \|  \| Prupe.7G103500 \| \|  \| Prupe.7G103900 \| \|  \| Prupe.7G107700 \| \|  \| Prupe.7G108100 \| \|  \| Prupe.7G108200 \| \|  \| Prupe.7G108500 \| \|  \| Prupe.7G109600 \| \|  \| Prupe.7G111400 \| \|  \| Prupe.7G112200 \| \|  \| Prupe.7G114600 \| \|  \| Prupe.7G122200 \| \|  \| Prupe.7G123600 \| \|  \| Prupe.7G125200 \| \|  \| Prupe.7G125600 \| \|  \| Prupe.7G128400 \| \|  \| Prupe.7G128500 \| \|  \| Prupe.7G128600 \| \|  \| Prupe.7G131500 \| \|  \| Prupe.7G131900 \| \|  \| Prupe.7G133100 \| \|  \| Prupe.7G134600 \| \|  \| Prupe.7G136400 \| \|  \| Prupe.7G137500 \| \|  \| Prupe.7G137900 \| \|  \| Prupe.7G138900 \| \|  \| Prupe.7G139500 \| \|  \| Prupe.7G141800 \| \|  \| Prupe.7G141900 \| \|  \| Prupe.7G142100 \| \|  \| Prupe.7G143900 \| \|  \| Prupe.7G145200 \| \|  \| Prupe.7G148200 \| \|  \| Prupe.7G148300 \| \|  \| Prupe.7G148800 \| \|  \| Prupe.7G150000 \| \|  \| Prupe.7G151500 \| \|  \| Prupe.7G152700 \| \|  \| Prupe.7G157600 \| \|  \| Prupe.7G158400 \| \|  \| Prupe.7G158900 \| \|  \| Prupe.7G160800 \| \|  \| Prupe.7G161100 \| \|  \| Prupe.7G161700 \| \|  \| Prupe.7G163900 \| \|  \| Prupe.7G165400 \| \|  \| Prupe.7G166400 \| \|  \| Prupe.7G167000 \| \|  \| Prupe.7G170500 \| \|  \| Prupe.7G171600 \| \|  \| Prupe.7G173300 \| \|  \| Prupe.7G174800 \| \|  \| Prupe.7G176300 \| \|  \| Prupe.7G181200 \| \|  \| Prupe.7G183200 \| \|  \| Prupe.7G184200 \| \|  \| Prupe.7G186300 \| \|  \| Prupe.7G187700 \| \|  \| Prupe.7G188000 \| \|  \| Prupe.7G188400 \| \|  \| Prupe.7G188500 \| \|  \| Prupe.7G190400 \| \|  \| Prupe.7G192500 \| \|  \| Prupe.7G192800 \| \|  \| Prupe.7G192900 \| \|  \| Prupe.7G198400 \| \|  \| Prupe.7G202200 \| \|  \| Prupe.7G202600 \| \|  \| Prupe.7G205600 \| \|  \| Prupe.7G209100 \| \|  \| Prupe.7G210700 \| \|  \| Prupe.7G211300 \| \|  \| Prupe.7G211700 \| \|  \| Prupe.7G211800 \| \|  \| Prupe.7G212600 \| \|  \| Prupe.7G212700 \| \|  \| Prupe.7G212800 \| \|  \| Prupe.7G213100 \| \|  \| Prupe.7G215900 \| \|  \| Prupe.7G216900 \| \|  \| Prupe.7G217700 \| \|  \| Prupe.7G218000 \| \|  \| Prupe.7G218800 \| \|  \| Prupe.7G219800 \| \|  \| Prupe.7G219900 \| \|  \| Prupe.7G222100 \| \|  \| Prupe.7G222600 \| \|  \| Prupe.7G228000 \| \|  \| Prupe.7G230400 \| \|  \| Prupe.7G232200 \| \|  \| Prupe.7G232500 \| \|  \| Prupe.7G233100 \| \|  \| Prupe.7G240800 \| \|  \| Prupe.7G242200 \| \|  \| Prupe.7G242700 \| \|  \| Prupe.7G243300 \| \|  \| Prupe.7G243400 \| \|  \| Prupe.7G245300 \| \|  \| Prupe.7G245400 \| \|  \| Prupe.7G253100 \| \|  \| Prupe.7G253500 \| \|  \| Prupe.7G253800 \| \|  \| Prupe.7G255400 \| \|  \| Prupe.7G255500 \| \|  \| Prupe.7G256100 \| \|  \| Prupe.7G257900 \| \|  \| Prupe.7G259700 \| \|  \| Prupe.7G264300 \| \|  \| Prupe.7G264900 \| \|  \| Prupe.7G266500 \| \|  \| Prupe.7G267600 \| \|  \| Prupe.7G268400 \| \|  \| Prupe.7G270900 \| \|  \| Prupe.8G001800 \| \|  \| Prupe.8G002100 \| \|  \| Prupe.8G005400 \| \|  \| Prupe.8G006900 \| \|  \| Prupe.8G016800 \| \|  \| Prupe.8G016900 \| \|  \| Prupe.8G019400 \| \|  \| Prupe.8G022200 \| \|  \| Prupe.8G023000 \| \|  \| Prupe.8G023100 \| \|  \| Prupe.8G023400 \| \|  \| Prupe.8G023500 \| \|  \| Prupe.8G028000 \| \|  \| Prupe.8G028800 \| \|  \| Prupe.8G030300 \| \|  \| Prupe.8G033500 \| \|  \| Prupe.8G034400 \| \|  \| Prupe.8G034500 \| \|  \| Prupe.8G034700 \| \|  \| Prupe.8G037500 \| \|  \| Prupe.8G038600 \| \|  \| Prupe.8G038900 \| \|  \| Prupe.8G044100 \| \|  \| Prupe.8G044400 \| \|  \| Prupe.8G051900 \| \|  \| Prupe.8G058500 \| \|  \| Prupe.8G058600 \| \|  \| Prupe.8G061700 \| \|  \| Prupe.8G062200 \| \|  \| Prupe.8G064500 \| \|  \| Prupe.8G068900 \| \|  \| Prupe.8G069400 \| \|  \| Prupe.8G077100 \| \|  \| Prupe.8G080100 \| \|  \| Prupe.8G080300 \| \|  \| Prupe.8G081000 \| \|  \| Prupe.8G082200 \| \|  \| Prupe.8G083000 \| \|  \| Prupe.8G083800 \| \|  \| Prupe.8G085100 \| \|  \| Prupe.8G085400 \| \|  \| Prupe.8G086300 \| \|  \| Prupe.8G086900 \| \|  \| Prupe.8G087200 \| \|  \| Prupe.8G088700 \| \|  \| Prupe.8G089900 \| \|  \| Prupe.8G090400 \| \|  \| Prupe.8G091600 \| \|  \| Prupe.8G092500 \| \|  \| Prupe.8G093000 \| \|  \| Prupe.8G093200 \| \|  \| Prupe.8G093700 \| \|  \| Prupe.8G096000 \| \|  \| Prupe.8G096900 \| \|  \| Prupe.8G097900 \| \|  \| Prupe.8G098800 \| \|  \| Prupe.8G100600 \| \|  \| Prupe.8G104800 \| \|  \| Prupe.8G106300 \| \|  \| Prupe.8G112900 \| \|  \| Prupe.8G113200 \| \|  \| Prupe.8G115900 \| \|  \| Prupe.8G120900 \| \|  \| Prupe.8G122100 \| \|  \| Prupe.8G125600 \| \|  \| Prupe.8G127200 \| \|  \| Prupe.8G128100 \| \|  \| Prupe.8G129500 \| \|  \| Prupe.8G137500 \| \|  \| Prupe.8G137900 \| \|  \| Prupe.8G143000 \| \|  \| Prupe.8G143200 \| \|  \| Prupe.8G144800 \| \|  \| Prupe.8G147200 \| \|  \| Prupe.8G151400 \| \|  \| Prupe.8G157300 \| \|  \| Prupe.8G157400 \| \|  \| Prupe.8G161700 \| \|  \| Prupe.8G162800 \| \|  \| Prupe.8G163200 \| \|  \| Prupe.8G165200 \| \|  \| Prupe.8G166000 \| \|  \| Prupe.8G166400 \| \|  \| Prupe.8G168500 \| \|  \| Prupe.8G170800 \| \|  \| Prupe.8G172900 \| \|  \| Prupe.8G173600 \| \|  \| Prupe.8G173700 \| \|  \| Prupe.8G176200 \| \|  \| Prupe.8G176700 \| \|  \| Prupe.8G191400 \| \|  \| Prupe.8G192400 \| \|  \| Prupe.8G192800 \| \|  \| Prupe.8G193500 \| \|  \| Prupe.8G193800 \| \|  \| Prupe.8G194000 \| \|  \| Prupe.8G195200 \| \|  \| Prupe.8G197300 \| \|  \| Prupe.8G198300 \| \|  \| Prupe.8G202300 \| \|  \| Prupe.8G208900 \| \|  \| Prupe.8G210000 \| \|  \| Prupe.8G210100 \| \|  \| Prupe.8G212600 \| \|  \| Prupe.8G215400 \| \|  \| Prupe.8G215800 \| \|  \| Prupe.8G216400 \| \|  \| Prupe.8G219200 \| \|  \| Prupe.8G225500 \| \|  \| Prupe.8G225600 \| \|  \| Prupe.8G229400 \| \|  \| Prupe.8G229800 \| \|  \| Prupe.8G230500 \| \|  \| Prupe.8G231600 \| \|  \| Prupe.8G232200 \| \|  \| Prupe.8G232400 \| \|  \| Prupe.8G235400 \| \|  \| Prupe.8G237300 \| \|  \| Prupe.8G239400 \| \|  \| Prupe.8G239500 \| \|  \| Prupe.8G239600 \| \|  \| Prupe.8G241400 \| \|  \| Prupe.8G241500 \| \|  \| Prupe.8G243900 \| \|  \| Prupe.8G244900 \| \|  \| Prupe.8G248100 \| \|  \| Prupe.8G250600 \| \|  \| Prupe.8G250700 \| \|  \| Prupe.8G252100 \| \|  \| Prupe.8G252500 \| \|  \| Prupe.8G256900 \| \|  \| Prupe.8G257900 \| \|  \| Prupe.8G259100 \| \|  \| Prupe.8G259200 \| \|  \| Prupe.8G260100 \| \|  \| Prupe.8G262000 \| \|  \| Prupe.8G262100 \| \|  \| Prupe.8G262800 \| \|  \| Prupe.8G264300 \| \|  \| Prupe.8G268600 \| \|  \| Prupe.8G270800 \| | | | | | |
|  | |  | | |  |

| \| **Table S4-1. Summary of peach fruits transcriptome data. In this table, A_X-Y means A represent stage, X represent variety (H: ‘Hakuho’, T: ‘Tianjin Shui Mi’, Y represent biological replicates serial number.** \| \| \| \| \| \| \| --- \| --- \| --- \| --- \| --- \| --- \| \| \| Sample \| Clean Reads \| Clean Bases \| Ref. Genome ‘Lovell’ (Mb) \| Mapped Reads \| Mapping Rate (%) \| \| S1_H-1&2 \| 39379040 \| 4922380000 \| 265 \| 35756168 \| 90.80% \| \| S2_H-1&2 \| 44888292 \| 5611036500 \| 265 \| 40309686 \| 89.80% \| \| S3_H-1&2 \| 40088416 \| 5011052000 \| 265 \| 36440370 \| 90.90% \| \| S4_H-1&2 \| 37122810 \| 4640351250 \| 265 \| 34041616 \| 91.70% \| \| S5_H-1&2 \| 34734934 \| 4341866750 \| 265 \| 32129813 \| 92.50% \| \| S6_H-1&2 \| 32939420 \| 4117427500 \| 265 \| 30436024 \| 92.40% \| \| S1_T-1&2 \| 33412272 \| 4176534000 \| 265 \| 30104457 \| 90.10% \| \| S2_T-1&2 \| 38769676 \| 4846209500 \| 265 \| 34776399 \| 89.70% \| \| S3_T-1&2 \| 42265362 \| 5283170250 \| 265 \| 38250152 \| 90.50% \| \| S4_T-1&2 \| 29749172 \| 3718646500 \| 265 \| 27279990 \| 91.70% \| \| S5_T-1&2 \| 39384568 \| 4923071000 \| 265 \| 36391340 \| 92.40% \| \| S6_T-1&2 \| 40144484 \| 5018060500 \| 265 \| 37454803 \| 93.30% \| | | | | | |
| --- | --- | --- | --- | --- | --- | --- | --- | --- | --- | --- | --- | --- | --- | --- | --- | --- | --- | --- | --- | --- | --- | --- | --- | --- | --- | --- | --- | --- | --- | --- | --- | --- | --- | --- | --- | --- | --- | --- | --- | --- | --- | --- | --- | --- | --- | --- | --- | --- | --- | --- | --- | --- | --- | --- | --- | --- | --- | --- | --- | --- | --- | --- | --- | --- | --- | --- | --- | --- | --- | --- | --- | --- | --- | --- | --- | --- | --- | --- | --- | --- | --- | --- | --- | --- | --- | --- | --- | --- | --- |
|  |  |  |  |  |  |
| \| **Table S4-2. Summary of BSA-seq analysis data.** \| \| \| \| \| \| --- \| --- \| --- \| --- \| --- \| \| \| Sample \| Non-acid bulks \| Acid bulks \| Yangzhou 431 \| Huang 07-4-28 \| \| Clean reads \| 141474362 \| 140540432 \| 147,876,374 \| 287246278 \| \| Clean bases(bp) \| 21221154300 \| 21081064800 \| 22,181,456,100 \| 43086941700 \| \| Mapped reads \| 136469353 \| 136371015 \| 145,770,938 \| 280791483 \| \| Mapped bases(bp) \| 20364564688 \| 20359149647 \| 21,628,301,852 \| 41946933430 \| \| Mapping rate \| 0.9646 \| 0.9703 \| 1 \| 0.9775 \| \| Duplicate reads \| 25836679 \| 26342972 \| 21,895,927 \| 60084977 \| \| Duplicate rate \| 0.1893 \| 0.1932 \| 0 \| 0.214 \| \| Mismatch bases(bp) \| 139823408 \| 137907117 \| 138,449,273 \| 251912722 \| \| Mismatch rate \| 0.0069 \| 0.0068 \| 0 \| 0.006 \| \| Average sequencing depth \| 68.74 \| 69.18 \| 80 \| 137.58 \| \| Coverage \| 0.9971 \| 0.9962 \| 1 \| 0.9952 \| \| Coverage at least 4X \| 0.9956 \| 0.994 \| 1 \| 0.9936 \| \| Coverage at least 10X \| 0.9917 \| 0.987 \| 1 \| 0.9918 \| \| Coverage at least 20X \| 0.9789 \| 0.9734 \| 1 \| 0.9897 \| \| Coverage at least 50X \| 0.7477 \| 0.7624 \| 1 \| 0.98 \| |  |  |  |  |  |
| \| **Table S5. Interaction proteins of PpTST1 identified by DUALmembrane yeast two-hybrid system. In this table, common means those both interacting with PpTST1^Gln^ and PpTST1^His^，PpTST1^Gln^ means those only interacting with PpTST1^Gln^, PpTST1^His^ means those only interacting with PpTST1^His^.** \| \| \| \| --- \| --- \| --- \| \| \| **Groups** \| **BLAS  output from prey clone sequencing** \| **Genes encoding interaction proteins** \| \| common \| Populus EST from mild drought-stressed leaves \|  \| \| common \| PREDICTED: Prunus dulcis post-GPI attachment to proteins factor 3 (LOC117625112), mRNA \|  \| \| common \| PREDICTED: Prunus dulcis transcription factor MAMYB (LOC117628834), transcript variant X1, mRNA \|  \| \| common \| PREDICTED: Prunus persica 21 kDa protein (LOC18769631), mRNA \| Prupe.7G193700 \| \| common \| PREDICTED: Prunus persica aquaporin PIP1-2 (LOC18786880), mRNA \| Prupe.2G171200 \| \| common \| PREDICTED: Prunus persica aquaporin SIP1-1 (LOC18782318), mRNA \| Prupe.3G216100 \| \| common \| PREDICTED: Prunus persica cold-regulated 413 inner membrane protein 2, chloroplastic (LOC18779742), mRNA \| Prupe.4G074300 \| \| common \| PREDICTED: Prunus persica cysteine proteinase inhibitor 12 (LOC18785448), mRNA \| Prupe.2G047100 \| \| common \| PREDICTED: Prunus persica dnaJ homolog subfamily C member 2 (LOC18776590), transcript variant X2, mRNA \| Prupe.5G047100 \| \| common \| PREDICTED: Prunus persica fruit protein pKIWI501 (LOC18781341), mRNA \| Prupe.4G009800 \| \| common \| PREDICTED: Prunus persica LRR receptor kinase BAK1 (LOC18766377), mRNA \| Prupe.8G140900 \| \| common \| PREDICTED: Prunus persica lysine histidine transporter 1 (LOC18781720), mRNA \| Prupe.3G300900 \| \| common \| PREDICTED: Prunus persica mitochondrial fission 1 protein A (LOC18780050), mRNA \| Prupe.4G258900 \| \| common \| PREDICTED: Prunus persica photosystem I reaction center subunit XI, chloroplastic (LOC18773231), mRNA \| Prupe.6G361000 \| \| common \| PREDICTED: Prunus persica plasma membrane-associated cation-binding protein 1 (LOC18781369), transcript variant X1, mRNA \| Prupe.4G121100 \| \| common \| PREDICTED: Prunus persica probable aquaporin PIP1-4 (LOC18785337), mRNA \| Prupe.2G247300 \| \| common \| PREDICTED: Prunus persica rhomboid-like protein 20 (LOC18769142), mRNA \| Prupe.7G034400 \| \| common \| PREDICTED: Prunus persica transmembrane protein 18 (LOC18768763), mRNA \| Prupe.8G054100 \| \| common \| PREDICTED: Prunus persica uncharacterized LOC18780412 (LOC18780412), mRNA \| Prupe.4G047900 \| \| common \| PREDICTED: Prunus persica uncharacterized LOC18783005 (LOC18783005), mRNA \| Prupe.3G070800 \| \| common \| PREDICTED: Prunus persica uncharacterized LOC18789752 (LOC18789752), mRNA \| Prupe.1G050700 \| \| common \| PREDICTED: Prunus persica uncharacterized LOC18790790 (LOC18790790), mRNA \| Prupe.1G478100 \| \| common \| PREDICTED: Prunus persica uncharacterized LOC18792000 (LOC18792000), mRNA \| Prupe.1G035100 \| \| common \| PREDICTED: Prunus persica uncharacterized LOC18792339 (LOC18792339), mRNA \| Prupe.1G035200 \| \| common \| PREDICTED: Prunus persica uncharacterized protein C1450.15 (LOC18781784), transcript variant X1, mRNA \| Prupe.3G103300 \| \| common \| PREDICTED: Prunus persica vesicle-associated protein 1-2 (LOC18784918), mRNA \| Prupe.2G138100 \| \| common \| PREDICTED: Prunus persica V-type proton ATPase 16 kDa proteolipid subunit (LOC18768326), mRNA \| Prupe.8G083200 \| \| common \| PREDICTED: Prunus persica V-type proton ATPase 16 kDa proteolipid subunit (LOC18771680), mRNA \| Prupe.7G166200 \| \| common \| PREDICTED: Prunus persica WAT1-related protein At4g19185 (LOC18790641), mRNA \| Prupe.1G353200 \| \| common \| PREDICTED: Rosa chinensis CCG-binding protein 1 (LOC112173399), mRNA \| RchiOBHm_Chr6g0259611 \| \| common \| Prunus dulcis DNA, pseudomolecule Pd06 \|  \| \| common \| Salmo salar clone ssal-evd-541-186 60S acidic ribosomal protein P1 putative mRNA, complete cds \|  \| \| PpTST1^Gln^ \| Aphanomyces euteiches cDNA \|  \| \| PpTST1^Gln^ \| Bufo marinus mRNA for epithelial sodium channel alpha subunit, complete cds \|  \| \| PpTST1^Gln^ \| Populus EST from leave \|  \| \| PpTST1^Gln^ \| PREDICTED: Prunus persica cell division topological specificity factor homolog, chloroplastic (LOC18790183), mRNA \| Prupe.1G281100 \| \| PpTST1^Gln^ \| PREDICTED: Prunus persica glutathione S-transferase F12 (LOC18782821), mRNA \| Prupe.3G013600 \| \| PpTST1^Gln^ \| PREDICTED: Prunus persica probable carboxylesterase SOBER1-like (LOC18779014), mRNA \| Prupe.4G012400 \| \| PpTST1^Gln^ \| PREDICTED: Prunus persica protein LSM12 homolog (LOC18788673), mRNA \| Prupe.1G258500 \| \| PpTST1^Gln^ \| PREDICTED: Prunus persica uncharacterized LOC18775737 (LOC18775737), mRNA \| Prupe.5G220500 \| \| PpTST1^Gln^ \| PREDICTED: Prunus persica uncharacterized LOC18791638 (LOC18791638), transcript variant X4, mRNA \| Prupe.1G480600 \| \| PpTST1^Gln^ \| PREDICTED: Prunus persica uncharacterized LOC18792402 (LOC18792402), mRNA \| Prupe.1G360400 \| \| PpTST1^Gln^ \| PREDICTED: Salmo salar ladderlectin-like (LOC106561635), mRNA \|  \| \| PpTST1^His^ \| PREDICTED: Prunus persica ABC transporter F family member 1 (LOC18773033), mRNA \| Prupe.6G108600 \| \| PpTST1^His^ \| PREDICTED: Prunus persica bifunctional protein FolD 2 (LOC18766873), transcript variant X1, mRNA \| Prupe.8G257500 \| \| PpTST1^His^ \| PREDICTED: Prunus persica probable protein phosphatase 2C 39 (LOC18779143), mRNA \| Prupe.4G177100 \| \| PpTST1^His^ \| PREDICTED: Prunus persica probable signal peptidase complex subunit 2 (LOC18785525), mRNA \| Prupe.2G246200 \| \| PpTST1^His^ \| PREDICTED: Prunus persica protein C2-DOMAIN ABA-RELATED 4 (LOC18777103), transcript variant X2, mRNA \| Prupe.5G233300 \| \| PpTST1^His^ \| PREDICTED: Prunus persica zinc finger CCCH domain-containing protein 11 (LOC18774773), mRNA \| Prupe.6G243000 \| \| PpTST1^His^ \| PREDICTED: Prunus persica zinc finger protein CONSTANS-LIKE 4 (LOC18789515), mRNA \| Prupe.1G398700 \| | | | | | |
|  |  |  |  |  |  |

| \| **Table S6. The TA and TOA values of 100 accessions** \| \| \| \| --- \| --- \| --- \| \| Accessions \| Mean TA (%) \| Mean TOA (g/kg) \| \| Wujiang Bai \| 0.13875 \| 7.526502 \| \| Guizhou Shui Mi \| 0.158299 \| 7.389034 \| \| Xiang Jiao Tao \| 0.164155 \| 6.831847 \| \| Xia Hui 1 \| 0.165 \| 7.703418 \| \| Sa Hua Hong Pan Tao \| 0.16502 \| 7.089064 \| \| Shenzhou Li He Shui Mi \| 0.171222 \| 7.330971 \| \| Rui Guang 3 \| 0.172633 \| 6.866912 \| \| Bai Hua \| 0.174333 \| 7.518315 \| \| Qing Tao \| 0.176422 \| 7.436483 \| \| Hakuho \| 0.181667 \| 7.092221 \| \| Zhong You 5 \| 0.185 \| 7.215671 \| \| Han Lu Mi \| 0.185727 \| 7.011257 \| \| Nunome Wase \| 0.186517 \| 7.516829 \| \| Hua Guang \| 0.195 \| 8.225864 \| \| Matsumori \| 0.1955 \| 8.101364 \| \| Sunago Wase \| 0.2 \| 6.915727 \| \| Jin Feng \| 0.2 \| 7.653899 \| \| Yan Hong \| 0.203217 \| 7.622962 \| \| Chang Sheng Pan Tao \| 0.203291 \| 8.158714 \| \| Zao Huang Pan Tao \| 0.205 \| 7.379595 \| \| Kanoiwa \| 0.205 \| 8.453175 \| \| Yu Lu Pan Tao \| 0.206033 \| 7.998196 \| \| Xingyi Bai Hua Tao \| 0.206063 \| 7.019552 \| \| Shi Tou Tao \| 0.207691 \| 8.716325 \| \| Shiwo Shui Mi \| 0.207778 \| 8.24894 \| \| Shen Zhou Shui Mi \| 0.212138 \| 8.257401 \| \| Hu You 003 \| 0.212733 \| 8.569016 \| \| Hong Ya Zui \| 0.215741 \| 8.174314 \| \| Diao Zhi Bai \| 0.216286 \| 7.880469 \| \| Wuhan Da Hong Pao \| 0.22 \| 8.310898 \| \| Jing Yu \| 0.22045 \| 9.91299 \| \| Yumyeong \| 0.2206 \| 9.786157 \| \| Changling Zao Yu Lu \| 0.223238 \| 9.654479 \| \| Zao Hong Zhu \| 0.224967 \| 8.080597 \| \| Shu Guang \| 0.225 \| 7.15602 \| \| Bai Mang Pan Tao \| 0.225 \| 8.189393 \| \| Li He Pan Tao \| 0.225 \| 9.280012 \| \| Mai Huang Pan Taao \| 0.2291 \| 8.571285 \| \| Zhong You Pan 4 \| 0.245017 \| 8.799934 \| \| Okubo \| 0.251233 \| 8.357694 \| \| Su Hong \| 0.255 \| 8.445899 \| \| Ji Zhui Bai \| 0.255344 \| 8.938788 \| \| Kawanakajima Hakuto \| 0.26 \| 9.344928 \| \| Shangshan Da Yu Lu \| 0.266183 \| 9.125977 \| \| Xiao Bai Tao \| 0.2814 \| 9.868764 \| \| Baisha \| 0.281956 \| 9.938051 \| \| Yang Tao \| 0.282933 \| 9.904327 \| \| Fei Cheng Hong Li 6 \| 0.307223 \| 9.55258 \| \| Babygold 5 \| 0.425 \| 13.72776 \| \| Kashi 1 \| 0.44 \| 14.75946 \| \| Zhang Bai Gan \| 0.465 \| 11.14591 \| \| Yangzhou 3 \| 0.475 \| 12.5793 \| \| Qinling Dong Tao \| 0.490056 \| 14.16052 \| \| Rou Pan Tao \| 0.491 \| 11.79753 \| \| Gaotai 1 \| 0.493333 \| 11.84784 \| \| Fuzalode \| 0.5 \| 14.96107 \| \| Li He Tian Ren \| 0.50857 \| 16.08187 \| \| Cullinan \| 0.51 \| 13.62414 \| \| Tian Li Guang \| 0.512267 \| 12.95184 \| \| Xian Tao \| 0.514497 \| 14.45041 \| \| Kashi 3 \| 0.52 \| 11.41212 \| \| Hong Li Guang \| 0.524137 \| 14.6909 \| \| Chinese Cling \| 0.534611 \| 13.65774 \| \| Lin Huang 1 \| 0.55 \| 13.74077 \| \| NJC108 \| 0.56 \| 12.36807 \| \| Lincheng Tao \| 0.578804 \| 15.78349 \| \| Shui Bai Tao \| 0.58 \| 12.58433 \| \| Huang Nian Hu \| 0.587367 \| 13.23872 \| \| Huang La Tao \| 0.588 \| 12.36899 \| \| NJN76 \| 0.5881 \| 13.88376 \| \| Lian Huang \| 0.595 \| 15.33008 \| \| Texstar \| 0.6185 \| 14.5162 \| \| Wanzhou Suan Tao \| 0.625 \| 14.59989 \| \| Ping Yong Tao \| 0.634128 \| 17.51612 \| \| Tie 4-1 \| 0.634748 \| 18.00098 \| \| Suan Tao \| 0.636656 \| 16.52608 \| \| Wu Da Tao \| 0.659086 \| 15.97011 \| \| May Fire \| 0.669033 \| 15.57838 \| \| Linze Zi Tao \| 0.67185 \| 15.76151 \| \| Kashi 2 \| 0.68 \| 16.62757 \| \| Xiang Tao \| 0.685 \| 14.71643 \| \| Xinjiang Huang Rou \| 0.690286 \| 18.43374 \| \| Maravilha \| 0.695 \| 15.87797 \| \| Flordagold \| 0.7 \| 17.1999 \| \| Anlong Bai Tao \| 0.70674 \| 18.01905 \| \| Bai Li Hu \| 0.726967 \| 17.07152 \| \| Snow Queen \| 0.735317 \| 16.83365 \| \| Shi Tao \| 0.75 \| 17.4145 \| \| NJC77 \| 0.775 \| 18.45164 \| \| Early Red 2 \| 0.796583 \| 18.24979 \| \| Okitsu \| 0.82 \| 20.25276 \| \| Long You Pan Tao \| 0.833725 \| 18.57526 \| \| Hong Tao \| 0.840844 \| 19.07528 \| \| Yuan Chun Bai \| 0.845 \| 19.57866 \| \| Moyu 8 \| 0.850008 \| 22.29188 \| \| Croce del Sud \| 0.861533 \| 19.56149 \| \| Nectaross \| 0.885 \| 19.09888 \| \| Jiu Yang Qing Tao \| 0.994222 \| 22.60642 \| \| Zhou Xing Shan Tao \| 1.208233 \| 27.17465 \| \| Bai Gen Gan Su Tao \| 1.269675 \| 32.19725 \|  \| **Table S7. The TA values and genotypes of 169 accessions from 5 prunus species** \| \| \| \| \| --- \| --- \| --- \| --- \| \| Accessions \| Mean TA (%) \| Genotype \| Species \| \| Linzhou Tianpingshan 1 \| 2.24 \| G/G \| P. davidiana (Carr.) Franch. \| \| Hong Jing Gan Su Tao \| 2.04 \| G/G \| P. kansuensis Rehd. \| \| Li He Guanghetao \| 1.82 \| G/G \| Prunus mira Koehne \| \| Gan Su Tao \| 1.7755 \| G/G \| P. kansuensis Rehd. \| \| Lv Jing Gan Su Tao \| 1.74 \| G/G \| P. kansuensis Rehd. \| \| Bai Gen Gan Su Tao \| 1.269674744 \| G/G \| P. kansuensis Rehd. \| \| Guanghetao \| 1.24 \| G/G \| Prunus mira Koehne \| \| Zhou Xing Shan Tao \| 1.208233333 \| G/G \| P. davidiana (Carr.) Franch. \| \| Da Guo Hei Tao \| 1.187759213 \| G/G \| P. persica (L.) Batsch. \| \| Hong Hua Shan Tao \| 1.045 \| G/G \| P. davidiana (Carr.) Franch. \| \| Aba Guang He Tao \| 1.04 \| G/G \| Prunus mira Koehne \| \| Jiu Yang Qing Tao \| 0.994222222 \| G/G \| P. persica (L.) Batsch. \| \| Zao Chun Tao \| 0.972758661 \| G/G \| P. persica (L.) Batsch. \| \| Suan Li Guang \| 0.93 \| G/G \| P. persica (L.) Batsch. \| \| Ankou Shan Tao \| 0.91 \| G/G \| P. davidiana (Carr.) Franch. \| \| Xinjiang Pan Tao \| 0.9045 \| G/G \| P. ferganensis Kost.et Riab. \| \| Qiu Bai Tao \| 0.892 \| G/G \| P. persica (L.) Batsch. \| \| Nectaross \| 0.885 \| G/G \| P. persica (L.) Batsch. \| \| Croce del Sud \| 0.861533333 \| G/G \| P. persica (L.) Batsch. \| \| Mayglo \| 0.856333333 \| G/G \| P. persica (L.) Batsch. \| \| Moyu 8 \| 0.850008078 \| G/G \| P. persica (L.) Batsch. \| \| Yuan Chun Bai \| 0.845 \| G/G \| P. persica (L.) Batsch. \| \| Hong Tao \| 0.8408441 \| G/G \| P. persica (L.) Batsch. \| \| Long You Pan Tao \| 0.833725 \| G/G \| P. persica (L.) Batsch. \| \| Okitsu \| 0.82 \| G/G \| P. persica (L.) Batsch. \| \| Kashi Huang Rou Li Guang \| 0.799655556 \| G/G \| P. persica (L.) Batsch. \| \| Early Red 2 \| 0.796583333 \| G/G \| P. persica (L.) Batsch. \| \| Regina \| 0.791975 \| G/G \| P. persica (L.) Batsch. \| \| Nectagrand 4 \| 0.79 \| G/G \| P. persica (L.) Batsch. \| \| Hardired \| 0.79 \| G/G \| P. persica (L.) Batsch. \| \| Bai He Tao \| 0.788716667 \| G/G \| P. persica (L.) Batsch. \| \| NJC77 \| 0.775 \| G/G \| P. persica (L.) Batsch. \| \| NJN80 \| 0.756366667 \| G/G \| P. persica (L.) Batsch. \| \| Shi Tao \| 0.75 \| G/G \| P. persica (L.) Batsch. \| \| He Yang You Tao \| 0.745 \| G/G \| P. persica (L.) Batsch. \| \| Cheonhong \| 0.737916667 \| G/G \| P. persica (L.) Batsch. \| \| Snow Queen \| 0.735316667 \| G/G \| P. persica (L.) Batsch. \| \| Tian Jin Shui Mi \| 0.732977329 \| G/G \| P. persica (L.) Batsch. \| \| Bai Li Hu \| 0.726966667 \| G/G \| P. persica (L.) Batsch. \| \| Armking \| 0.72 \| G/G \| P. persica (L.) Batsch. \| \| Bai Nian Hu \| 0.715 \| G/G \| P. persica (L.) Batsch. \| \| Zhang Bai 2 \| 0.712944444 \| G/G \| P. persica (L.) Batsch. \| \| Anlong Bai Tao \| 0.706739967 \| G/G \| P. persica (L.) Batsch. \| \| Flordagold \| 0.7 \| G/G \| P. persica (L.) Batsch. \| \| Maravilha \| 0.695 \| G/G \| P. persica (L.) Batsch. \| \| Xinjiang Huang Rou \| 0.690285856 \| G/G \| P. ferganensis Kost.et Riab. \| \| Xiang Tao \| 0.685 \| G/G \| P. persica (L.) Batsch. \| \| Kashi 2 \| 0.68 \| G/G \| P. ferganensis Kost.et Riab. \| \| Da Jin Dan \| 0.674566667 \| G/G \| P. persica (L.) Batsch. \| \| Linze Zi Tao \| 0.67185 \| G/G \| P. persica (L.) Batsch. \| \| May Fire \| 0.669033333 \| G/G \| P. persica (L.) Batsch. \| \| Wu Da Tao \| 0.659086456 \| G/G \| P. persica (L.) Batsch. \| \| Suan Tao \| 0.636655974 \| G/G \| P. persica (L.) Batsch. \| \| Tie 4-1 \| 0.634748375 \| G/G \| P. persica (L.) Batsch. \| \| Ping Yong Tao \| 0.634127529 \| G/G \| P. persica (L.) Batsch. \| \| Wanzhou Suan Tao \| 0.625 \| G/G \| P. persica (L.) Batsch. \| \| Redhaven \| 0.6235 \| G/G \| P. persica (L.) Batsch. \| \| Tu- 2 \| 0.622525528 \| G/G \| P. persica (L.) Batsch. \| \| Texstar \| 0.6185 \| G/G \| P. persica (L.) Batsch. \| \| Da Li He Huang Rou \| 0.608533333 \| G/G \| P. persica (L.) Batsch. \| \| Lian Huang \| 0.595 \| G/G \| P. persica (L.) Batsch. \| \| Hetian Huang Rou \| 0.594666667 \| G/G \| P. persica (L.) Batsch. \| \| NJN76 \| 0.5881 \| G/G \| P. persica (L.) Batsch. \| \| Huang La Tao \| 0.588 \| G/G \| P. persica (L.) Batsch. \| \| Huang Nian Hu \| 0.587366667 \| G/G \| P. persica (L.) Batsch. \| \| Shui Bai Tao \| 0.58 \| G/G \| P. persica (L.) Batsch. \| \| Lincheng Tao \| 0.578803506 \| G/G \| P. persica (L.) Batsch. \| \| NJC108 \| 0.56 \| G/G \| P. persica (L.) Batsch. \| \| Dicon \| 0.556833333 \| G/G \| P. persica (L.) Batsch. \| \| Lin Huang 1 \| 0.55 \| G/G \| P. persica (L.) Batsch. \| \| Chinese Cling \| 0.534611111 \| G/G \| P. persica (L.) Batsch. \| \| Hong Li Guang \| 0.524136639 \| G/G \| P. persica (L.) Batsch. \| \| Kashi 3 \| 0.52 \| G/G \| P. ferganensis Kost.et Riab. \| \| Flordaking \| 0.5179 \| G/G \| P. persica (L.) Batsch. \| \| Xian Tao \| 0.5144965 \| G/G \| P. persica (L.) Batsch. \| \| Tian Li Guang \| 0.512266667 \| G/G \| P. persica (L.) Batsch. \| \| Cullinan \| 0.51 \| G/G \| P. persica (L.) Batsch. \| \| Li He Tian Ren \| 0.508570006 \| G/G \| P. persica (L.) Batsch. \| \| Fertilia Morettini \| 0.5014 \| G/G \| P. persica (L.) Batsch. \| \| Fuzalode \| 0.5 \| G/G \| P. persica (L.) Batsch. \| \| Huo Lian Jin Dan \| 0.499016667 \| G/G \| P. persica (L.) Batsch. \| \| Gaotai 1 \| 0.493333333 \| G/G \| P. persica (L.) Batsch. \| \| Rou Pan Tao \| 0.491 \| G/G \| P. persica (L.) Batsch. \| \| Qinling Dong Tao \| 0.490055556 \| G/G \| P. persica (L.) Batsch. \| \| Tian Ren Tao \| 0.49 \| G/G \| P. ferganensis Kost.et Riab. \| \| Yangzhou 3 \| 0.475 \| G/G \| P. persica (L.) Batsch. \| \| Zhang Bai Gan \| 0.465 \| G/G \| P. persica (L.) Batsch. \| \| Early Crawford \| 0.456025 \| G/G \| P. persica (L.) Batsch. \| \| Kashi 1 \| 0.44 \| G/G \| P. ferganensis Kost.et Riab. \| \| Babygold 5 \| 0.425 \| G/G \| P. persica (L.) Batsch. \| \| Dawangzhuang Huang Tao \| 0.4077 \| G/G \| P. persica (L.) Batsch. \| \| Wuhan 2 \| 0.39 \| T/G \| P. persica (L.) Batsch. \| \| Guanghetao \| 0.37 \| T/G \| P. kansuensis Rehd. \| \| Qing Mao Zi Bai Hua \| 0.360666667 \| T/T \| P. persica (L.) Batsch. \| \| Hei Bu Dai \| 0.3595 \| T/G \| P. persica (L.) Batsch. \| \| Zhong You Pan 1 \| 0.3425 \| T/G \| P. persica (L.) Batsch. \| \| Qin Guang \| 0.338133333 \| T/G \| P. persica (L.) Batsch. \| \| Fei Cheng Hong Li 6 \| 0.307223325 \| T/G \| P. persica (L.) Batsch. \| \| Mei Gui Hong \| 0.286866667 \| T/G \| P. persica (L.) Batsch. \| \| Yang Tao \| 0.282933333 \| T/G \| P. persica (L.) Batsch. \| \| Baisha \| 0.281956258 \| T/G \| P. persica (L.) Batsch. \| \| Xiao Bai Tao \| 0.2814 \| T/G \| P. persica (L.) Batsch. \| \| Jian Zui Hong Rou \| 0.275 \| T/G \| P. persica (L.) Batsch. \| \| Shangshan Da Yu Lu \| 0.266183333 \| T/G \| P. persica (L.) Batsch. \| \| Jingmen Tao (Mao) \| 0.2641 \| T/G \| P. persica (L.) Batsch. \| \| Kawanakajima Hakuto \| 0.26 \| T/G \| P. persica (L.) Batsch. \| \| Da Hong Pao \| 0.255571339 \| T/G \| P. persica (L.) Batsch. \| \| Ji Zhui Bai \| 0.255343544 \| T/G \| P. persica (L.) Batsch. \| \| Su Hong \| 0.255 \| T/G \| P. persica (L.) Batsch. \| \| Okubo \| 0.251233333 \| T/G \| P. persica (L.) Batsch. \| \| Zhong You Pan 4 \| 0.245016667 \| T/G \| P. persica (L.) Batsch. \| \| Da Hong Pao \| 0.238466667 \| T/G \| P. persica (L.) Batsch. \| \| Huang Jin Pan Tao \| 0.235 \| T/G \| P. persica (L.) Batsch. \| \| Mai Huang Pan Taao \| 0.2291 \| T/G \| P. persica (L.) Batsch. \| \| Li He Pan Tao \| 0.225 \| T/G \| P. persica (L.) Batsch. \| \| Bai Mang Pan Tao \| 0.225 \| T/G \| P. persica (L.) Batsch. \| \| Shu Guang \| 0.225 \| T/G \| P. persica (L.) Batsch. \| \| Zao Hong Zhu \| 0.224966667 \| T/G \| P. persica (L.) Batsch. \| \| Guizhou Huang Jin Mi \| 0.223416667 \| T/G \| P. persica (L.) Batsch. \| \| Changling Zao Yu Lu \| 0.223238339 \| T/T \| P. persica (L.) Batsch. \| \| Wangmo Xiao Mi Tao \| 0.221666667 \| T/G \| P. persica (L.) Batsch. \| \| Yumyeong \| 0.2206 \| T/G \| P. persica (L.) Batsch. \| \| Jing Yu \| 0.22045 \| T/G \| P. persica (L.) Batsch. \| \| Hong Shan Hu \| 0.22 \| T/G \| P. persica (L.) Batsch. \| \| Wei Jian Hong Rou \| 0.22 \| T/G \| P. persica (L.) Batsch. \| \| Wuhan Da Hong Pao \| 0.22 \| T/G \| P. persica (L.) Batsch. \| \| Diao Zhi Bai \| 0.216286138 \| T/G \| P. persica (L.) Batsch. \| \| Hong Ya Zui \| 0.215741192 \| T/G \| P. persica (L.) Batsch. \| \| Da Tian Tao \| 0.215016667 \| T/G \| P. persica (L.) Batsch. \| \| Hu You 003 \| 0.212733333 \| T/T \| P. persica (L.) Batsch. \| \| Shen Zhou Shui Mi \| 0.212138139 \| T/G \| P. persica (L.) Batsch. \| \| Shiwo Shui Mi \| 0.207777778 \| T/G \| P. persica (L.) Batsch. \| \| Shi Tou Tao \| 0.207691067 \| T/G \| P. persica (L.) Batsch. \| \| Xingyi Bai Hua Tao \| 0.206063197 \| T/G \| P. persica (L.) Batsch. \| \| Yu Lu Pan Tao \| 0.206033333 \| T/G \| P. persica (L.) Batsch. \| \| Kanoiwa \| 0.205 \| T/G \| P. persica (L.) Batsch. \| \| Zao Huang Pan Tao \| 0.205 \| T/G \| P. persica (L.) Batsch. \| \| Chang Sheng Pan Tao \| 0.203291042 \| T/T \| P. persica (L.) Batsch. \| \| Yan Hong \| 0.203216667 \| T/G \| P. persica (L.) Batsch. \| \| Sunago Wase \| 0.2 \| T/G \| P. persica (L.) Batsch. \| \| Wan Bai Mi \| 0.2 \| T/G \| P. persica (L.) Batsch. \| \| Jin Feng \| 0.2 \| T/T \| P. persica (L.) Batsch. \| \| Matsumori \| 0.1955 \| T/T \| P. persica (L.) Batsch. \| \| Hua Guang \| 0.195 \| T/G \| P. persica (L.) Batsch. \| \| Ju Shan Tong Tao \| 0.195 \| T/G \| P. persica (L.) Batsch. \| \| Hu You 004 \| 0.192733333 \| T/G \| P. persica (L.) Batsch. \| \| Nunome Wase \| 0.186516667 \| T/G \| P. persica (L.) Batsch. \| \| Han Lu Mi \| 0.185727094 \| T/G \| P. persica (L.) Batsch. \| \| Hua Yu Lu \| 0.18525 \| T/G \| P. persica (L.) Batsch. \| \| Zhong You 5 \| 0.185 \| T/G \| P. persica (L.) Batsch. \| \| Xiao Hong Hua \| 0.184966667 \| T/G \| P. persica (L.) Batsch. \| \| Hakuho \| 0.181666667 \| T/G \| P. persica (L.) Batsch. \| \| Sha Hong Tao \| 0.17875 \| T/G \| P. persica (L.) Batsch. \| \| Da Zhao Huang Tao \| 0.177797398 \| T/G \| P. persica (L.) Batsch. \| \| Qing Tao \| 0.176421522 \| T/G \| P. persica (L.) Batsch. \| \| Bai Hua \| 0.174333333 \| T/G \| P. persica (L.) Batsch. \| \| Rui Guang 3 \| 0.172633333 \| T/G \| P. persica (L.) Batsch. \| \| Shenzhou Li He Shui Mi \| 0.171221656 \| T/G \| P. persica (L.) Batsch. \| \| Sa Hua Hong Pan Tao \| 0.165020396 \| T/G \| P. persica (L.) Batsch. \| \| Xia Hui 1 \| 0.165 \| T/G \| P. persica (L.) Batsch. \| \| Xiang Jiao Tao \| 0.164154922 \| T/G \| P. persica (L.) Batsch. \| \| Zhong You 5 Zao Shu Ya Bian \| 0.1637 \| T/G \| P. persica (L.) Batsch. \| \| Guizhou Shui Mi \| 0.158299433 \| T/G \| P. persica (L.) Batsch. \| \| Huo Zhu \| 0.14375 \| T/G \| P. persica (L.) Batsch. \| \| Fei Cheng Bai Li 10 \| 0.142932283 \| T/G \| P. persica (L.) Batsch. \| \| Wujiang Bai \| 0.13875 \| T/G \| P. persica (L.) Batsch. \| \| Gucheng Chun Lei \| 0.128116667 \| T/G \| P. persica (L.) Batsch. \| \| Er Jie Bai \| 0.117249517 \| T/G \| P. persica (L.) Batsch. \| \| Fei Cheng Bai Li 17 \| 0.111944746 \| T/T \| P. persica (L.) Batsch. \| | | | |
| --- | --- | --- | --- | --- | --- | --- | --- | --- | --- | --- | --- | --- | --- | --- | --- | --- | --- | --- | --- | --- | --- | --- | --- | --- | --- | --- | --- | --- | --- | --- | --- | --- | --- | --- | --- | --- | --- | --- | --- | --- | --- | --- | --- | --- | --- | --- | --- | --- | --- | --- | --- | --- | --- | --- | --- | --- | --- | --- | --- | --- | --- | --- | --- | --- | --- | --- | --- | --- | --- | --- | --- | --- | --- | --- | --- | --- | --- | --- | --- | --- | --- | --- | --- | --- | --- | --- | --- | --- | --- | --- | --- | --- | --- | --- | --- | --- | --- | --- | --- | --- | --- | --- | --- | --- | --- | --- | --- | --- | --- | --- | --- | --- | --- | --- | --- | --- | --- | --- | --- | --- | --- | --- | --- | --- | --- | --- | --- | --- | --- | --- | --- | --- | --- | --- | --- | --- | --- | --- | --- | --- | --- | --- | --- | --- | --- | --- | --- | --- | --- | --- | --- | --- | --- | --- | --- | --- | --- | --- | --- | --- | --- | --- | --- | --- | --- | --- | --- | --- | --- | --- | --- | --- | --- | --- | --- | --- | --- | --- | --- | --- | --- | --- | --- | --- | --- | --- | --- | --- | --- | --- | --- | --- | --- | --- | --- | --- | --- | --- | --- | --- | --- | --- | --- | --- | --- | --- | --- | --- | --- | --- | --- | --- | --- | --- | --- | --- | --- | --- | --- | --- | --- | --- | --- | --- | --- | --- | --- | --- | --- | --- | --- | --- | --- | --- | --- | --- | --- | --- | --- | --- | --- | --- | --- | --- | --- | --- | --- | --- | --- | --- | --- | --- | --- | --- | --- | --- | --- | --- | --- | --- | --- | --- | --- | --- | --- | --- | --- | --- | --- | --- | --- | --- | --- | --- | --- | --- | --- | --- | --- | --- | --- | --- | --- | --- | --- | --- | --- | --- | --- | --- | --- | --- | --- | --- | --- | --- | --- | --- | --- | --- | --- | --- | --- | --- | --- | --- | --- | --- | --- | --- | --- | --- | --- | --- | --- | --- | --- | --- | --- | --- | --- | --- | --- | --- | --- | --- | --- | --- | --- | --- | --- | --- | --- | --- | --- | --- | --- | --- | --- | --- | --- | --- | --- | --- | --- | --- | --- | --- | --- | --- | --- | --- | --- | --- | --- | --- | --- | --- | --- | --- | --- | --- | --- | --- | --- | --- | --- | --- | --- | --- | --- | --- | --- | --- | --- | --- | --- | --- | --- | --- | --- | --- | --- | --- | --- | --- | --- | --- | --- | --- | --- | --- | --- | --- | --- | --- | --- | --- | --- | --- | --- | --- | --- | --- | --- | --- | --- | --- | --- | --- | --- | --- | --- | --- | --- | --- | --- | --- | --- | --- | --- | --- | --- | --- | --- | --- | --- | --- | --- | --- | --- | --- | --- | --- | --- | --- | --- | --- | --- | --- | --- | --- | --- | --- | --- | --- | --- | --- | --- | --- | --- | --- | --- | --- | --- | --- | --- | --- | --- | --- | --- | --- | --- | --- | --- | --- | --- | --- | --- | --- | --- | --- | --- | --- | --- | --- | --- | --- | --- | --- | --- | --- | --- | --- | --- | --- | --- | --- | --- | --- | --- | --- | --- | --- | --- | --- | --- | --- | --- | --- | --- | --- | --- | --- | --- | --- | --- | --- | --- | --- | --- | --- | --- | --- | --- | --- | --- | --- | --- | --- | --- | --- | --- | --- | --- | --- | --- | --- | --- | --- | --- | --- | --- | --- | --- | --- | --- | --- | --- | --- | --- | --- | --- | --- | --- | --- | --- | --- | --- | --- | --- | --- | --- | --- | --- | --- | --- | --- | --- | --- | --- | --- | --- | --- | --- | --- | --- | --- | --- | --- | --- | --- | --- | --- | --- | --- | --- | --- | --- | --- | --- | --- | --- | --- | --- | --- | --- | --- | --- | --- | --- | --- | --- | --- | --- | --- | --- | --- | --- | --- | --- | --- | --- | --- | --- | --- | --- | --- | --- | --- | --- | --- | --- | --- | --- | --- | --- | --- | --- | --- | --- | --- | --- | --- | --- | --- | --- | --- | --- | --- | --- | --- | --- | --- | --- | --- | --- | --- | --- | --- | --- | --- | --- | --- | --- | --- | --- | --- | --- | --- | --- | --- | --- | --- | --- | --- | --- | --- | --- | --- | --- | --- | --- | --- | --- | --- | --- | --- | --- | --- | --- | --- | --- | --- | --- | --- | --- | --- | --- | --- | --- | --- | --- | --- | --- | --- | --- | --- | --- | --- | --- | --- | --- | --- | --- | --- | --- | --- | --- | --- | --- | --- | --- | --- | --- | --- | --- | --- | --- | --- | --- | --- | --- | --- | --- | --- | --- | --- | --- | --- | --- | --- | --- | --- | --- | --- | --- | --- | --- | --- | --- | --- | --- | --- | --- | --- | --- | --- | --- | --- | --- | --- | --- | --- | --- | --- | --- | --- | --- | --- | --- | --- | --- | --- | --- | --- | --- | --- | --- | --- | --- | --- | --- | --- | --- | --- | --- | --- | --- | --- | --- | --- | --- | --- | --- | --- | --- | --- | --- | --- | --- | --- | --- | --- | --- | --- | --- | --- | --- | --- | --- | --- | --- | --- | --- | --- | --- | --- | --- | --- | --- | --- | --- | --- | --- | --- | --- | --- | --- | --- | --- | --- | --- | --- | --- | --- | --- | --- | --- | --- | --- | --- | --- | --- | --- | --- | --- | --- | --- | --- | --- | --- | --- | --- | --- | --- | --- | --- | --- | --- | --- | --- | --- | --- | --- | --- | --- | --- | --- | --- | --- | --- | --- | --- | --- | --- | --- | --- | --- | --- | --- | --- | --- | --- | --- | --- | --- | --- | --- | --- | --- | --- | --- | --- | --- | --- | --- | --- | --- | --- | --- | --- | --- | --- | --- | --- | --- | --- | --- | --- | --- | --- | --- | --- | --- | --- | --- | --- | --- | --- | --- | --- | --- | --- | --- | --- | --- | --- | --- | --- | --- | --- | --- | --- | --- | --- | --- | --- | --- | --- | --- | --- | --- | --- | --- | --- | --- | --- | --- | --- | --- | --- | --- | --- | --- | --- | --- | --- | --- | --- | --- | --- | --- | --- | --- | --- | --- | --- | --- | --- | --- | --- | --- | --- | --- | --- | --- | --- | --- | --- | --- | --- | --- | --- | --- | --- | --- | --- | --- | --- | --- | --- | --- | --- | --- | --- | --- | --- | --- | --- | --- | --- | --- | --- | --- | --- | --- | --- | --- | --- | --- | --- | --- |
| \| **Table S8. Genotypes of 480 accessions from wild, landrace and improved groups. Genotypes with yellow background do not match the phenotypes. Genotypes with red color were corrected by Sanger sequencing** \| \| \| \| --- \| --- \| --- \| \| \| **Accessions** \| **Groups** \| **Genotype** \| \| 2010 Tibet 15 \| wild \| G/G \| \| 2010 Tibet 17 \| wild \| G/G \| \| 2010 Tibet 26 \| wild \| G/G \| \| 2010 Tibet 28 \| wild \| G/G \| \| 2010 Tibet 29 \| wild \| G/G \| \| 2010 Tibet 32 \| wild \| G/G \| \| 2010 Tibet 54 \| wild \| G/G \| \| 2010 Tibet 55 \| wild \| G/G \| \| 2010 Tibet 57 \| wild \| G/G \| \| 2010-138 \| wild \| G/G \| \| 2010-138b \| wild \| G/G \| \| Aba Guang He Tao \| wild \| G/G \| \| Bai Gen Gansu Tao 1 \| wild \| G/G \| \| Bai Hua Shan Bi Tao \| wild \| G/G \| \| Bai Hua Shan Tao \| wild \| G/G \| \| Basu 1 \| wild \| G/G \| \| Daze 1 \| wild \| G/G \| \| Daze 2 \| wild \| G/G \| \| Hong Gen Gansu Tao 1 \| wild \| G/G \| \| Hong Hua Shan Tao \| wild \| G/G \| \| Hong Rou Guang He Tao \| wild \| ./. \| \| Julong 11 \| wild \| G/G \| \| Julong 12 \| wild \| G/G \| \| Julong 13 \| wild \| G/G \| \| Julong 15 \| wild \| G/G \| \| Julong 16 \| wild \| G/G \| \| Lanong 1 \| wild \| G/G \| \| Lanong 2 \| wild \| G/G \| \| Lanong 3 \| wild \| ./. \| \| Lawu 1 \| wild \| G/G \| \| Lawu 2 \| wild \| G/G \| \| Lawu 3 \| wild \| G/G \| \| Lawu 3-1 \| wild \| G/G \| \| Lawu 4 \| wild \| G/G \| \| Pabangka 1 \| wild \| G/G \| \| Pabangka 2 \| wild \| G/G \| \| Paizhen 1 \| wild \| G/G \| \| Paizhen 2 \| wild \| G/G \| \| Paizhen 3 \| wild \| G/G \| \| Paizhen 4 \| wild \| G/G \| \| Rikaze Guang He Tao \| wild \| G/G \| \| Runa 1 \| wild \| G/G \| \| Shan Gan Shan Tao \| wild \| G/G \| \| Shannan Tibet \| wild \| G/G \| \| Tongmai 1 \| wild \| G/G \| \| Tongmai 2 \| wild \| G/G \| \| Yan Tian Jin Qu Zao Hua Tao \| wild \| G/G \| \| Yuxu 1 \| wild \| ./. \| \| Yuxu 2 \| wild \| G/G \| \| Yuxu 3 \| wild \| G/G \| \| Yuxu 4 \| wild \| G/G \| \| Zhou Xing Shan Tao \| wild \| ./. \| \| Anlong Bai Tao \| Landrace \| G/G \| \| Bai He Tao \| Landrace \| G/G \| \| Bai Hua \| Landrace \| ./. \| \| Bai Li Hu \| Landrace \| G/G \| \| Bai Mang Pan Tao \| Landrace \| T/G \| \| Bai Nian He \| Landrace \| ./. \| \| Bai Nian Hu \| Landrace \| G/G \| \| Bai Sha \| Landrace \| T/G \| \| Bi Nan I \| Landrace \| G/G \| \| Chang Sheng Pan Tao \| Landrace \| T/T \| \| Changling Zao Yu Lu \| Landrace \| T/T \| \| Chinese Cling \| Landrace \| G/G \| \| Da Guo Hei Tao \| Landrace \| G/G \| \| Da Hong Pao \| Landrace \| T/G \| \| Da Hong Tao \| Landrace \| T/T \| \| Da Jin Dan \| Landrace \| G/G \| \| Da Li He Huang Rou \| Landrace \| G/G \| \| Da Tian Tao \| Landrace \| T/G \| \| Da Xue Tao \| Landrace \| T/T \| \| Damiao Qing Pi Tao \| Landrace \| G/G \| \| Diao Zhi Bai \| Landrace \| T/G \| \| Dingjiaba Hong Li Guang Tao \| Landrace \| G/G \| \| Dingjiaba Qing Pi Tao \| Landrace \| G/G \| \| Dujiadun Wan Ge Da Tao \| Landrace \| G/G \| \| Dunhuang Dong Tao \| Landrace \| G/G \| \| Er Jie Bai \| Landrace \| T/G \| \| Er Zao Tao \| Landrace \| G/G \| \| Feicheng Bai Li 10 \| Landrace \| T/G \| \| Feicheng Bai Li 17 \| Landrace \| T/T \| \| Feicheng Hong Li 6 \| Landrace \| T/G \| \| Feijing Tao \| Landrace \| G/G \| \| Fen Shou Xing \| Landrace \| G/G \| \| Fenghua Pan Tao \| Landrace \| T/G \| \| Gaotai 1 \| Landrace \| G/G \| \| Ge Gu \| Landrace \| ./. \| \| Gua Tao \| Landrace \| G/G \| \| Gucheng Da Hong Pao \| Landrace \| T/G \| \| Guizhou Shui Mi \| Landrace \| T/G \| \| Han Lu Mi \| Landrace \| T/G \| \| Hetian Huang Rou \| Landrace \| G/G \| \| Hetian Wan You Tao \| Landrace \| G/G \| \| Hong Chui Zhi \| Landrace \| G/G \| \| Hong Hua Bi Tao \| Landrace \| G/G \| \| Hong Li Guang \| Landrace \| G/G \| \| Hong Shou Xing \| Landrace \| G/G \| \| Hong Tao \| Landrace \| G/G \| \| Hong Ya Zui \| Landrace \| T/G \| \| Hong Ye Tao \| Landrace \| G/G \| \| Hongguang Wan Li Guang Tao \| Landrace \| G/G \| \| Hu Jing Mi Lu \| Landrace \| T/G \| \| Huang Jin Pan Tao \| Landrace \| T/G \| \| Huang La Tao \| Landrace \| G/G \| \| Huang Li Guang \| Landrace \| G/G \| \| Huang Nian Hu \| Landrace \| G/G \| \| Huang Yan \| Landrace \| G/G \| \| Huangguang Da Li Guang Tao \| Landrace \| G/G \| \| Hunchun Tao Shi Sheng \| Landrace \| G/G \| \| Hunchun Tao Shi Sheng \| Landrace \| G/G \| \| Huo Lian Jin Dan \| Landrace \| G/G \| \| Huo Zhu \| Landrace \| T/G \| \| Ji Zui Bai \| Landrace \| T/G \| \| Jian Zui Hong Rou \| Landrace \| T/G \| \| Jiangcun 1 \| Landrace \| G/G \| \| Jiangcun 4 \| Landrace \| G/G \| \| Jiaqing Pan Tao \| Landrace \| T/G \| \| Jilin 8501 \| Landrace \| G/G \| \| Jilin 8601 \| Landrace \| G/G \| \| Jilin 8701 \| Landrace \| G/G \| \| Jilin 8801 \| Landrace \| G/G \| \| Jilin 8903 \| Landrace \| ./. \| \| Jingmen Tao \| Landrace \| T/G \| \| Jinta Hong Guang \| Landrace \| G/G \| \| Jinta You Pan Tao \| Landrace \| G/G \| \| Jiuyang Qing Tao \| Landrace \| G/G \| \| Ju Hua Tao \| Landrace \| G/G \| \| Jushan Tong Tao \| Landrace \| T/G \| \| Kashi 1 \| Landrace \| G/G \| \| Kashi 2 \| Landrace \| G/G \| \| Kashi 3 \| Landrace \| G/G \| \| Kashi 4 \| Landrace \| G/G \| \| Kashi Huang Rou Li Guang \| Landrace \| G/G \| \| Kuanxi Tao \| Landrace \| G/G \| \| Langanxiang 1 \| Landrace \| G/G \| \| Langanxiang 2 \| Landrace \| G/G \| \| Langanxiang 3 \| Landrace \| G/G \| \| Li He Pan Tao \| Landrace \| T/G \| \| Li He Tian Ren \| Landrace \| G/G \| \| Lianhuashan 1 \| Landrace \| G/G \| \| Lianhuashan 2 \| Landrace \| G/G \| \| Lianhuashan 3 \| Landrace \| G/G \| \| Licheng Tao \| Landrace \| G/G \| \| Lin Bai 10 \| Landrace \| ./. \| \| Lin Bai 3 \| Landrace \| G/G \| \| Lin Huang 1 \| Landrace \| G/G \| \| Linze Zi Tao \| Landrace \| G/G \| \| Liu Yue Bai \| Landrace \| T/G \| \| Liu Yue Kong \| Landrace \| T/G \| \| Long 1-2-3 \| Landrace \| G/G \| \| Long 1-2-4 \| Landrace \| G/G \| \| Long 1-2-6 \| Landrace \| G/G \| \| Long 2-4-6 \| Landrace \| G/G \| \| Longhua Shui Mi \| Landrace \| G/G \| \| Longsheng Da Wan Tao \| Landrace \| G/G \| \| Longsheng Pan Tao \| Landrace \| ./. \| \| Longsheng Xin Jiang Tao \| Landrace \| G/G \| \| Lulin Shui Mi \| Landrace \| ./. \| \| Mi Yang Shan \| Landrace \| G/G \| \| Mo Yu 8 \| Landrace \| G/G \| \| Nan Fang Hong Hua Zao \| Landrace \| G/G \| \| Nan Fang Zao Shu Tao \| Landrace \| T/T \| \| Nan Shan 1 \| Landrace \| G/G \| \| Nanshan Tian Tao \| Landrace \| T/G \| \| Ping Bei Zi \| Landrace \| T/G \| \| Ping Ding Qiu Tao \| Landrace \| T/G \| \| Pingyong Tao \| Landrace \| G/G \| \| Qi Tao \| Landrace \| G/G \| \| Qing Mao Zi Bai Hua \| Landrace \| T/T \| \| Qing Si \| Landrace \| G/G \| \| Qing Tao \| Landrace \| T/G \| \| Qingling Dong Tao \| Landrace \| G/G \| \| Qingzhou Bai Pi Mi Tao \| Landrace \| T/T \| \| Qingzhou Hong Pi Mi Tao \| Landrace \| T/T \| \| Qinjiawan Zao Tao \| Landrace \| G/G \| \| Qiu Bai Tao \| Landrace \| G/G \| \| Rou Pan Tao \| Landrace \| G/G \| \| Sa Hong Tao \| Landrace \| T/G \| \| Sa Hua Hong Pan Tao \| Landrace \| T/G \| \| Shandong Si Yue Ban \| Landrace \| T/G \| \| Shang Shan Da Yu Lu \| Landrace \| T/G \| \| Shenyang 1 \| Landrace \| ./. \| \| Shenyang 2 \| Landrace \| G/G \| \| Shenyang 3 \| Landrace \| G/G \| \| Shenyang 4 \| Landrace \| G/G \| \| Shenyang 5 \| Landrace \| G/G \| \| Shenyang Wu Yue Xian \| Landrace \| G/G \| \| Shenzhou Bai Mi \| Landrace \| T/G \| \| Shenzhou Li He Shui Mi \| Landrace \| T/G \| \| Shenzhou Shui Mi \| Landrace \| T/G \| \| Shi Tao \| Landrace \| G/G \| \| Shi Wo Shui Mi \| Landrace \| ./. \| \| Shui Bai Tao \| Landrace \| G/G \| \| Su Hong \| Landrace \| T/G \| \| Suan Li Guang \| Landrace \| G/G \| \| Suan Tao \| Landrace \| G/G \| \| Tai Bai \| Landrace \| G/G \| \| Taiyuan Shui Mi \| Landrace \| T/G \| \| Tan Chun \| Landrace \| G/G \| \| Tayuan Nian He Tao \| Landrace \| G/G \| \| Tian Li Guang \| Landrace \| G/G \| \| Tian Ren Tao \| Landrace \| G/G \| \| Tianjin Shui Mi \| Landrace \| G/G \| \| Tie 4-1 \| Landrace \| G/G \| \| Tu-2 \| Landrace \| G/G \| \| Tugou 1 \| Landrace \| G/G \| \| Tupulukexiang 1 \| Landrace \| G/G \| \| Tupulukexiang 3 \| Landrace \| G/G \| \| Wan Pan Tao \| Landrace \| T/G \| \| Wangmo Xiao Mi Tao \| Landrace \| T/G \| \| Wanzhou Suan Tao \| Landrace \| G/G \| \| Wei Jian Hong Rou \| Landrace \| T/G \| \| Wenzhou Shui Mi \| Landrace \| G/G \| \| Wu Bao Tao \| Landrace \| G/G \| \| Wu Hei Ji Rou Tao \| Landrace \| G/G \| \| Wu Yue Bai \| Landrace \| ./. \| \| Wu yue Xian \| Landrace \| T/G \| \| Wu Yue Xian Biao Gan \| Landrace \| T/G \| \| Wuda Tao \| Landrace \| G/G \| \| Wuhan 2 \| Landrace \| T/G \| \| Wuhan Da Hong Pao \| Landrace \| T/G \| \| Wujiang Bai \| Landrace \| T/G \| \| Wutongshan 1 \| Landrace \| G/G \| \| Xiamiao 1 \| Landrace \| G/G \| \| Xian Tao \| Landrace \| G/G \| \| Xiang Tao \| Landrace \| G/G \| \| Xiao Bai Tao \| Landrace \| T/G \| \| Xiao Hong Hua \| Landrace \| T/G \| \| Xiao Jin Dan \| Landrace \| T/T \| \| Xidong Xiao Li Guang Tao \| Landrace \| G/G \| \| Xijiao 2 \| Landrace \| G/G \| \| Xijiao 3 \| Landrace \| G/G \| \| Xin Jiang Ye You Tao \| Landrace \| G/G \| \| Xingyi Bai Hua Tao \| Landrace \| T/G \| \| Xingyi Wu Yue Tao \| Landrace \| T/G \| \| Xinjiang Huang Rou \| Landrace \| G/G \| \| Xinjiang Pan Tao \| Landrace \| G/G \| \| Xizhuang 1 \| Landrace \| G/G \| \| Yang Tao \| Landrace \| T/G \| \| Yangquan Rou Tao \| Landrace \| T/T \| \| Yexian Dong Tao \| Landrace \| G/G \| \| Yexiang Huang Rou Tao \| Landrace \| G/G \| \| Yi Hong Shui Mi \| Landrace \| T/G \| \| Yi Xian Hong \| Landrace \| T/G \| \| Yili Xian Huang Rou \| Landrace \| G/G \| \| Ying Ge Tao \| Landrace \| ./. \| \| Ying Zui Huang Tao \| Landrace \| G/G \| \| Yu Lu \| Landrace \| T/T \| \| Yu Lu Pan Tao \| Landrace \| T/G \| \| Yuan Chun Bai \| Landrace \| G/G \| \| Yuan Yang Chui Zhi \| Landrace \| G/G \| \| Yueya Da Ye Hong Li Guang \| Landrace \| G/G \| \| Yueya Hong Li Guang \| Landrace \| G/G \| \| Yueya Wan Ge Da Tao \| Landrace \| G/G \| \| Zao Huang Jin \| Landrace \| T/G \| \| Zao Shanghai Shui Mi \| Landrace \| G/G \| \| Zao Shu Huang Gan \| Landrace \| G/G \| \| Zepu 4 \| Landrace \| G/G \| \| Zepu 6 \| Landrace \| G/G \| \| Zepu 8 \| Landrace \| G/G \| \| Zhang Bai 2 \| Landrace \| G/G \| \| Zhang Bai 5 \| Landrace \| G/G \| \| Zhang Bai Gan \| Landrace \| G/G \| \| Zhong Hua Shou Tao \| Landrace \| T/G \| \| Zhu Fen Chui Zhi \| Landrace \| G/G \| \| 124 Pan Tao \| Improved cultivar \| T/G \| \| 21 shiji \| Improved cultivar \| T/T \| \| Abehakuto \| Improved cultivar \| ./. \| \| Amsden \| Improved cultivar \| G/G \| \| An Nong Shui Mi \| Improved cultivar \| T/G \| \| Arctic Blaze \| Improved cultivar \| T/G \| \| Arctic Star \| Improved cultivar \| ./. \| \| Armking \| Improved cultivar \| G/G \| \| Asama Hakuto \| Improved cultivar \| T/T \| \| Autumn prince \| Improved cultivar \| G/G \| \| Azumo \| Improved cultivar \| G/G \| \| Babygold 5 \| Improved cultivar \| G/G \| \| Babygold 6 \| Improved cultivar \| G/G \| \| Bai Xiang Lu \| Improved cultivar \| G/G \| \| Bailey \| Improved cultivar \| G/G \| \| Bei Nong 2 \| Improved cultivar \| T/G \| \| Bei Nong Zao Shu \| Improved cultivar \| G/G \| \| Benishimizi \| Improved cultivar \| T/G \| \| Blaze prince \| Improved cultivar \| G/G \| \| Cheng Xiang \| Improved cultivar \| G/G \| \| Cheng Yan \| Improved cultivar \| G/G \| \| Cheonhong \| Improved cultivar \| G/G \| \| Chi Yuan Mi \| Improved cultivar \| T/T \| \| Chun Lei \| Improved cultivar \| T/G \| \| Chun Mi \| Improved cultivar \| T/G \| \| Croce Decsus \| Improved cultivar \| G/G \| \| Croce Del Sud \| Improved cultivar \| G/G \| \| Cullinan \| Improved cultivar \| G/G \| \| Da Bai Suan \| Improved cultivar \| G/G \| \| Dalian 12-28 \| Improved cultivar \| G/G \| \| Dalian 22-8 \| Improved cultivar \| G/G \| \| Dalian 4-35 \| Improved cultivar \| ./. \| \| Dan Mo \| Improved cultivar \| T/G \| \| Denjiulo \| Improved cultivar \| T/G \| \| Dicon \| Improved cultivar \| G/G \| \| Early red 2 \| Improved cultivar \| G/G \| \| Early Yumyeong \| Improved cultivar \| T/G \| \| Early Zrawford \| Improved cultivar \| G/G \| \| Fairlane \| Improved cultivar \| G/G \| \| Fantasia \| Improved cultivar \| G/G \| \| Favolate 2 \| Improved cultivar \| G/G \| \| Fay Elbert \| Improved cultivar \| G/G \| \| Feng Guan 2 \| Improved cultivar \| G/G \| \| Feng Huang \| Improved cultivar \| G/G \| \| Fertilia Morettimi \| Improved cultivar \| G/G \| \| Flame prince \| Improved cultivar \| G/G \| \| Flavortop \| Improved cultivar \| G/G \| \| Flordagold \| Improved cultivar \| G/G \| \| Flordaking \| Improved cultivar \| G/G \| \| Fortuna \| Improved cultivar \| G/G \| \| Fuzalode \| Improved cultivar \| G/G \| \| Gan Xuan 4 \| Improved cultivar \| T/G \| \| Globehaven \| Improved cultivar \| G/G \| \| Hakuho \| Improved cultivar \| T/G \| \| Hakuto \| Improved cultivar \| T/G \| \| Halfold \| Improved cultivar \| ./. \| \| Hang Zhou Zao Shui Mi \| Improved cultivar \| G/G \| \| Harbrite \| Improved cultivar \| G/G \| \| Hardride \| Improved cultivar \| G/G \| \| Harken \| Improved cultivar \| G/G \| \| Harmony \| Improved cultivar \| G/G \| \| Harrow blood \| Improved cultivar \| G/G \| \| Havis \| Improved cultivar \| G/G \| \| Hong Gan Lu \| Improved cultivar \| T/G \| \| Hong Shan Hu \| Improved cultivar \| T/G \| \| Hu You 003 \| Improved cultivar \| T/T \| \| Hu You 004 \| Improved cultivar \| T/G \| \| Hua Guang \| Improved cultivar \| T/G \| \| Huang Jin Mei Li \| Improved cultivar \| T/G \| \| Hui Yun Lu \| Improved cultivar \| T/G \| \| Improved Flavor Crest \| Improved cultivar \| G/G \| \| Jie Tu Bai \| Improved cultivar \| T/G \| \| Jin Xiu \| Improved cultivar \| T/G \| \| Jing Chun \| Improved cultivar \| T/G \| \| Jing Feng \| Improved cultivar \| T/T \| \| Jing Mi \| Improved cultivar \| T/G \| \| Jing Yan \| Improved cultivar \| T/G \| \| Jing Yu \| Improved cultivar \| T/G \| \| June gold \| Improved cultivar \| G/G \| \| Kanoiwa \| Improved cultivar \| T/G \| \| Kanto 14 \| Improved cultivar \| G/G \| \| Kanto 5 \| Improved cultivar \| G/G \| \| Kawanakajima Hakuto \| Improved cultivar \| T/G \| \| Kouyou Hakuto \| Improved cultivar \| T/G \| \| Kugetsu \| Improved cultivar \| T/G \| \| Kurakato Wase \| Improved cultivar \| T/G \| \| Lai Shan Mi \| Improved cultivar \| T/T \| \| Legrand \| Improved cultivar \| G/G \| \| Li07-1-13 \| Improved cultivar \| T/G \| \| Lian Huang \| Improved cultivar \| G/G \| \| Long You Pan Tao \| Improved cultivar \| G/G \| \| Lu Xiang \| Improved cultivar \| G/G \| \| Mai Huang Pan Tao \| Improved cultivar \| G/G \| \| Mai Xiang \| Improved cultivar \| G/G \| \| Man Tian Hong \| Improved cultivar \| T/G \| \| Maravilha \| Improved cultivar \| T/G \| \| Maria Laura \| Improved cultivar \| G/G \| \| Maria Serena \| Improved cultivar \| G/G \| \| Matsumori \| Improved cultivar \| T/T \| \| May Fire \| Improved cultivar \| G/G \| \| May Grand \| Improved cultivar \| G/G \| \| Mayglo \| Improved cultivar \| G/G \| \| Mei Gui Hong \| Improved cultivar \| T/G \| \| Nan Fang Jin Mi \| Improved cultivar \| T/G \| \| Nectagrand \| Improved cultivar \| G/G \| \| Nectagrand 1 \| Improved cultivar \| G/G \| \| Nectagrand 2 \| Improved cultivar \| G/G \| \| Nectagrand 7 \| Improved cultivar \| G/G \| \| Nectaross \| Improved cultivar \| G/G \| \| Nemaguard \| Improved cultivar \| G/G \| \| NJC106 \| Improved cultivar \| G/G \| \| NJC108 \| Improved cultivar \| G/G \| \| NJC113 \| Improved cultivar \| G/G \| \| NJC20 \| Improved cultivar \| G/G \| \| NJC48 \| Improved cultivar \| G/G \| \| NJC77 \| Improved cultivar \| G/G \| \| NJC83 \| Improved cultivar \| G/G \| \| NJC84 \| Improved cultivar \| G/G \| \| NJN76 \| Improved cultivar \| G/G \| \| NJN80 \| Improved cultivar \| G/G \| \| NJN89 \| Improved cultivar \| G/G \| \| Norman \| Improved cultivar \| G/G \| \| Okayama Wase \| Improved cultivar \| T/G \| \| Okinawa \| Improved cultivar \| G/G \| \| Okubo \| Improved cultivar \| ./. \| \| Pan Tao Huang Hou \| Improved cultivar \| T/G \| \| Pan Tao Wang \| Improved cultivar \| T/G \| \| Phillips \| Improved cultivar \| ./. \| \| Ping Ding You Pan Tao \| Improved cultivar \| G/G \| \| Ping Ding You Pan Tao \| Improved cultivar \| G/G \| \| Qian Nian Hong \| Improved cultivar \| T/G \| \| Qin Guang \| Improved cultivar \| T/G \| \| Qin Wang \| Improved cultivar \| T/G \| \| Red diamond \| Improved cultivar \| G/G \| \| Reddomun \| Improved cultivar \| T/G \| \| Redhaven \| Improved cultivar \| G/G \| \| Redskun \| Improved cultivar \| G/G \| \| Redtop \| Improved cultivar \| G/G \| \| Regina \| Improved cultivar \| G/G \| \| Robin \| Improved cultivar \| T/G \| \| Ruby prince \| Improved cultivar \| G/G \| \| Rui Guang 18 \| Improved cultivar \| T/G \| \| Rui Guang 2 \| Improved cultivar \| T/G \| \| Rui Guang 5 \| Improved cultivar \| T/G \| \| Rui Guang 7 \| Improved cultivar \| T/G \| \| Rui Pan 2 \| Improved cultivar \| T/G \| \| Rui Pan 3 \| Improved cultivar \| T/G \| \| Rutgers Redleaf \| Improved cultivar \| G/G \| \| Saotome \| Improved cultivar \| T/G \| \| Sha Hong \| Improved cultivar \| T/G \| \| Shaji 2 \| Improved cultivar \| T/G \| \| Shu Guang \| Improved cultivar \| T/G \| \| Shuang Feng \| Improved cultivar \| T/G \| \| Shuang Xi Hong \| Improved cultivar \| T/G \| \| Siberian C \| Improved cultivar \| G/G \| \| Sims \| Improved cultivar \| G/G \| \| Snow Queen \| Improved cultivar \| G/G \| \| Spring baby \| Improved cultivar \| G/G \| \| Spring gem \| Improved cultivar \| G/G \| \| Spring prince \| Improved cultivar \| G/G \| \| Springgold \| Improved cultivar \| G/G \| \| Springtime \| Improved cultivar \| G/G \| \| Sulian Pan Tao \| Improved cultivar \| T/G \| \| Summer Beauty \| Improved cultivar \| G/G \| \| Summer Grand \| Improved cultivar \| G/G \| \| Sunago Wase \| Improved cultivar \| T/G \| \| Sunhigh \| Improved cultivar \| G/G \| \| Sunred \| Improved cultivar \| G/G \| \| Super Crimson \| Improved cultivar \| G/G \| \| Triumph \| Improved cultivar \| G/G \| \| Tropicbeauty \| Improved cultivar \| G/G \| \| Troubadour \| Improved cultivar \| G/G \| \| Tsukuba 89 \| Improved cultivar \| T/G \| \| TX4B185LN \| Improved cultivar \| G/G \| \| Wan Bai Hua \| Improved cultivar \| T/T \| \| Wan Bai Mi \| Improved cultivar \| T/G \| \| Wan Mi \| Improved cultivar \| T/G \| \| Wei Yang 2 \| Improved cultivar \| T/G \| \| Wu yue Jin \| Improved cultivar \| T/G \| \| Xia Cui \| Improved cultivar \| T/T \| \| Xia Hui 1 \| Improved cultivar \| T/G \| \| Xia Hui 2 \| Improved cultivar \| G/G \| \| Xia Hui 5 \| Improved cultivar \| T/G \| \| Xin Bai Hua \| Improved cultivar \| T/G \| \| Xiwei gold \| Improved cultivar \| T/T \| \| Yan Guang \| Improved cultivar \| T/G \| \| Yan Hong \| Improved cultivar \| T/G \| \| Yang Zhou 431 \| Improved cultivar \| T/G \| \| Yang Zhou 531 \| Improved cultivar \| T/G \| \| Ying Shuang Hong \| Improved cultivar \| T/T \| \| Yu Bai \| Improved cultivar \| T/G \| \| Yumyeong \| Improved cultivar \| T/G \| \| Yun Shu 2 \| Improved cultivar \| T/T \| \| Zao Hong Xia \| Improved cultivar \| T/G \| \| Zao Hong Zhu \| Improved cultivar \| T/G \| \| Zao Hua Lu \| Improved cultivar \| T/G \| \| Zao Huang Pan Tao \| Improved cultivar \| T/G \| \| Zao Mei \| Improved cultivar \| T/T \| \| Zao Xia Lu \| Improved cultivar \| T/G \| \| Zao Yan \| Improved cultivar \| T/G \| \| Zhao Hui \| Improved cultivar \| T/G \| \| Zhao Xia \| Improved cultivar \| T/G \| \| Zheng Huang 2 \| Improved cultivar \| G/G \| \| Zheng Huang 3 \| Improved cultivar \| G/G \| \| Zhong 06-8-44 \| Improved cultivar \| T/G \| \| Zhong Pan Tao 11 \| Improved cultivar \| ./. \| \| Zhong You 4 \| Improved cultivar \| T/G \| \| Zhong You 5 \| Improved cultivar \| T/G \| \| Zhong You 7 \| Improved cultivar \| T/G \| \| Zhong You 9 \| Improved cultivar \| T/G \| \| Zhong You Pan 1 \| Improved cultivar \| T/G \| \| Zhong You Pan 2 \| Improved cultivar \| T/G \| \| Zhong You Pan 4 \| Improved cultivar \| T/G \| \| ZN Jin Hui \| Improved cultivar \| T/G \| \| ZN Jin Shuo \| Improved cultivar \| T/G \| | | |  |
|  |  |  |  |

| \| **Table S9. Details regarding the primers used in this study** \| \| \| \| --- \| --- \| --- \| \|  \|  \|  \| \| **Primers for marker variation of *PpTST1*** \| \| \| \| Name \| Forward primer \| Reverse primer \| \| PpTST1_primer1 \| ATTTAGTGCCCCCTGCTTCC \| CCACGTACCCTTGTCGGAAA \| \|  \|  \|  \| \|  \|  \|  \| \| **Primers for cloning coding region of *PpTST1*** \| \| \| \| Name \| Forward primer \| Reverse primer \| \| PpTST1_primer2 \| ATGAGGGGAGCTGTGATGGT \| TTACTCACTTTTGGCTGCGG \| \|  \|  \|  \| \|  \|  \|  \| \| **Primers for qRT-PCR analysis** \| \| \| \| Name \| Forward primer \| Reverse primer \| \| PpActin \| GATTCCGGTGCCCAGAAGT \| CCAGCAGCTTCCATTCCAA \| \| PpTST1_primer3 \| AAGGATTTAGTGCCCCCTGC \| CTTCTTTCCATCCCCGCCTT \| \| SlActin \| GTCCTCTTCCAGCCATCCAT \| ACCACTGAGCACAATGTTACCG \| \| SlTST1 \| ATGGTGAATTCATACAAGCTGC \| GCGATGCCAAAGTTAGAAAGAA \| \| Prupe.5G006400 \| GAGATGTGCGGAAAGGTTTATC \| GAACACCATGTTTCTGGTACAC \| \| Prupe.5G009600 \| GCTCAACAGGATCTTACTGGAA \| TCTTTGATCTTCTGCTTGCATG \| \|  \|  \|  \| \|  \|  \|  \| \| **Primers for vector construction** \| \| \| \| Name \| Forward primer \| Reverse primer \| \| PpTST1_Pri101-AN \| catatgcccgtcgaccccgggATGAGGGGAGCTGTGATGGTG \| agagttgttgattcagaattcTTACTCACTTTTGGCTGCGGC \| \| PpTST1_pART-CAM \| ggagaggacacgctcgagATGAGGGGAGCTGTGATGGT \| ttaaagcaggactctagaTTACTCACTTTTGGCTGCGG \| \|  \|  \|  \| \|  \|  \|  \| \| **Primers for qRT-PCR analysis of H(+)-ATPase genes, H(+)-pyrophosphatase genes , tonoplast dicarboxylate transporter genes and ALMT genes** \| \| \| \| Name \| Forward primer \| Reverse primer \| \| Prupe.1G082000 \| TGAAGAAATGGCTGGTATGGAT \| GAAGTGCACCTCTCTAATACCA \| \| Prupe.1G308100 \| CCAAAATTGGTTCCTTTAGGCA \| CGATACAATTGACAGTGACGTC \| \| Prupe.1G308300 \| CAAAGTGCTTCATCAGACAACA \| TGCAGTTCAGGATACCAAATCT \| \| Prupe.1G370100 \| GAGGTCAGATCCGATCAAGAAT \| GATGATAAGGCCGTAGATACCC \| \| Prupe.1G371400 \| TTCTAGTCAGAAACTTGGGGAC \| GACCTTACTCAAACCCACTGTA \| \| Prupe.1G460700 \| CCAAGCCCTTATTTTTGTCACA \| CACTTAGAATGTAGCGGATTGC \| \| Prupe.1G494900 \| AGACATGGCTTCTTCTACCTTC \| CTATGATGAGCCCATAGATCCC \| \| Prupe.2G128800 \| AATGACTGGTGATGGAGTGAAT \| AAAACCGAGCACAATACGTATG \| \| Prupe.3G024800 \| GAAGATGACCCTAGGAATCCTG \| TGAGAATATTCCAGCCGATGAA \| \| Prupe.3G029800 \| AATTGACCAGTCAGCTCTTACA \| TGTATGGGGAACATGACAATGA \| \| Prupe.3G091900 \| TTGCTCTGGGATACAAATCAGT \| CATCAGTTCTCTCACGGATTCT \| \| Prupe.3G095900 \| CAAACAAGCTGGAGGAAAAGAA \| TCTCAGAACCTTAGCTTTTGGT \| \| Prupe.3G169600 \| ATGTAGCTGGTGAGAACATCAA \| TCAATTGCTTTCACATAGGCAG \| \| Prupe.3G272500 \| AGCGTGGGATCTCATAATTGAT \| TACTTAATTCTCTCAGCCTGGC \| \| Prupe.3G312200 \| GTCGCAAAGATTGAGGATGATC \| ACCTAGGGACCACATAGTTAGT \| \| Prupe.4G009400 \| TGCATGTGCATCCACTCCTT \| AAGAAACGTAATGTTACCAAGTGT \| \| Prupe.4G009500 \| CTTGCTTCCAACAGGAACACC \| TGCTCCTGCAATAGCCAAAAA \| \| Prupe.5G040200 \| ATCTGCCTGCTAATGAGTCTAC \| TGGAAATCTGTAGCAGTGTCTT \| \| Prupe.5G062200 \| TCCAAAGACAGAGTTAAGCCAT \| ATGGTGCTAACTTGAAGGTACA \| \| Prupe.5G110600 \| GTGCCATAGACGTACACTCTTA \| AGCTCATGGTAGTTAGATTCCG \| \| Prupe.5G127200 \| AGCTCTGATTCTTCAATGGTCA \| GGAACTGCCTTTTTGGAATCTT \| \| Prupe.5G185000 \| CAGAGCAGATTCTAAACCTTGC \| TACACCAAGATCTAAAGCCCTC \| \| Prupe.5G220200 \| GTTTCGAAGGTGCTAAAGTGTT \| GATCAAGCACAGCTTCACTAAG \| \| Prupe.6G092300 \| TACCGAGATCCATACCCATTTG \| CTGTTAAGAAAGATCACCTGCG \| \| Prupe.6G144000 \| ACTCTACGAAGGTTTTGGTCTT \| ATACCTTGCCTTAATTTGGGGA \| \| Prupe.6G144100 \| CATCCAGAGCAACAAAGTTCAA \| GAATCGTGATGACAAGATGTGG \| \| Prupe.6G144200 \| TCATCACCTAGCTAGCCTATCT \| GGGCCAACTCCAATATTTCATC \| \| Prupe.6G194600 \| TATCAGCGCAAAGACAATGATG \| TCATGAAATGACTCCTGGTTGA \| \| Prupe.6G313800 \| TGTCAAGAACTGGTACCTCTTC \| GCAATGGCAAATACAGGAATGA \| \| Prupe.7G094400 \| CCCAACACTTAGCTAGTCTCTT \| GAGTCTTTCATGAGCCATTTCC \| \| Prupe.7G166200 \| TGTGATTATCAGCACTGGGATT \| CAAAGCTTCAGCAAAGATGAGA \| \| Prupe.7G250800 \| AAGAAGAAGAAGGCGGTAATGA \| CATATGTACATGGACGGCTACT \| \| Prupe.7G258000 \| GGACCAAGACTGTACGTATCAT \| CAAATCCATTCATTCCGACCAA \| \| Prupe.8G083200 \| ATAGTTGGTGATGCTGGTGTAA \| ATAGTTGGTGATGCTGGTGTAA \| \| Prupe.8G088600 \| TGGCTTTTGTTTCTGTACCATG \| TGAGACTGCTCCAAGTACAAAT \| \| Prupe.8G242400 \| GGTGCTACACTTAGTAAGGGTT \| GAAGATCATGGCTCCATAGTCA \| | | |
| --- | --- | --- | --- | --- | --- | --- | --- | --- | --- | --- | --- | --- | --- | --- | --- | --- | --- | --- | --- | --- | --- | --- | --- | --- | --- | --- | --- | --- | --- | --- | --- | --- | --- | --- | --- | --- | --- | --- | --- | --- | --- | --- | --- | --- | --- | --- | --- | --- | --- | --- | --- | --- | --- | --- | --- | --- | --- | --- | --- | --- | --- | --- | --- | --- | --- | --- | --- | --- | --- | --- | --- | --- | --- | --- | --- | --- | --- | --- | --- | --- | --- | --- | --- | --- | --- | --- | --- | --- | --- | --- | --- | --- | --- | --- | --- | --- | --- | --- | --- | --- | --- | --- | --- | --- | --- | --- | --- | --- | --- | --- | --- | --- | --- | --- | --- | --- | --- | --- | --- | --- | --- | --- | --- | --- | --- | --- | --- | --- | --- | --- | --- | --- | --- | --- | --- | --- | --- | --- | --- | --- | --- | --- | --- | --- | --- | --- | --- | --- | --- | --- | --- | --- | --- | --- | --- | --- | --- | --- | --- | --- | --- | --- | --- | --- | --- | --- | --- | --- | --- | --- | --- | --- | --- | --- | --- | --- | --- | --- | --- | --- | --- | --- | --- | --- | --- | --- | --- | --- | --- | --- | --- | --- | --- | --- | --- | --- | --- | --- | --- | --- |
|  |  |  |
